# NEXT-scASV: a Nextflow pipeline for allele-specific variant calling from single-cell RNA-seq data

**DOI:** 10.1093/gigascience/giag042

**Published:** 2026-04-06

**Authors:** Andrey Shevtsov, Andrey Buyan, Vladimir Nozdrin, Pavel Akhtyamov, Alexey Stupnikov, Georgy Meshcheryakov, Ivan V Kulakovskiy, Yulia A Medvedeva

**Affiliations:** Research Institute of Biotechnology, Russian Academy of Sciences, Moscow117312 Vavilova Street 7 Russia; Bioinformatics Group, AIRI, Moscow121170 Russia; Institute of Protein Research, Russian Academy of Sciences, Institutskaya 4142290Pushchino Russia; Faculty of Bioengineering and Bioinformatics, Lomonosov Moscow State University, MoscowLeninskie Gory 1119234 Russia; Department of Biomedical Physics, Moscow Center for Advanced Studies, Kulakova Street 20b1, Moscow123592 Russia; National Research University Higher School of Economics, 11 Pokrovksy Bulvar, Moscow 109028 Russia; Research Institute of Biotechnology, Russian Academy of Sciences, Moscow117312 Vavilova Street 7 Russia; Vavilov Institute of General Genetics, Russian Academy of Sciences, MoscowGubkina 3119991 Russia; Institute of Protein Research, Russian Academy of Sciences, Institutskaya 4142290Pushchino Russia; Institute of Protein Research, Russian Academy of Sciences, Institutskaya 4142290Pushchino Russia; Vavilov Institute of General Genetics, Russian Academy of Sciences, MoscowGubkina 3119991 Russia; Institute of Biochemistry and Genetics, Ufa Federal Research Centre of the Russian Academy of Sciences, Oktyabrya 71450054Ufa Russia; Research Institute of Biotechnology, Russian Academy of Sciences, Moscow117312 Vavilova Street 7 Russia; Mohamed Bin Zayed University of Artificial Intelligence, Masdar City, Abu Dhabi UAE

**Keywords:** allele-specific variants, single-cell RNA-seq, Nextflow, computational pipeline, regulatory variants

## Abstract

The rapid accumulation of single-cell sequencing data presents major computational challenges in reproducibility, scaling, and handling data characteristics such as sparsity and technical variations, which complicate even basic analyses. The next level of complexity is the allele-specific analysis, focused on identifying differential gene expression or regulation between homologous chromosomes by estimating the allelic imbalance of read counts at individual single-nucleotide variants. Here, we present NEXT-scASV, a scalable Nextflow pipeline for calling allele-specific variants (ASVs) from 5′ single-cell RNA sequencing data. NEXT-scASV automates the entire process—from read alignment and quality control to variant calling and statistical evaluation of the allelic imbalance—within a containerized environment, ensuring reproducibility and ease of deployment across platforms. NEXT-scASV is able to perform *de novo* ASV detection from single-cell sequencing data without prior genotyping. Its modular design allows for massive parallelization, efficiently handling the scale of modern atlas-level studies. We validate NEXT-scASV on a dataset of 135,000 peripheral blood mononuclear cells from 57 donors, demonstrating that it processes large-scale data efficiently, completing analysis in a week on a cluster with one node and 100 threads. Crucially, the pipeline reliably identifies ASVs even in rare cell populations (e.g., gdT GZMBhi and memory B IGHMhi cells), which remains elusive for bulk analyses. We also successfully detect allele-specific regulation of long non-coding RNAs and other lowly expressed, cell type-specific genes. Genes linked to detected ASVs show a high concordance (80%) with previously reported eQTLs. This strong validation confirms that NEXT-scASV produces biologically relevant results, making it a powerful tool for uncovering allele-specific regulation in large-scale, complex single-cell studies.

## Background

Genetic variation within regulatory elements represents a fundamental mechanism of phenotypic diversity and disease susceptibility [1, 2]. In diploid genomes, particular regulatory variants can be identified from comparison of allelic signals at homologous chromosomes, revealing allele-specific expression (ASE), allele-specific transcription factor binding (ASB), and allele-specific chromatin accessibility (ASA), representing different types of allele-specific variants (ASVs).

ASVs have been widely studied using bulk sequencing [3–7], which yields averaged signals across often heterogeneous cell populations. The averaging obscures cell type-specific effects, potentially diluting the signal from biologically meaningful, context-dependent regulation [8, 9]. The advent of single-cell sequencing (including scRNA-seq) provides the resolution necessary to interrogate ASVs and ASE within defined cell types [10, 11].

5′ scRNA-seq offers a distinct advantage for allele-specific analysis. By capturing the 5′ ends of transcripts, scRNA-Seq reads are strongly enriched in regulatory regions in the close vicinity to the transcription start sites (TSS), such as promoters and transcribed enhancers, physically linking genetic variants to the expression of their target genes on the level of individual reads [12]. This allows for direct and unambiguous assignment of regulatory effects compared to 3′ scRNA-seq protocols, which require imputation or statistical phasing. Furthermore, the high sensitivity afforded by cell type-specific analysis and the direct capture of 5′ ends opens the possibility of investigating allele-specific regulation in challenging genomic contexts. These include long non-coding RNAs (lncRNAs) and other lowly expressed or highly tissue-specific genes, which are often underrepresented in bulk tissue eQTL studies due to signal dilution across heterogeneous cell populations. Notably, the promoters of lncRNAs often contain highly specific genetic features [13] that are susceptible to disruption by genetic variants. This underscores the unique advantage of 5′ scRNA-seq and, by extension, our pipeline for directly interrogating the allele-specific mechanisms governing lncRNA expression.

Despite these advantages, single-cell RNA-seq data are sparse: Most variants are covered by few reads per cell, and many cells show dropout at a given locus. To increase power, some single-cell allele-specific approaches, therefore, rely on different aggregation strategies, most commonly at the gene level, where allelic counts are summed across sites within a gene and tested for gene-level ASE [14, 15]. In this work, we focus on variant-level ASVs rather than gene-level aggregation, because regulatory effects are often driven by specific nucleotide changes. A single variant can disrupt a transcription factor motif, alter promoter or enhancer activity, or create a splice-altering event, leading to a mechanistic and directly interpretable signal. Variant-level results also enable direct overlap with external catalogs of regulatory variants, and they avoid masking situations where multiple variants within the same gene have different, context-dependent effects.

Several single-cell methods have been proposed for allele-specific analysis. SCALE [15] is designed for gene-level ASE modeling from sparse scRNA-seq counts. scBASE [14] integrates allele-specific signals with phased haplotypes, which typically requires phased genotypes. Tools that can operate at the variant level using allelic counts: DAESC [16] and scDALI [17]. DAESC is formulated to detect differential allelic imbalance between groups or conditions, whereas scDALI supports tests for both differential effects (heterogeneous mode) and shared allele-specific effects (homogeneous mode), as well as a joint setting that combines information across modes.

However, analyzing scRNA-seq data for ASE is a multi-faceted computational challenge, and very few existing tools allow for this kind of analysis [18]. The process involves numerous steps: demultiplexing, quality control, read alignment and deduplication, variant calling, allelic read counting, and finally, statistical modeling to pinpoint the single-nucleotide variants (SNVs) with the significant allelic imbalance. Each step requires specific software tools with complex dependencies; orchestrating such a workflow with custom scripts is fragile, difficult to parallelize, and notoriously hard to reproduce, creating a significant barrier to reliable discovery. Workflow management systems such as Nextflow [19] have emerged to solve these problems and gained popularity. Nextflow enables the creation of portable, scalable, and reproducible pipelines by abstracting away complex job scheduling, managing software dependencies through containers (Docker [20], Singularity [21]), and providing built-in mechanisms for logging and resuming failed runs.

Here, we present NEXT-scASV (Nextflow pipeline for ASV calling), a comprehensive and robust workflow designed specifically for discovery of ASVs from 5′ scRNA-seq data. NEXT-scASV integrates established tools for alignment (HISAT2 [22]), reads deduplication (umi_tools [23]), variant calling (bcftools mpileup [24]), reference bias correction (WASP [25]), and advanced statistical modeling (MIXALIME [26]). We demonstrate its performance on a large dataset of human peripheral blood mononuclear cells (PBMCs). The high sensitivity afforded by cell type-specific analysis and the direct capture of 5′ ends opens the possibility of investigating allele-specific regulation in rare cell populations or challenging genomic contexts, such as in long non-coding RNAs (lncRNAs) and other lowly expressed or highly tissue-specific genes, which are often underpowered in bulk tissue eQTL studies. By providing a standardized, containerized, and parallelized solution, NEXT-scASV lowers the barrier for performing sophisticated allele-specific analyses in large-scale single-cell studies.

## Materials and methods

### Pipeline design and implementation

NEXT-scASV is implemented in Nextflow (DSL2) with a modular structure where each processing stage (or subflow) is defined as a separate, reusable module. The pipeline execution is managed through a central configuration file, defining input data paths, sample metadata, and important parameters for each module. All software dependencies are packaged into Docker, supporting reproducibility across different computing environments.

The pipeline requires two primary inputs from the user:

1. A metadata JSON file linking sample identifiers to the paths of the corresponding FASTQ files.
2. A barcode-to-group assignment CSV file containing columns for *barcode, sample*, and *group* (e.g., a cell type). This file is essential for splitting the data and performing group-level analyses.

**Figure 1:**
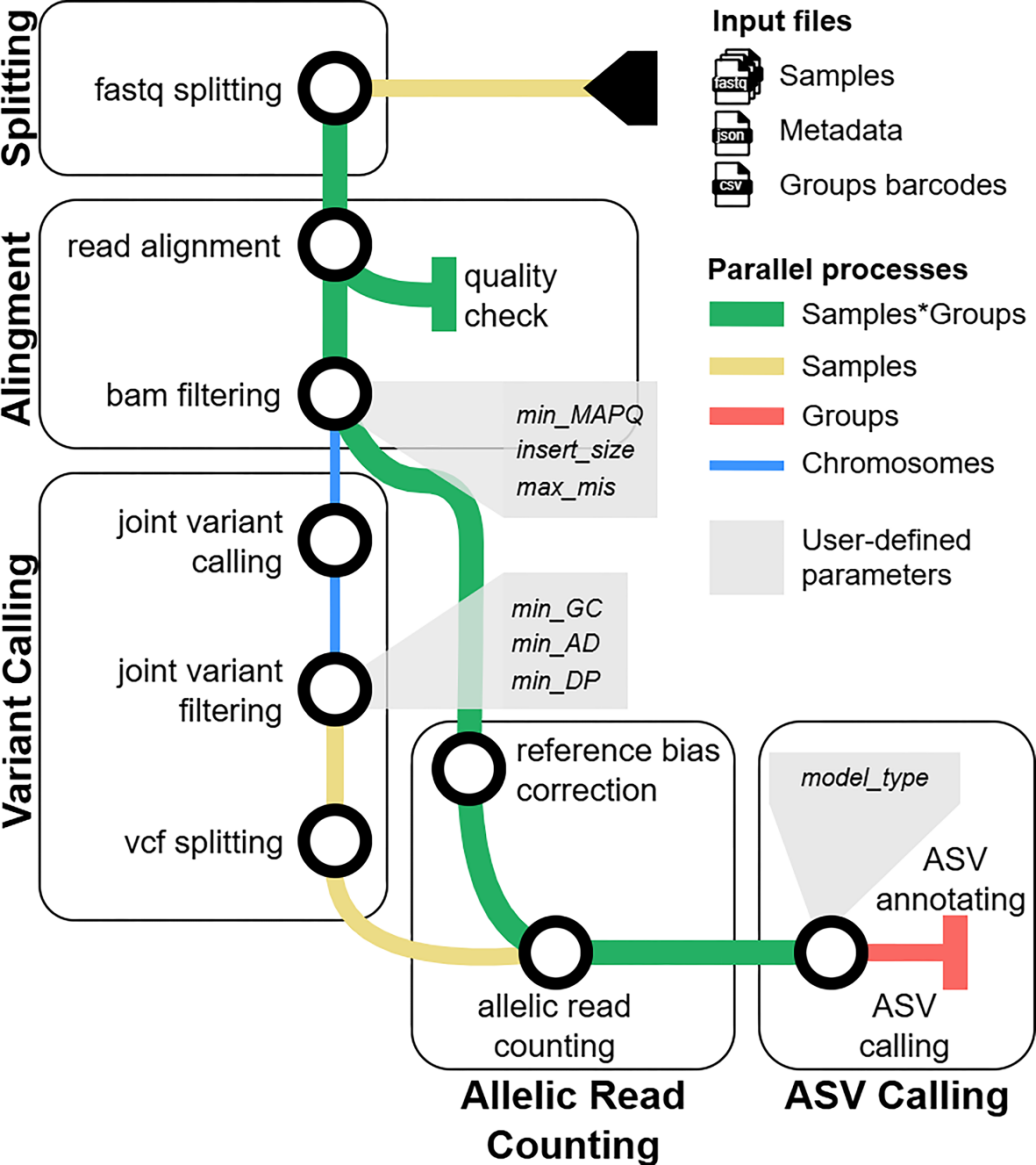
Schematic overview of the NEXT-scASV pipeline. Rounded rectangles represent the five main subflows. Circles represent individual modules within a subflow. The color and thickness of the arrows indicate the data aggregation strategy. Grayscale rectangles highlight important user-configurable parameters.

The workflow consists of five main subflows, illustrated in Fig. 1: (i) data splitting, (ii) alignment and filtering, (iii) variant calling, (iv) allelic read counting, and (v) ASV calling.

### Splitting subflow

The initial step employs a custom Python script to split the raw FASTQ files based on the provided barcode-group assignments. Each input sample (e.g., one donor) is split into multiple FASTQ files, one for each assigned group (e.g., a cell type). This strategy, while increasing the number of subsequent tasks, is the cornerstone of the pipeline’s parallelization, allowing downstream steps to process each sample × group combination independently and in parallel.

### Alignment subflow

Each split FASTQ file is then processed individually. First, the reads are trimmed to remove adapters and low-quality bases using cutadapt v5 [27]. Processed reads are then aligned to the reference genome (e.g., GRCh38) using HISAT2, which efficiently handles splice-aware alignment of RNA-seq data.

We selected HISAT2 over STAR due to its superior speed, a critical factor when processing large single-cell datasets with multiple donors and cell types. This choice does not compromise accuracy: On a dataset with a natural human polymorphism rate of 0.001, both aligners delivered nearly identical performance, achieving near 100% precision and >95% recall [28].

Following alignment, PCR duplicates are marked and removed using umi_tools dedup [23] to mitigate technical artifacts. The resulting BAM files are rigorously filtered to retain only high-quality alignments. For our testing, we set the following values for the user-defined parameters: mapping quality (MAPQ) ≥ 10, a maximum of 2 mismatches, and an insert size ≤750 bp.

### Variant calling subflow

To call heterozygous SNVs, as in the default MIXALIME workflow [26], the pipeline processes the filtered BAM files from each sample × group combination using bcftools mpileup and bcftools call [24]. Variant calling is performed independently for each chromosome (1–22) to maximize parallelization. The initial call set is stringently filtered to ensure high-confidence heterozygous sites: read depth (DP) ≥ 10, genotype quality (GQ) ≥ 50, and a minimum of 5 reads supporting each allele (AD ≥ 5). At the vcf splitting step, jointly called variants are split by samples, and each sample vcf file is also filtered by a minimum of 5 reads supporting each allele.

The choice of variant caller determines the initial set of candidate single-nucleotide polymorphisms (SNPs) and therefore establishes the upper bound for sensitivity and accuracy in downstream analysis. However, in our pipeline, the impact of the caller is mitigated through several subsequent steps. We primarily use the caller for initial SNP discovery, after which we apply rigorous variant-level filters, including genotype quality (GQ), read depth (DP), and allelic depth (AD). Additionally, we implement read-level filters before counting, such as MAPQ, edit distance (NM), and exclusion of QC-failed reads using filter_reads.py and samtools (flag 512). Crucially, the final allele-specific expression (ASE) signal is derived through direct allele-specific read counting from BAM files via count_tags_pileup.py, rather than relying on caller-derived quality scores. Furthermore, variants are called at the patient level, which leverages information across related samples to address the sparse coverage typical of single-cell RNA-seq data. As a result, the final ASE estimates are substantially less dependent on the specific variant caller than in workflows where caller outputs directly determine allelic estimates. For this study, we selected bcftools as our variant caller, as it offers a simpler, faster, and more computationally lightweight solution while still providing robust candidate variants suitable for downstream ASE analysis.

### Allelic read counting subflow

A critical challenge in ASV analysis is reference allele mapping bias, where reads containing alternative alleles map with lower confidence. To correct this, the pipeline integrates the WASP tool [25]. For each potential variant site, WASP identifies reads overlapping the site, swaps the alleles, remaps these modified reads, and filters out the original reads that fail to remap correctly. This process generates a corrected set of alignments devoid of reference bias. A Python script [29] then parses these corrected BAM files to count the number of reads supporting the reference and alternative alleles for every variant in every sample × group combination, producing an extensive table of allelic read counts for statistical testing.

### ASV calling subflow

The final step identifies the sites with the significant allelic imbalance using the MIXALIME framework [26]. MIXALIME fits probabilistic models to the allelic read counts, accounting for data sparsity and over-dispersion inherent in scRNA-seq. We employed the Beta-Negative Binomial (BetaNB) model, which is the most conservative yet reliable for modeling over-dispersed count data. The tool fits model parameters and calculates *P*-values for the deviation from the expected balanced allelic read counts (i.e., 0.5 ratio). *P*-values are then aggregated with the Mudholkar–George *logitp* method [30] across hierarchical groups (e.g., within a cell type lineage) to increase statistical power. False discovery rate (FDR) correction is applied (Benjamini–Hochberg), and the significant ASVs are called at FDR < 0.05. MIXALIME also generates extensive quality control plots, including goodness-of-fit metrics like RMSEA (Root Mean Square Error of Approximation) [31]. Significant ASVs are functionally annotated by overlapping their genomic coordinates with regulatory databases such as GTEx for eQTLs and ADASTRA for ASB events [2, 32], providing immediate biological context and an additional layer of validation against known regulatory SNPs (rSNPs).

### Benchmarking dataset

The pipeline was tested on data from the Asian Immune Diversity Atlas (AIDA) [33], a comprehensive resource of scRNA-seq data from PBMCs of 619 healthy donors. A random subset of 57 donors (23 male, 34 female, aged 25–40) of South Korean ancestry was selected for this benchmarking study to balance computational feasibility with statistical power. The dataset comprised 17 major immune cell types, as annotated in the original paper.

### Computational resource profiling

The pipeline was executed on a high-performance computing cluster managed by the SLURM workload manager, with a constrained limit of 1 node with 100 threads and 15 TB of RAM. Resource usage (CPU time, peak memory, disk I/O) for each process was meticulously recorded using Nextflow’s built-in tracing capabilities. The total execution time was calculated under three scenarios: the sum of all process times (emulating a single CPU, lower bound), the duration of the longest process chain (emulating infinite parallelization, upper bound), and the actual wall time under the 100 threads constraint.

### Biological validation against eQTL data

To assess the biological relevance of the discovered ASVs, we compared our results against the eQTL analysis published in the original AIDA study. For each significant ASV, we identified its nearest TSS. The resulting list of ASV-associated genes was intersected with the list of genes identified as significantly associated with eQTLs (eGenes) in the AIDA cohort (FDR < 0.05).

### Allele-specific analysis with scDALI and DAESC

We ran scDALI and DAESC in two input modes. First, we used a pseudobulk setup consistent with our pipeline, where allelic counts were aggregated for each sample × group combination and used as input for the models. Second, we used a true single-cell setup: At the allelic counting stage, we computed reference and alternative counts for each barcode, yielding allele-count matrices for ∼70,000 cells, which were then used as input for scDALI and DAESC.

For scDALI, we applied additional filtering thresholds: minimum SNPs per cell = 50, minimum cells per SNP = 50, minimum non-zero ALT cells = 5, and minimum non-zero REF cells = 5. scDALI was run in the joint mode (heterozygous + homozygous), which provides a test that is closer in spirit to overall allelic imbalance detection while still leveraging single-cell variation.

For DAESC, we used minimum SNPs per cell = 50 and minimum cells per SNP = 50, and ran the model with the following parameters: num_iter = 50, min_iter = 20, and max_optim = 10.

### Variant calling with cellsnp-lite

As an alternative to the default variant-calling branch based on bcftools mpileup/call, we generated variants with cellsnp-lite [34]. In this mode, the pipeline takes the processed, coordinate-sorted, and indexed BAM files and runs cellsnp-lite in Mode 2b, using the configured cell barcode/UMI tags (e.g., CB/UB when enabled). cellsnp-lite outputs per-locus reference/alternative allele counts, which are then used as the variant set for the downstream counting/ASE steps.

## Results

### NEXT-scASV pipeline enables scalable and reproducible analysis

The NEXT-scASV pipeline integrates a complex series of tools into a cohesive, automated workflow (Fig. 1). Its modular Nextflow DSL2 implementation allows users to easily start, stop, and resume analyses, a critical feature for long-running computations. The use of containers eliminates “dependency hell” and ensures identical results regardless of the host system.

A key design feature is the aggressive data splitting at the outset. While this strategy generates a large number of intermediate files (∼15 TB for this study), it unlocks massive parallelization, transforming a computationally prohibitive task into a manageable one. The configuration of the pipeline allows fine-grained control over resource allocation for each step and provides options for cleaning up intermediate files to conserve disk space after successful completion of individual stages.

### Pipeline performance and resource utilization

**Figure 2:**
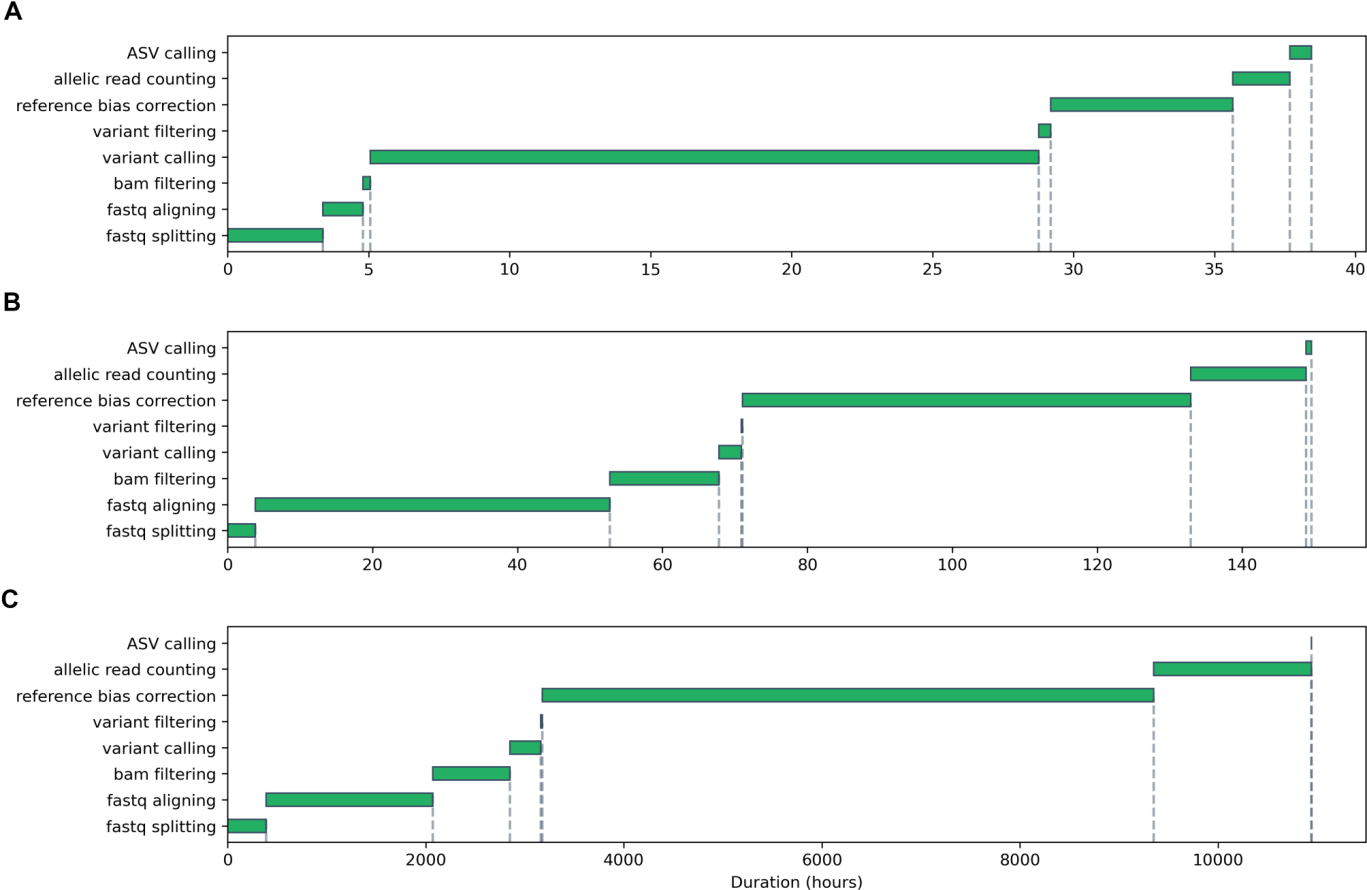
Pipeline execution time under different parallelization scenarios. (A) Mean process duration per stage, representing the ideal upper bound with infinite parallelization. (B) Actual wall-time duration with a limit of 100 concurrent CPUs. (C) Cumulative sum of all process times, representing the lower bound of sequential execution on a single CPU.

**Figure 3:**
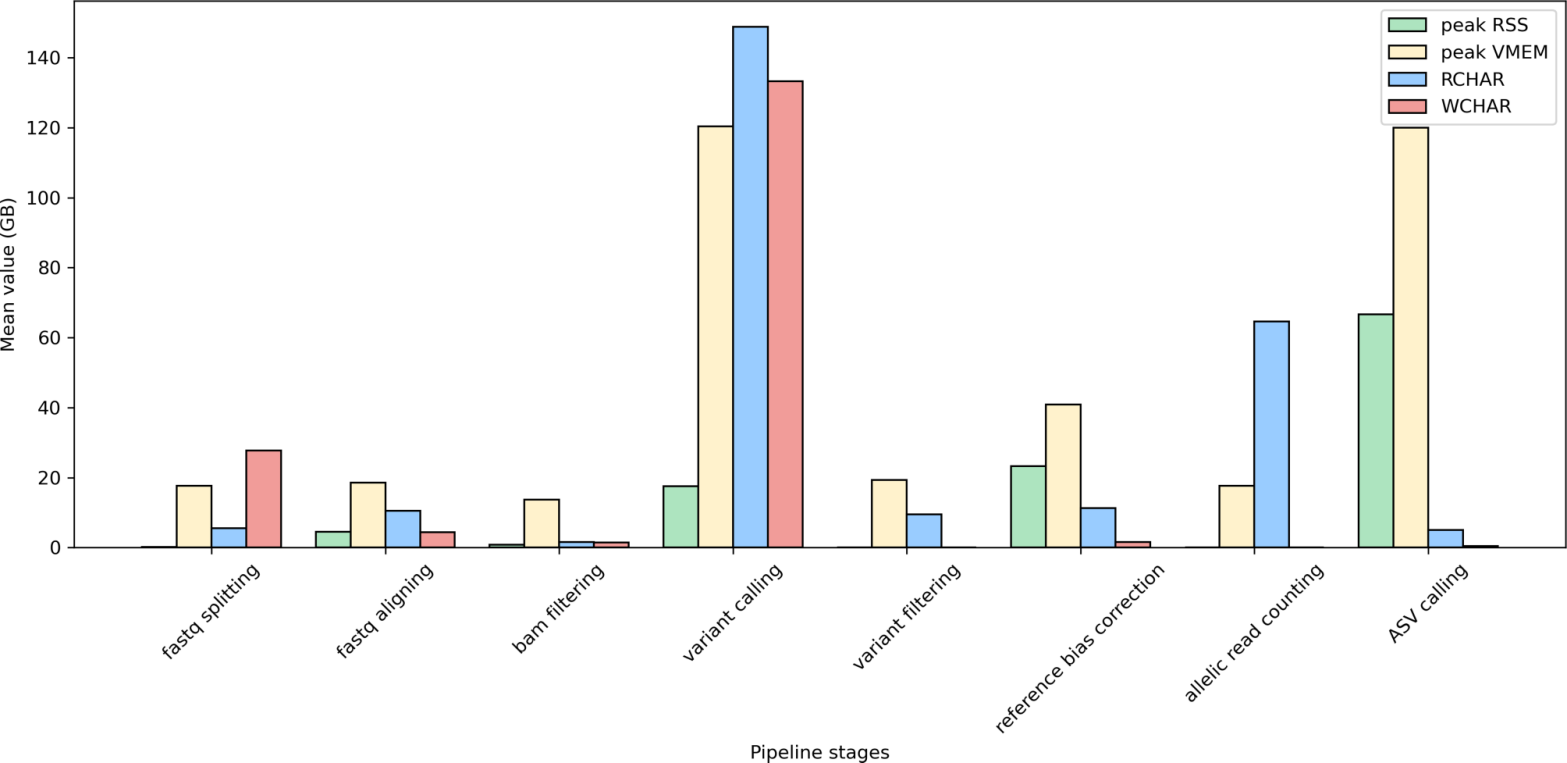
Mean computational resource utilization per process for each major pipeline stage. Metrics shown include: peak RSS (peak Resident Set Size, the maximum amount of physical memory a process has used at any point during its execution), peak VMEM (peak virtual memory usage, includes swap), RCHAR (volume of data read from storage), and WCHAR (volume of data written to storage).

We evaluated the computational cost of processing the 57-donor PBMC dataset. Under a realistic constraint of 100 threads, the pipeline completed in ∼7 days (Fig. 2B). Profiling the resource usage revealed distinct patterns for different stages (Fig. 3). The alignment stages (“fastq aligning” and “reference bias correction”) were the most time-consuming but scaled efficiently with the number of available CPUs. In contrast, the “Call variants” stage, split into 22 parallel chromosome-level tasks, represents a parallelization bottleneck; its duration is fixed by the longest chromosome job and cannot be reduced by adding more CPUs beyond this point. This stage also exhibited the highest memory demand, with a peak virtual memory usage of ∼120 GB per process and intensive disk I/O (reading/writing >140 GB per process), highlighting the need for substantial memory and fast storage on compute nodes.

Theoretical estimates illustrate the necessity of parallelization: running the workflow sequentially on a single CPU would take >400 days (Fig. 2C), while perfect parallelization could reduce this to under a day (Fig. 2A). Our real-world scenario (100 CPUs) strikes a practical balance, demonstrating that NEXT-scASV can process atlas-scale data within a reasonable timeframe on a high-performance computing cluster typically used in data-intensive biomedical research.

### Quality control and model validation

The statistical analysis using MIXALIME passed the basic quality check. Particularly, the goodness-of-fit, measured by RMSEA for all fitted models, was excellent, falling well below the recommended threshold of 0.05 (Supplementary Fig. S1A), indicating that the chosen BetaNB model adequately captured the overall distribution of allelic read counts.

As an orthogonal validation, we examined the fraction of discovered ASVs annotated in external databases of rSNPs (GTEx eQTLs and ADASTRA ASBs). We observed that the proportion of annotated variants increased monotonically along with higher thresholds for the FDR significance (Supplementary Fig. S1B). This trend is biologically expected—stronger, more reproducible functional variants are more likely to be cataloged in existing databases—and provides independent support for the correctness of our statistical calls.

### Caller comparison

To provide an empirical benchmark, we compared bcftools with cellsnp-lite. Сellsnp-lite reported more candidate variants (Supplementary Fig. S3A), but most additional variants did not pass downstream ASV significance testing in MixALime (Supplementary Fig. S3B). This suggests that cellsnp-lite is more sensitive to low-coverage variants, but this increased sensitivity does not translate into a clear advantage in terms of final significant ASVs. At the same time, each caller contributed a little fraction of caller-specific variants (Supplementary Fig. S3B).

### ASV tools comparison

We evaluated two alternatives to MixALime: DAESC and scDALI. When we ran them in the pseudobulk mode (the same way as in our pipeline), almost all variants detected by DAESC and scDALI were also detected by MixALime (Supplementary Fig. S4A). In this setting, MixALime identified many more ASVs. A likely reason is that MixALime is designed to detect allelic imbalance itself, while DAESC and scDALI are primarily designed to test differences between groups or cell states. Next, we ran DAESC and scDALI on true single-cell allele counts. In this case, the number of detected ASVs increased strongly (DAESC: 31 → 188; scDALI: 23 → 130), and unique variants appeared (Supplementary Fig. S4B). scDALI overlapped with MixALime more than DAESC, consistent with the idea that scDALI in the joint (homozygous + heterozygous) mode is conceptually closer to MixALime. DAESC targets allele-specific effects that vary across cell states or conditions rather than overall allelic imbalance from MixALime, so it should be considered complementary to MixALime rather than a direct alternative. Finally, ASVs reported by all three tools showed substantial overlap with known regulatory variant resources, including GTEx cis-eQTLs and ADASTRA (Supplementary Fig. S4C and D), indicating that each method recovers a meaningful fraction of previously reported regulatory variants. scDALI and DAESC application details are provided in the “Methods” section: allele-specific analysis with scDALI and DAESC.

### NEXT-scASV uncovers allele-specific events in rare cell populations and challenging genomic conditions

A major advantage of single-cell resolution is the ability to probe allele-specific regulation in rare cell types. We leveraged MIXALIME’s ability to aggregate *P*-values across different hierarchical levels (e.g., within T-cell subsets before aggregating to all T cells) to enhance statistical power at different levels of resolution. As expected, aggregating data to broader cell types (e.g., all T cells, all PBMCs) increased the total number of detectable ASVs due to greater statistical power from larger read counts (Supplementary Fig. S2).

**Figure 4:**
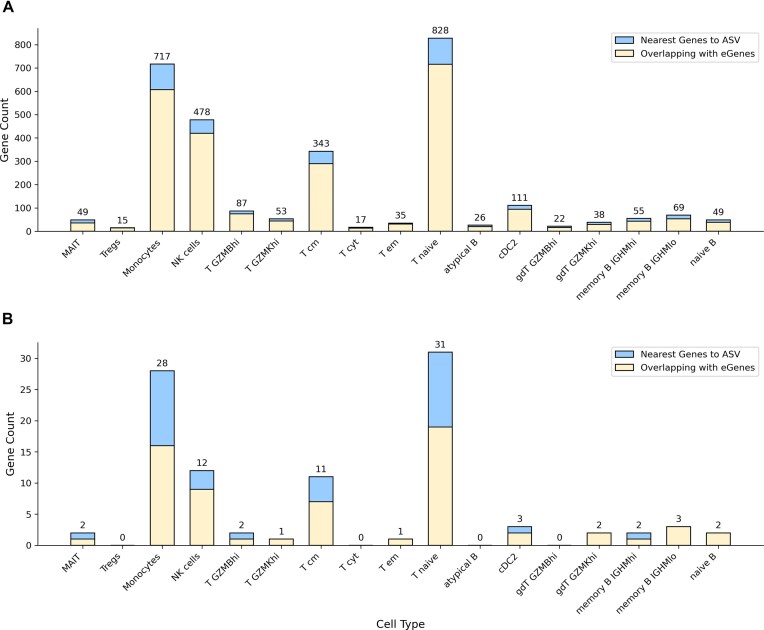
Validation of discovered ASVs by comparison against eQTLs analysis. For each cell type, the bar shows the total number of genes associated with significant ASVs. The shaded segment indicates the subset of these genes that were also reported as eGenes (FDR < 0.05) in the original cohort eQTL study. (A) All genes and (B) non-coding RNAs.

The sensitivity gains from analyzing particular cell types extend beyond discovering effects in rare populations to also detecting ASE of genes with low or cell type-specific expression. This includes challenging targets, such as long non-coding RNAs (lncRNAs), which are often expressed at low levels and whose regulation is highly context-dependent. In our case study, we identified significant ASVs impacting the expression of several non-coding RNAs. Fractions of the detected ASV impacting non-coding RNAs are the same as for all genes (Fig. 4B). Importantly, we found in total 19 long non-coding RNAs, some of them are well-studied regulators such as P53 Induced Transcript LINC-PINK or NLRP3 inflammasome regulator LINC00989, and also a number of less-studied RNAs (e.g., LINC02723, LINC01220, LINC01871, LINC01679, LINC02273, LINC01619), that may be included in immune cells regulation. The ability to probe allele-specific regulation of such genes provides a new avenue for understanding the functional impact of genetic variation in the non-coding genome.

### High concordance with independent eQTL analysis validates biological relevance

To validate the biological significance of our findings, we compared the set of genes linked to the detected ASVs with those reported as significantly associated with eQTLs (eGenes) in the AIDA cohort study [33]. This comparison showed a very high level of agreement: On average, 82% (± 6% across cell types) of the genes associated with ASVs were also identified as eGenes (Fig. 4 and Supplementary Fig. S5). This strong concordance with an orthogonal analysis performed on a larger cohort with genotyping data provides robust evidence that NEXT-scASV reliably identifies biologically genuine cis-regulatory variants.

The remaining ∼18% of ASV-linked genes not reported as eGenes likely represent a mix of false positives and, more interestingly, true cell type-specific regulatory events. These could either be effects that are too weak, narrow context-specific, or dependent on cellular environments not captured in the bulk-tissue eQTL analysis, pointing to potential novel biology uncovered by our single-cell approach.

## Discussion

We have developed NEXT-scASV, a scalable and reproducible pipeline for the detection of ASVs from 5′ scRNA-seq data. Its containerized implementation ensures that the ASV calling is reproducible across different computing platforms, a cornerstone of robust scientific practice. Our benchmarking on data from 57 individuals (135,000 cells) demonstrates that NEXT-scASV is capable of processing large datasets in a feasible timeframe on a mid-sized compute cluster. The resource analysis provides a valuable guide for other groups planning similar studies. The pipeline performance is not just technical: Its results agree well with existing rSNP annotations, showing 80% concordance with established eQTLs, while also revealing cell type-specific effects that are invisible to bulk approaches.

A key design philosophy was to enable *de novo* discovery without requiring external genotyping data, making the pipeline widely applicable to standard 5′ scRNA-seq study designs. The integration of WASP mitigates reference mapping bias, a known source of false positives in ASE studies, and the use of MIXALIME provides a statistically rigorous framework capable of handling the overdispersion in allelic counts arising due to noise and sparsity of single-cell data.

The ability of NEXT-scASV to detect signals in rare cell types, such as gdT GZMBhi cells, is particularly exciting. It opens the door to investigating allele-specific regulation in rare cell populations involved in disease, development, and immune response, which have been largely inaccessible. An exciting implication of our approach is its potential to illuminate allele-specific regulation in genomic regions that have been historically difficult to study. The combination of cell type-specific resolution and sensitivity makes NEXT-scASV particularly suited to investigate ASE of lncRNAs and other lowly expressed genes. These categories are often poorly tagged in bulk tissue eQTL studies due to their low expression and high cell type specificity. By isolating the relevant cell type, our pipeline can overcome this limitation, offering a powerful strategy to ascribe function to genetic variants associated with lncRNAs and other elusive elements of the regulome.

The modular, DSL2-based architecture of Nextflow is a core strength of NEXT-scASV, ensuring it is not a static tool but a flexible framework for future methodological developments. The pipeline can be readily adapted to other single-cell modalities that probe cis-regulatory activity, such as scATAC-seq for identifying ASA or multiome assays that simultaneously measure chromatin accessibility and gene expression. This would primarily involve swapping the alignment and initial processing modules while leveraging the same robust downstream variant calling and statistical analysis framework. Furthermore, the modular design simplifies the process of incorporating new best practices, such as advanced filters for technical artifacts or novel statistical models for allelic imbalance, ensuring the pipeline remains at the forefront of the field without requiring a complete rebuild. This adaptability makes NEXT-scASV a lasting resource for the community, capable of evolving alongside rapidly advancing single-cell technologies.

We compared the key step of our workflow—detecting allele-specific events with MixALime—to methods with closely related functionality (scDALI and DAESC). Results suggest that scDALI provides a conservative subset of MixALime calls, while DAESC can expand the analysis by highlighting variants with context-dependent allelic effects.

While NEXT-scASV provides a robust framework for allele-specific analysis, several limitations should be mentioned. First, the pipeline’s current implementation requires a predefined cell type annotation provided by the user via a barcode-group assignment file. It is therefore dependent on the accuracy and resolution of this external annotation. While the hierarchical aggregation in MIXALIME mitigates this to some degree, integrating the pipeline with a more advanced probabilistic cell typing could be a valuable future direction.

Second, the computational footprint, particularly the storage requirements for intermediate files (∼15 TB for this study) and the high I/O load during the variant calling stage, can be prohibitive for extra large studies without access to high-performance computing infrastructure with fast and capacious storage.

Third, the variant calling step relies solely on the scRNA-seq data itself. While this *de novo* approach is a key feature that increases broad applicability, it may be less sensitive than methods that incorporate external genotype information from array data or whole-genome sequencing. Finally, the statistical power to detect ASVs is inherently constrained by the number of expressed reads covering a heterozygous SNP in a given cell type. While aggregation of related cell types helps, very rare cell types or extremely lowly expressed genes will remain challenging to analyze, a limitation inherent to all current scRNA-seq analyses.

In conclusion, NEXT-scASV provides the community with a validated, scalable, and reproducible solution for deciphering allele-specific regulation from 5′ scRNA-seq data. By transforming a complex, multi-stage analysis into an automated and portable workflow, it empowers researchers to move beyond logistical challenges and focus on biological discovery. We demonstrate that it reliably recovers known regulatory biology while uniquely enabling the exploration of genetic effects in rare cell populations and understudied genomic elements. As single-cell atlas projects continue to expand, NEXT-scASV stands as a critical tool for unlocking the functional impact of genetic variation across the full spectrum of cellular diversity in health and disease.

## Availability of source code and requirements

Project name: NEXT-scASV

Project homepage: https://github.com/MedvedevaLab/NEXT-scASV

License: MIT license

Operating system(s): Linux (CentOS/Ubuntu)

Package management: Conda

Programming language: Nextflow, Python, Bash

Hardware requirements: High-performance computing (HPC) cluster recommended. Requirements depend on input data size. Tested on SLURM cluster with 100 CPUs and 100 GB RAM. Minimum 16 GB RAM for small test datasets.

RRID:SCR_027470

## Additional files

**Packages versions:** python=3.10.0; Cutadapt=5.0; HISAT2=2.2.1; UMI-tools=1.1.6; Bcftools=1.21; WASP=v0.4.3; MIXALIME==2.27.3.

**Supplementary Fig. S1:** Quality control of the ASV detection. (A) Root Mean Square Error of Approximation (RMSEA) for all fitted MIXALIME models. The dashed line indicates the desired threshold of 0.05. (B) Fraction of significant ASVs that can be annotated in the GTEx (eQTL) and ADASTRA (ASB) databases as a function of the FDR threshold.

**Supplementary Fig. S2:** Number of significant ASVs detected at different levels of hierarchical cell type aggregation. The number in the green shape defines the number of found ASV, the yellow shape includes the number of unique found ASV per cell type (сounts represent events unique to a node after subtracting all events from its parent nodes, so the nodes show only events not classified into any sub-type, highlighting the gain from broader aggregation). (A). Main PBMC hierarchy without T cells branching. B. T cells branching.

**Supplementary Fig. S3:** Number of variants that callers can detect. (A) Barplot with variants counts after calling and filtering, with green bar refers to both callers detections, yellow bar with unique bcftools mpileup variants counts and blue bars with only cellsnp-lite detected variants. (B) Significant ASV after each caller.

**Supplementary Fig. S4:** Intersections of significant ASV (FDR ≤ 0.05) identified by DAESC, scDALI, and MixALime. (A) All three tools use pseudobulk allelic counts (sample × cell group) as input. (B) DAESC and scDALI use single-cell allelic counts as input; MixALime uses pseudobulk counts. (C) Subset of significant ASV from B that overlap with cis-eQTLs in the GTEx database. (D) Subset of significant ASVs from B that overlap with regulatory variants in the ADASTRA database.

**Supplementary Fig. S5:** Validation of discovered ASVs by comparison against eQTLs analysis. For each cell type, the bar shows the total number of genes associated with significant ASVs (blue). The yellow segment indicates the subset of these genes that were also reported as eGenes (FDR < 0.05) in the original cohort eQTL study. The green segment represents the number eGens that are not intersected with the found ASVs.

## Supplementary Material

giag042_Supplemental_Files

giag042_Authors_Response_To_Reviewer_Comments_original_submission

giag042_GIGA-D-25-00386_original_submission

giag042_GIGA-D-25-00386_Revision_1

giag042_Reviewer_1_Report_original_submission Reviewer 1 -- 11/20/2025

giag042_Reviewer_1_Report_revision_1 Reviewer 1 -- 3/16/2026

giag042_Reviewer_2_Report_original_submission Reviewer 2 -- 11/24/2025

giag042_Reviewer_3_Report_original_submission Reviewer 3 -- 11/26/2025

giag042_Reviewer_3_Report_revision_1 Reviewer 3 -- 3/9/2026

## Data Availability

We used the data from the AIDA datasets and it is available via the HCA Data Portal [35]. The Supplementary material is deposited in GigaDB [36].
