# Supplementary figures and images for: NEXT-scASV: a Nextflow pipeline for allele-specific variant calling from single-cell RNA-seq data
Source: Gigascience. 2026 Apr 6;15:giag042. doi: 10.1093/gigascience/giag042 (PMC13148397; doi:10.1093/gigascience/giag042)

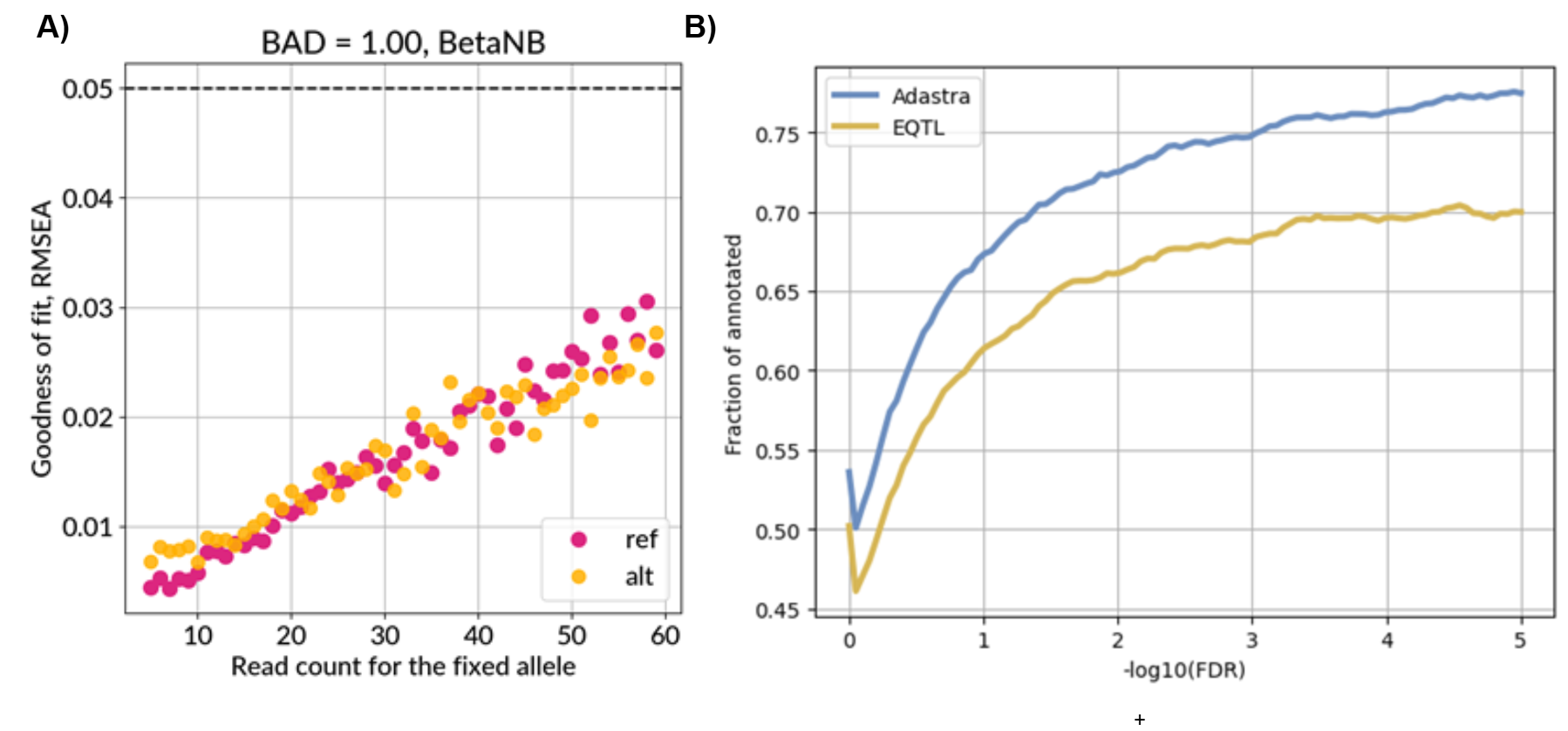

Supplement: giag042_Supplemental_Files [file giag042_supplemental_files.zip › fig_s1.png]

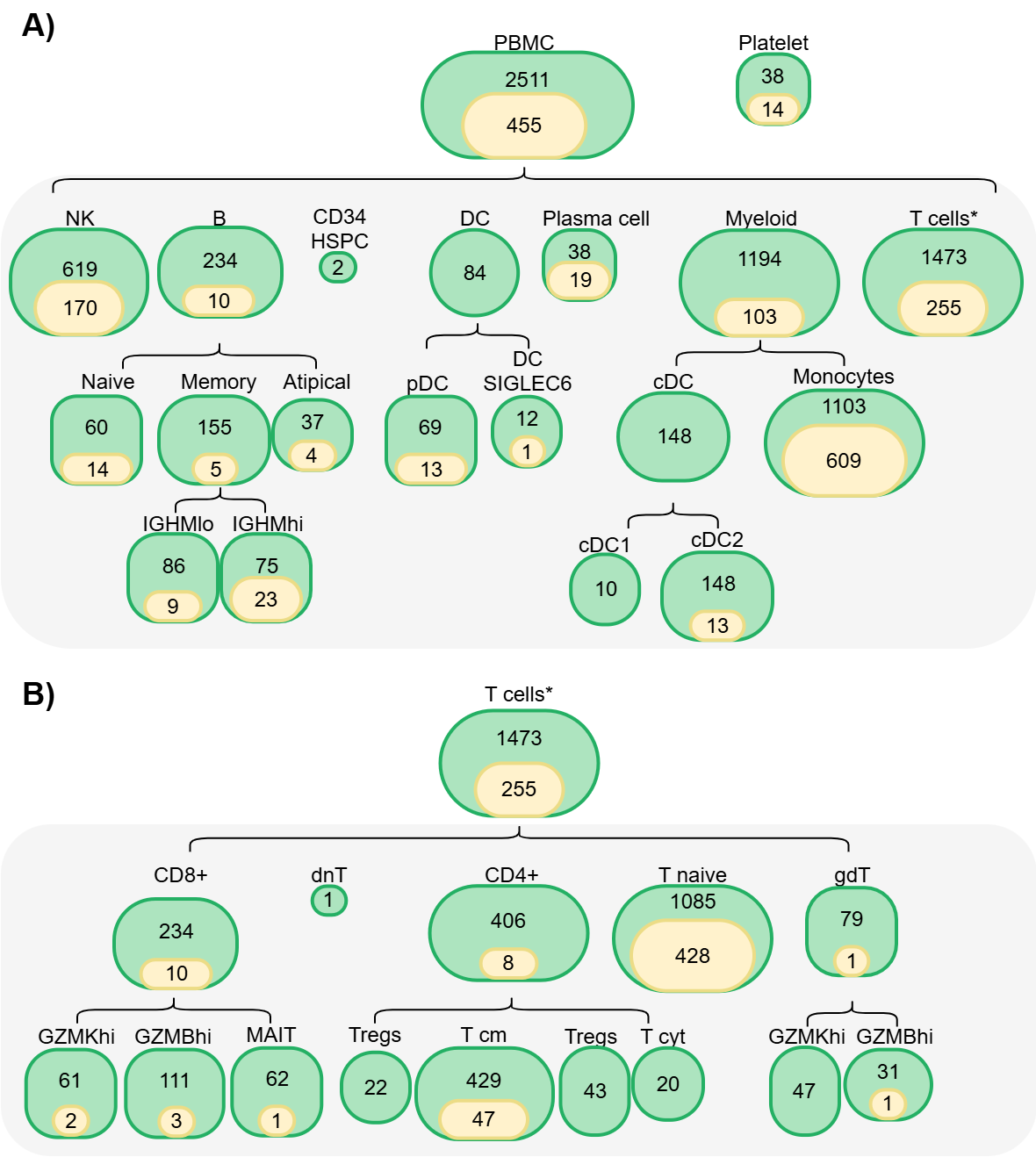

Supplement: giag042_Supplemental_Files [file giag042_supplemental_files.zip › fig_s2.png]

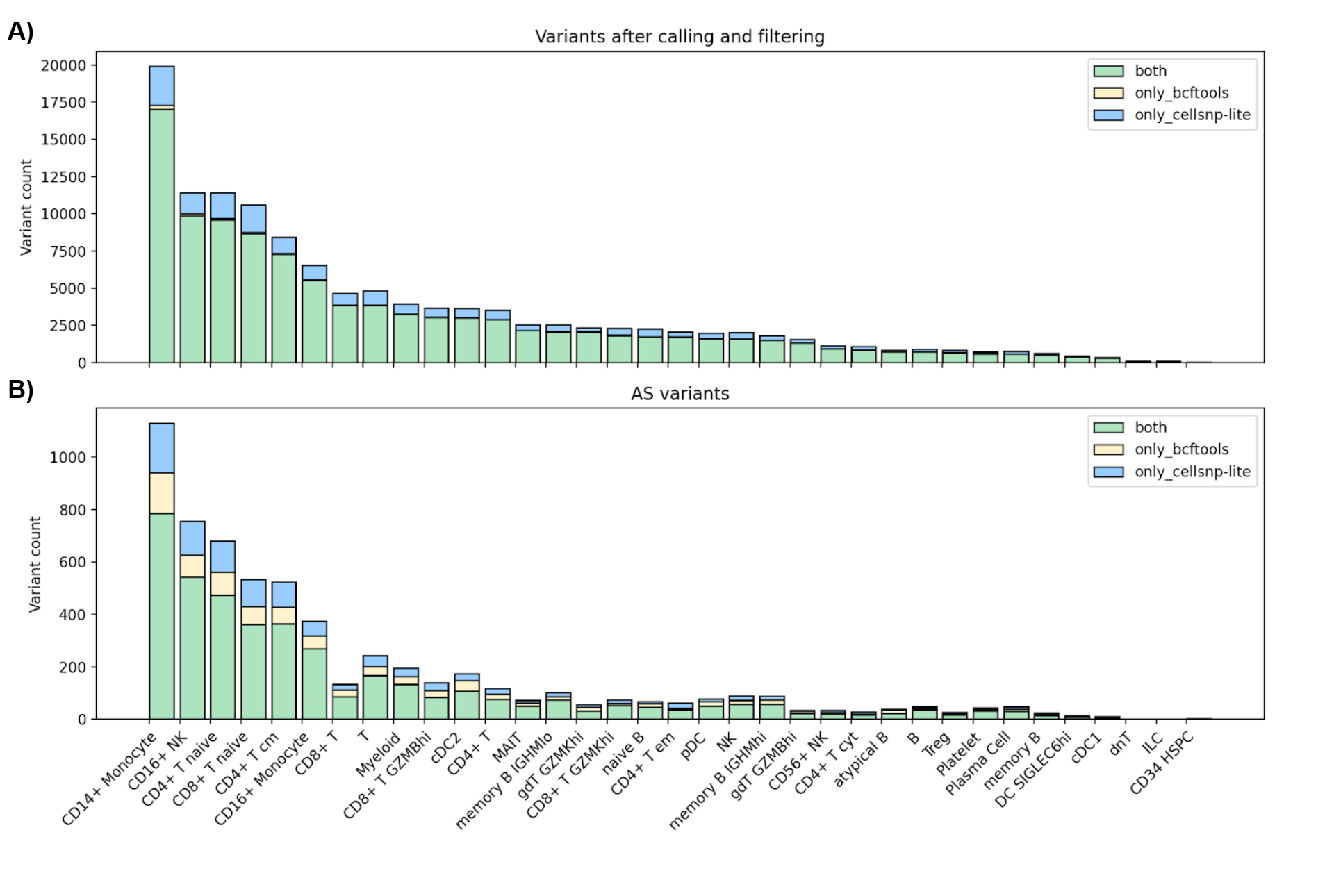

Supplement: giag042_Supplemental_Files [file giag042_supplemental_files.zip › s3_revision.png]

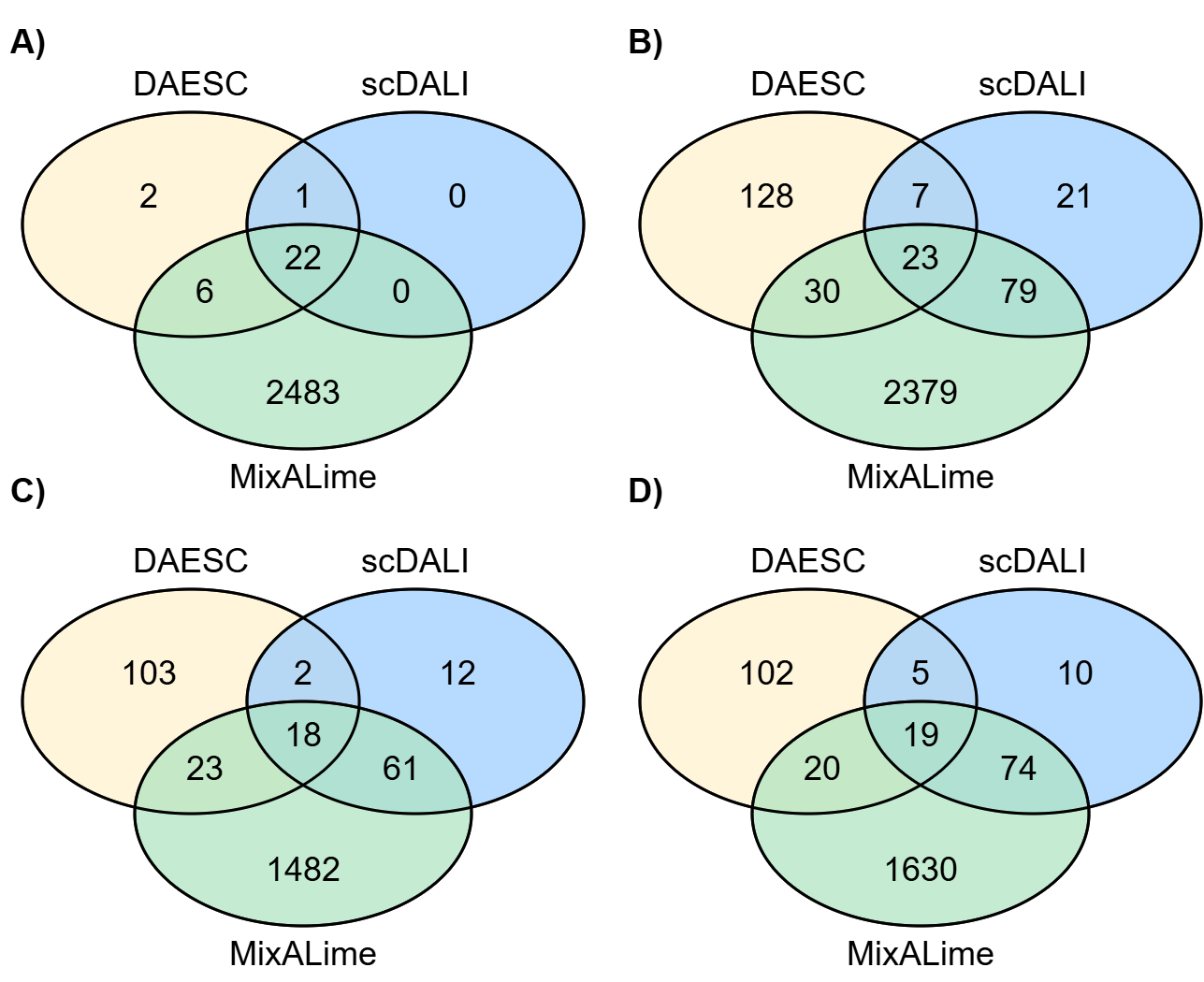

Supplement: giag042_Supplemental_Files [file giag042_supplemental_files.zip › s4_revision.png]

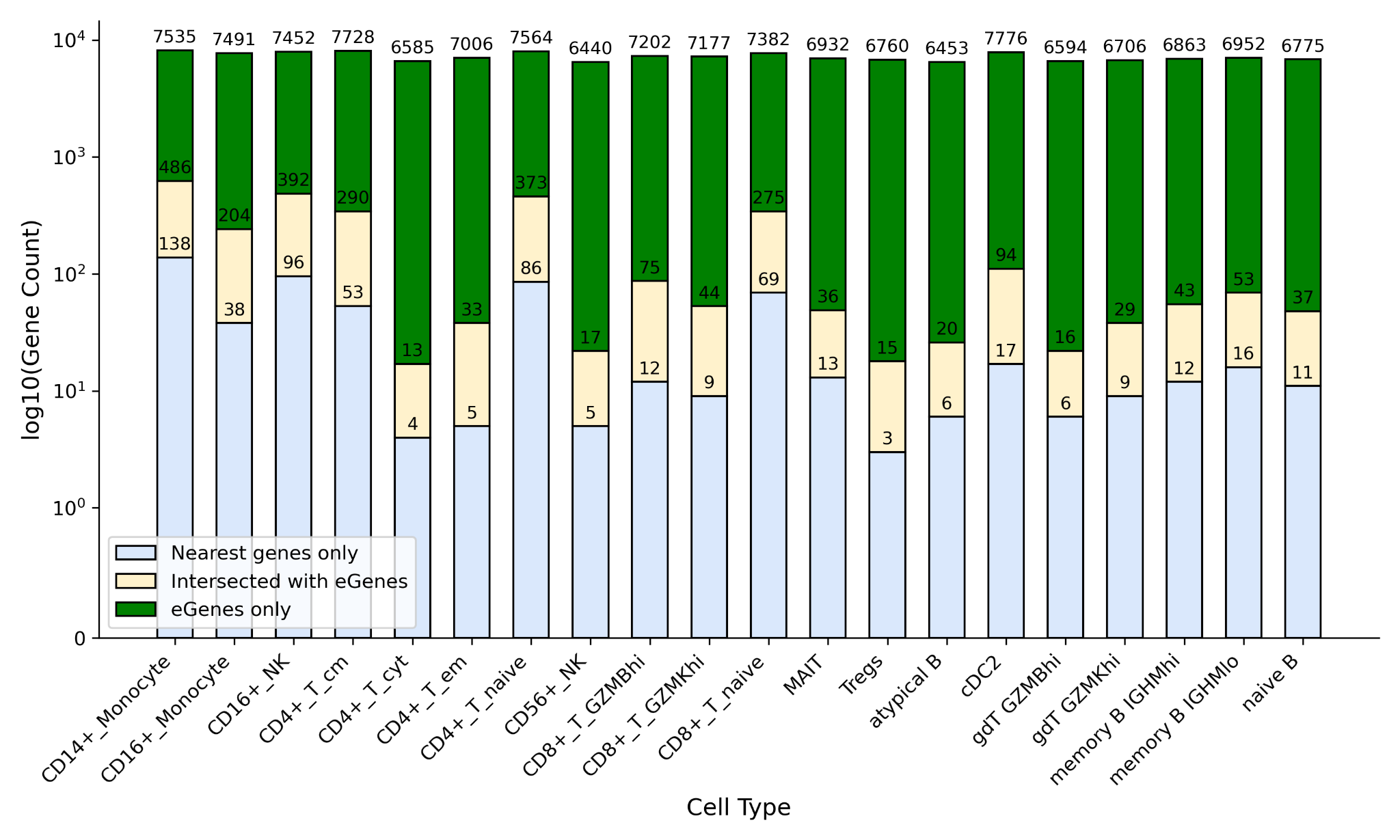

Supplement: giag042_Supplemental_Files [file giag042_supplemental_files.zip › s5_revision.png]
