# Supplementary material for: NEXT-scASV: a Nextflow pipeline for allele-specific variant calling from single-cell RNA-seq data
Source: Gigascience. 2026 Apr 6;15:giag042. doi: 10.1093/gigascience/giag042 (PMC13148397; doi:10.1093/gigascience/giag042)

GigaScience

NEXT-scASV: A Nextflow Pipeline for Allele-Specific Variants Calling from single cell RNA-seq data

--Manuscript Draft--

**Manuscript Number:**	GIGA-D-25-00386
**Full Title:**	NEXT-scASV: A Nextflow Pipeline for Allele-Specific Variants Calling from single cell RNA-seq data
**Article Type:**	Research
**Funding Information:**	RSF (23-14-00371) — Mrs. Yulia Medvedeva<br>MSHERF (075-15-2025-014) — Mr. Ivan Kulakovskiy<br>FFRW (FFRW-2025-010) — Mr. Ivan Kulakovskiy

Abstract:

Abstract

The rapid accumulation of single-cell sequencing data presents major computational challenges in reproducibility, scaling, and handling data imperfections like sparsity and technical variations, which complicate even basic analyses. The next level of complexity is the allele-specific analysis, focused on identifying differential gene expression or regulation between homologous chromosomes by estimating the allelic imbalance of read counts at individual single-nucleotide variants.

Here we present NEXT-scASV, a scalable Nextflow pipeline for calling allele-specific variants (ASVs) from 5′ single-cell RNA sequencing data. NEXT-scASV automates the entire process—from read alignment and quality control to variant calling and statistical evaluation of the allelic imbalance—within a containerized environment, ensuring reproducibility and ease of deployment across platforms. NEXT-scASV is able to perform de novo ASV detection from single-cell sequencing data without prior genotyping. Its modular design allows for massive parallelization, efficiently handling the scale of modern atlas-level studies.

We validate NEXT-scASV on a dataset of 135K peripheral blood mononuclear cells (PBMCs) from 57 donors, demonstrating that it processes large-scale data efficiently, completing analysis in a week on a cluster with one node and 100 threads. Crucially, the pipeline reliably identifies ASVs even in rare cell populations (e.g. gdT GZMBhi and memory B IGHMhi cells), which remains elusive for bulk analyses. We also successfully detect allele-specific regulation of long non-coding RNAs and other lowly expressed, cell type-specific genes. Genes linked to detected ASVs show a high concordance (80%) with previously reported eQTLs. This strong validation confirms that NEXT-scASV produces biologically relevant results, making it a powerful tool for uncovering allele-specific regulation in large-scale, complex single-cell studies.

**Corresponding Author:**	Andrey Vadimovich Shevtsov, M.D.<br>Federal Research Centre Fundamentals of Biotechnology: FGU Federal'nyj issledovatel'skij centr Fundamental'nye osnovy biotehnologii Rossijskoj akademii nauk<br>Moscow, RUSSIAN FEDERATION
**Corresponding Author Secondary Information:**	
**Corresponding Author's Institution:**	Federal Research Centre Fundamentals of Biotechnology: FGU Federal'nyj issledovatel'skij centr Fundamental'nye osnovy biotehnologii Rossijskoj akademii nauk
**Corresponding Author's Secondary Institution:**	
**First Author:**	Andrey Shevtsov
**First Author Secondary Information:**	
**Order of Authors:**	Andrey Shevtsov<br>Andrey Boyan

Powered by Editorial Manager® and ProduXion Manager® from Aries Systems Corporation

	Vladimir Nozdrin
	Pavel Akhtyamov
	Alexei Stupnikov
	Georgy Meshcheryakov
	Ivan Kulakovskiy
	Yulia Medvedeva
**Order of Authors Secondary Information:**	

**Additional Information:**	
**Question**	**Response**
Are you submitting this manuscript to a special series or article collection?	No
**Experimental design and statistics**<br><br>Full details of the experimental design and statistical methods used should be given in the Methods section, as detailed in our Minimum Standards Reporting Checklist. Information essential to interpreting the data presented should be made available in the figure legends.<br><br>Have you included all the information requested in your manuscript?	Yes
**Resources**<br><br>A description of all resources used, including antibodies, cell lines, animals and software tools, with enough information to allow them to be uniquely identified, should be included in the Methods section. Authors are strongly encouraged to cite Research Resource Identifiers (RRIDs) for antibodies, model organisms and tools, where possible.<br><br>Have you included the information requested as detailed in our Minimum Standards Reporting Checklist?	Yes
**Availability of data and materials**<br><br>All datasets and code on which the	Yes

Powered by Editorial Manager® and ProduXion Manager® from Aries Systems Corporation

conclusions of the paper rely must be either included in your submission or deposited in publicly available repositories (where available and ethically appropriate), referencing such data using a unique identifier in the references and in the "Availability of Data and Materials" section of your manuscript.<br><br>Have you have met the above requirement as detailed in our Minimum Standards Reporting Checklist?	
GigaScience has policies and guidelines in place for the use of generative AI-writing tools such as ChatGPT. If you have used such writing tools to assist with writing the manuscript this must be declared and cited in the text. Authors should not list AI-writing tools and other AI-assisted technologies as an author or co-author and should acknowledge that they are fully responsible for text generated or refined by AI-writing tools.<p><br><br>A summary of use (particularly in the introduction or among methods) needs to be included at the end of the paper, and the outputs should also be included as a supplementary file hosted in GigaDB or other open repositories. Please read our guidelines for more information. <p><br><br>By submitting to GigaScience, you are aware of the journal's AI-writing tools policy, and if you have declared use of such tools below, you have acknowledged this where appropriate in your manuscript and have made a summary of use and outputs available. <p><br><b>AI-assisted writing tools have been used in the preparation of this manuscript?	No

Powered by Editorial Manager® and ProduXion Manager® from Aries Systems Corporation

Manuscript Click here to access/download;Manuscript;next-scasv_main.docx

Title

NEXT-scASV: A Nextflow Pipeline for Allele-Specific Variants Calling from single cell RNA-seq data.

Authors

Andrey Shevtsov [1,2], Andrey Buyan [3], Vladimir Nozdrin [4], Pavel Akhtyamov [5, 6], Alexei Stupnikov [1, 7], Georgy Meshcheryakov [3], Ivan V. Kulakovskiy [3, 7, 8] & Yulia A. Medvedeva [1]

1- Research Institute of Biotechnology, Russian Academy of Sciences
2- Bioinformatics Group, AIRI, Moscow 121170, Russia
3- Institute of Protein Research, Russian Academy of Sciences, Pushchino, Russia
4- Faculty of Bioengineering and Bioinformatics, Lomonosov Moscow State University, Moscow, Russia
5- Moscow Center for Advanced Studies, Department of Biomedical Physics, Moscow, 123592, Russia
6- National Research University Higher School of Economics, 11 Pokrovksy Bulvar, Moscow, 109028, Russia
7- Vavilov Institute of General Genetics, Russian Academy of Sciences, Moscow, Russia
8- Institute of Biochemistry and Genetics, Ufa Federal Research Centre of the Russian Academy of Sciences, Ufa, Russia

ABSTRACT

The rapid accumulation of single-cell sequencing data presents major computational challenges in reproducibility, scaling, and handling data imperfections like sparsity and technical variations, which complicate even basic analyses. The next level of complexity is the allele-specific analysis, focused on identifying differential gene expression or regulation between homologous chromosomes by estimating the allelic imbalance of read counts at individual single-nucleotide variants.

Here we present NEXT-scASV, a scalable Nextflow pipeline for calling allele-specific variants (ASVs) from 5′ single-cell RNA sequencing data. NEXT-scASV automates the entire process—from read alignment and quality control to variant calling and statistical evaluation of the allelic imbalance—within a containerized environment, ensuring reproducibility and ease of deployment across platforms. NEXT-scASV is able to perform *de novo* ASV detection from single-cell sequencing data without prior genotyping. Its modular design allows for massive parallelization, efficiently handling the scale of modern atlas-level studies.

We validate NEXT-scASV on a dataset of 135,000 peripheral blood mononuclear cells (PBMCs) from 57 donors, demonstrating that it processes large-scale data efficiently, completing analysis in a week on a cluster with one node and 100 threads. Crucially, the pipeline reliably identifies ASVs even in rare cell populations (e.g. gdT GZMBhi and memory B IGHMhi cells), which remains elusive for bulk analyses. We also successfully detect allele-specific regulation of long non-coding RNAs and other lowly expressed, cell type-specific genes. Genes linked to detected ASVs show a high concordance (80%) with previously reported eQTLs. This strong validation confirms that NEXT-scASV produces biologically relevant results, making it a powerful tool for uncovering allele-specific regulation in large-scale, complex single-cell studies.

Availability: https://github.com/MedvedevaLab/NEXT-scASV

KEYWORDS: allele-specific variants, single-cell RNA-seq, Nextflow, computational pipeline, regulatory variants

BACKGROUND

Genetic variation within regulatory elements represents a fundamental mechanism of phenotypic diversity and disease susceptibility (Ge et al. 2009; Abramov et al. 2021). In diploid genomes, particular regulatory variants can be identified from comparison of allelic signals at homologous chromosomes, revealing allele-specific gene expression (ASE), transcription factor binding (ASB), and chromatin accessibility (ASA), representing different types of allele-specific variants (ASVs).

Allele-specific variants have been widely studied using bulk sequencing (Maurano et al. 2015; de Santiago et al. 2017; Chen et al. 2016; Miller et al. 2021; Amoah et al. 2021), which yields averaged signals across often heterogeneous cell populations. The averaging obscures cell type-specific effects, potentially diluting the signal from biologically meaningful, context-dependent regulation (Liu, Dong, and Li 2018; Frésard et al. 2019). The advent of single-cell sequencing (including scRNA-seq) provides the resolution necessary to interrogate ASVs and ASE within defined cell types (Cummings et al. 2017; Stachowiak, Szczerbal, and Flisikowski 2018).

5′ scRNA-seq offers a distinct advantage for allele-specific analysis. By capturing the 5′ ends of transcripts, scRNA-Seq reads are strongly enriched in regulatory regions in the close vicinity to the transcription start sites (TSSs), such as promoters and transcribed enhancers, physically linking genetic variants to the expression of their target genes on the level of individual reads (Y. Li et al. 2023). This allows for direct and unambiguous assignment of regulatory effects compared to 3′ scRNA-seq protocols, which require imputation or statistical phasing. Furthermore, the high sensitivity afforded by cell type-specific analysis and the direct capture of 5' ends opens the possibility of investigating allele-specific regulation in challenging genomic contexts. These include long non-coding RNAs (lncRNAs) and other lowly expressed or highly tissue-specific genes, which are often

underrepresented in bulk tissue eQTL studies due to signal dilution across heterogeneous cell populations. Notably, the promoters of lncRNAs often contain highly specific genetic features (Alam et al. 2014) that are susceptible to disruption by genetic variants. This underscores the unique advantage of 5' scRNA-seq, and by extension our pipeline, for directly interrogating the allele-specific mechanisms governing lncRNA expression.

However, analyzing scRNA-seq data for ASE is a multi-faceted computational challenge, and very few existing tools allow for this kind of analysis (Mattevi, Mazzarotto, and Martini 2025). The process involves numerous steps: demultiplexing, quality control, read alignment and deduplication, variant calling, allelic read counting, and finally, statistical modeling to pinpoint the single-nucleotide variants with the significant allelic imbalance. Each step requires specific software tools with complex dependencies, orchestrating such a workflow with custom scripts is fragile, difficult to parallelize, and notoriously hard to reproduce, creating a significant barrier to reliable discovery. Workflow management systems such as Nextflow (Di Tommaso et al. 2017) have emerged to solve these problems and gained popularity. Nextflow enables the creation of portable, scalable, and reproducible pipelines by abstracting away complex job scheduling, managing software dependencies through containers (Docker (Ratliff 2025), Singularity (Kurtzer, Sochat, and Bauer 2017)), and providing built-in mechanisms for logging and resuming failed runs.

Here, we present NEXT-scASV (Nextflow pipeline for Allele-Specific Variant calling), a comprehensive and robust workflow designed specifically for discovery of allele-specific variants from 5′ scRNA-seq data. NEXT-scASV integrates established tools for alignment (HISAT2 (Kim et al. 2019)), reads deduplication (umi_tools (Smith, Heger, and Sudbery 2017)), variant calling (bcftools mpileup (H. Li et al. 2009)), reference bias correction (WASP (van de Geijn et al. 2015)), and advanced statistical modeling (MIXALIME (Buyan et al. 2025)). We demonstrate its performance on a large dataset of human PBMCs. The high sensitivity afforded by cell type-specific analysis and the direct capture of 5' ends opens the possibility of investigating allele-specific regulation in rare cell populations or challenging genomic contexts, such as in long non-coding RNAs (lncRNAs) and other lowly expressed or highly tissue-specific genes, which are often underpowered in bulk tissue eQTL studies. By providing a standardized, containerized, and parallelized solution, NEXT-scASV lowers the barrier for performing sophisticated allele-specific analyses in large-scale single-cell studies.

MATERIALS AND METHODS

Pipeline Design and Implementation

NEXT-scASV is implemented in Nextflow (DSL2) with a modular structure where each processing stage (or subflow) is defined as a separate, reusable module. The pipeline execution is managed through a central configuration file, defining input data paths, sample metadata, and important parameters for each module. All software dependencies are packaged into Docker, supporting reproducibility across different computing environments.

The pipeline requires two primary inputs from the user:

1. A metadata JSON file linking sample identifiers to the paths of the corresponding FASTQ files.
2. A barcode-to-group assignment CSV file containing columns for *barcode*, *sample*, and *group* (e.g., a cell type). This file is essential for splitting the data and performing group-level analyses.

The workflow consists of five main subflows, illustrated in Figure 1: (i) Data Splitting, (ii) Alignment and Filtering, (iii) Variant Calling, (iv) Allelic Read Counting, and (v) ASV Calling.

Splitting Subflow

The initial step employs a custom Python script to split the raw FASTQ files based on the provided barcode-group assignments. Each input sample (e.g., one donor) is split into multiple FASTQ files, one for each assigned group (e.g., a cell type). This strategy, while increasing the number of subsequent tasks, is the cornerstone of the pipeline's parallelization, allowing downstream steps to process each sample × group combination independently and in parallel.

Aligningment Subflow

Each split FASTQ file is then processed individually. First, the reads are trimmed to remove adapters and low-quality bases using cutadapt v5 (Martin 2011). Processed reads are then aligned to the reference genome (e.g., GRCh38) using HISAT2, which efficiently handles splice-aware alignment of RNA-seq data. Following alignment, PCR duplicates are marked and removed using umi_tools dedup (Smith, Heger, and Sudbery 2017) to mitigate technical artifacts. The resulting BAM files are rigorously filtered to retain only high-quality alignments, for our testing we set the following values for the user defining parameters: mapping quality (MAPQ) ≥ 10, a maximum of 2 mismatches, and an insert size ≤ 750 bp.

Variant Calling Subflow

To call heterozygous single-nucleotide variants (SNVs), as in the default MIXALIME workflow (Buyan et al. 2025), the pipeline processes the filtered BAM files from each sample × group combination using bcftools mpileup and bcftools call (H. Li et al. 2009). Variant calling is performed independently for each chromosome (1-22) to maximize parallelization. The initial call set is stringently filtered to ensure high-confidence heterozygous sites: read depth (DP) ≥ 10, genotype quality (GQ) ≥ 50, and a minimum of 5 reads supporting each allele (AD ≥ 5). At the vcf splitting step, jointly called variants are splitted by samples and each sample vcf file is also filtered by minimum 5 reads supporting each allele.

Allelic Read Counting Subflow

A critical challenge in ASV analysis is reference allele mapping bias, where reads containing alternative alleles map with lower confidence. To correct this, the pipeline integrates the WASP tool (van de Geijn et al. 2015). For each potential variant site, WASP identifies reads overlapping the site, swaps the alleles, remaps these modified reads, and filters out the original reads that fail to remap correctly. This process generates a corrected set of alignments devoid of reference bias. A Python script (Vierstra et al. 2020) then parses

these corrected BAM files to count the number of reads supporting the reference and alternative alleles for every variant in every sample × group combination, producing an extensive table of allelic read counts for statistical testing.

ASV Calling Subflow

The final step identifies the sites with the significant allelic imbalance using the MIXALIME framework (Buyan et al. 2025). MIXALIME fits probabilistic models to the allelic read counts, accounting for data sparsity and over-dispersion inherent in scRNA-seq. We employed the Beta-Negative Binomial (BetaNB) model, which is the most conservative but reliable for modeling over-dispersed count data. The tool fits model parameters and calculates P-values for the deviation from the expected balanced allelic read counts (i.e., 0.5 ratio). P-values are then aggregated with the Mudholkar-George *logitp* method (George and Mudholkar 1983) across hierarchical groups (e.g., within a cell type lineage) to increase statistical power. False discovery rate (FDR) correction is applied (Benjamini-Hochberg), and the significant ASVs are called at, FDR < 0.05. MIXALIME also generates extensive quality control plots, including goodness-of-fit metrics like RMSEA (Root Mean Square Error of Approximation) (Browne and Cudeck 1992). Significant ASVs are functionally annotated by overlapping their genomic coordinates with regulatory databases such as GTEx for eQTLs and ADASTRA for allele-specific binding events (Aguet et al. 2020; Abramov et al. 2021), providing immediate biological context and an additional layer of validation against known regulatory single-nucleotide polymorphisms (rSNPs).

Benchmarking Dataset

The pipeline was tested on data from the Asian Immune Diversity Atlas (AIDA) (Kock et al. 2025), a comprehensive resource of scRNA-seq data from PBMCs of 619 healthy donors. A random subset of 57 donors (23 male, 34 female, aged 25-40) of South Korean ancestry was selected for this benchmarking study to balance computational feasibility with statistical power. The dataset comprised 17 major immune cell types, as annotated in the original paper.

Computational Resource Profiling

The pipeline was executed on a high-performance computing cluster managed by the SLURM workload manager, with a constrained limit of 1 node with 100 threads and 15 TB of RAM. Resource usage (CPU time, peak memory, disk I/O) for each process was meticulously recorded using Nextflow's built-in tracing capabilities. The total execution time was calculated under three scenarios: the sum of all process times (emulating a single CPU, lower bound), the duration of the longest process chain (emulating infinite parallelization, upper bound), and the actual wall time under the 100 threads constraint.

Biological Validation against eQTL Data

To assess the biological relevance of the discovered ASVs, we compared our results against the eQTL analysis published in the original AIDA study. For each significant ASV, we identified its nearest transcription start site (TSS). The resulting list of ASV-associated genes

was intersected with the list of genes identified as significantly associated with eQTLs (eGenes) in the AIDA cohort (FDR < 0.05).

RESULTS

NEXT-scASV Pipeline Enables Scalable and Reproducible Analysis

The NEXT-scASV pipeline integrates a complex series of tools into a cohesive, automated workflow (Figure 1). Its modular Nextflow DSL2 implementation allows users to easily start, stop, and resume analyses, a critical feature for long-running computations. The use of containers eliminates "dependency hell" and ensures identical results regardless of the host system.

A key design feature is the aggressive data splitting at the outset. While this strategy generates a large number of intermediate files (~15 TB for this study), it unlocks massive parallelization, transforming a computationally prohibitive task into a manageable one. The configuration of the pipeline allows fine-grained control over resource allocation for each step and provides options for cleaning up intermediate files to conserve disk space after successful completion of individual stages.

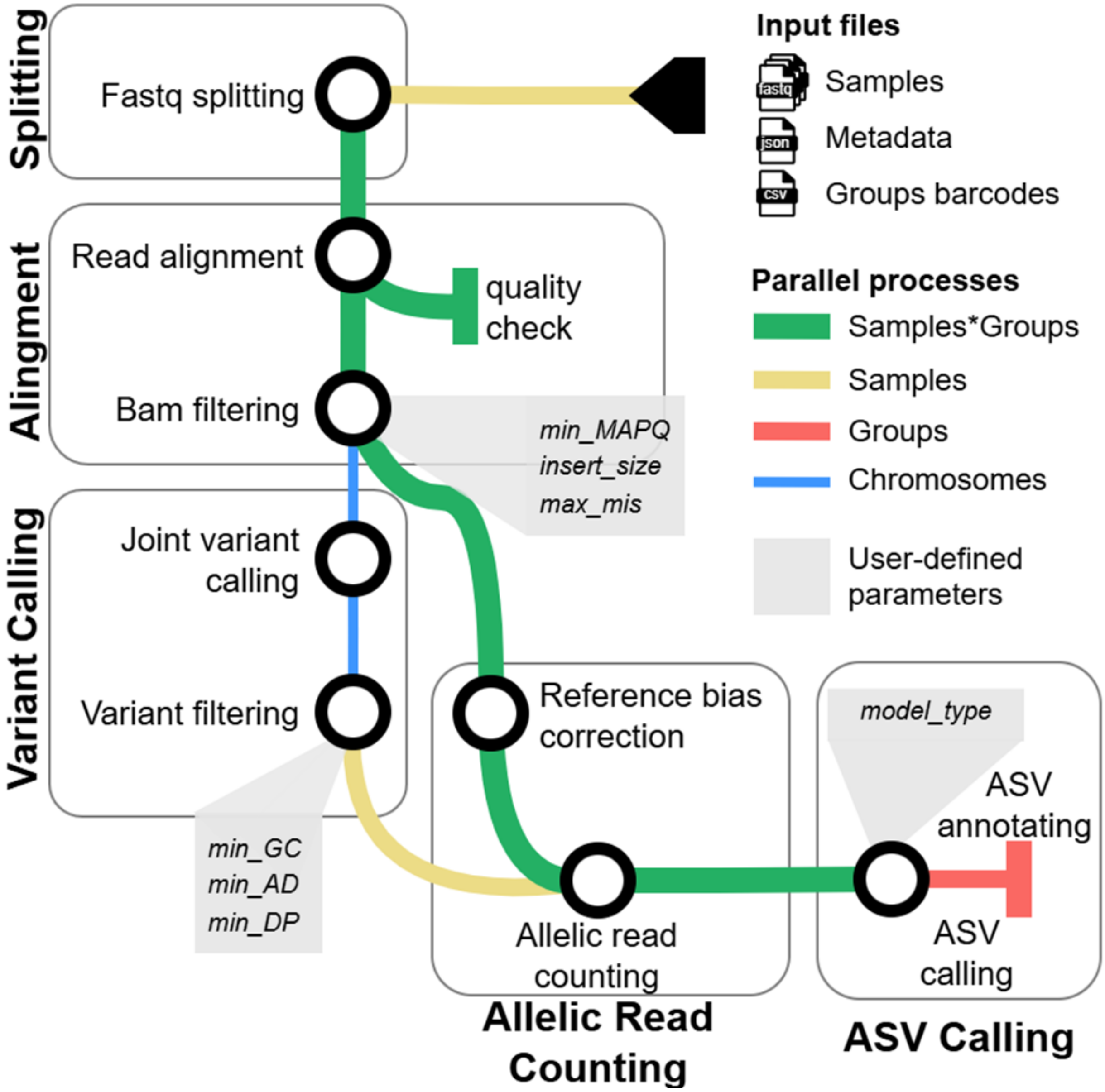

Figure 1. Schematic overview of the NEXT-scASV pipeline. Rounded rectangles represent the five main subflows. Circles represent individual modules within a subflow. The color and thickness of the arrows indicate the data aggregation strategy. Grayscale rectangles highlight important user-configurable parameters.

Pipeline Performance and Resource Utilization

We evaluated the computational cost of processing the 57-donor PBMC dataset. Under a realistic constraint of 100 threads, the pipeline completed in approximately 7 days (Figure 2B). Profiling the resource usage revealed distinct patterns for different stages (Figure 3). The alignment stages ("fastq aligning" and "reference bias correction") were the

most time-consuming but scaled efficiently with the number of available CPUs. In contrast, the "Call variants" stage, split into 22 parallel chromosome-level tasks, represents a parallelization bottleneck; its duration is fixed by the longest chromosome job and cannot be reduced by adding more CPUs beyond this point. This stage also exhibited the highest memory demand, with a peak virtual memory usage of ~120 GB per process and intensive disk I/O (reading/writing over 140 GB per process), highlighting the need for substantial memory and fast storage on compute nodes.

Theoretical estimates illustrate the necessity of parallelization: running the workflow sequentially on a single CPU would take over 400 days (Figure 2C), while perfect parallelization could reduce this to under a day (Figure 2A). Our real-world scenario (100 CPUs) strikes a practical balance, demonstrating that NEXT-scASV can process atlas-scale data within a reasonable timeframe on a high-performance computing cluster typically used in data-intensive biomedical research.

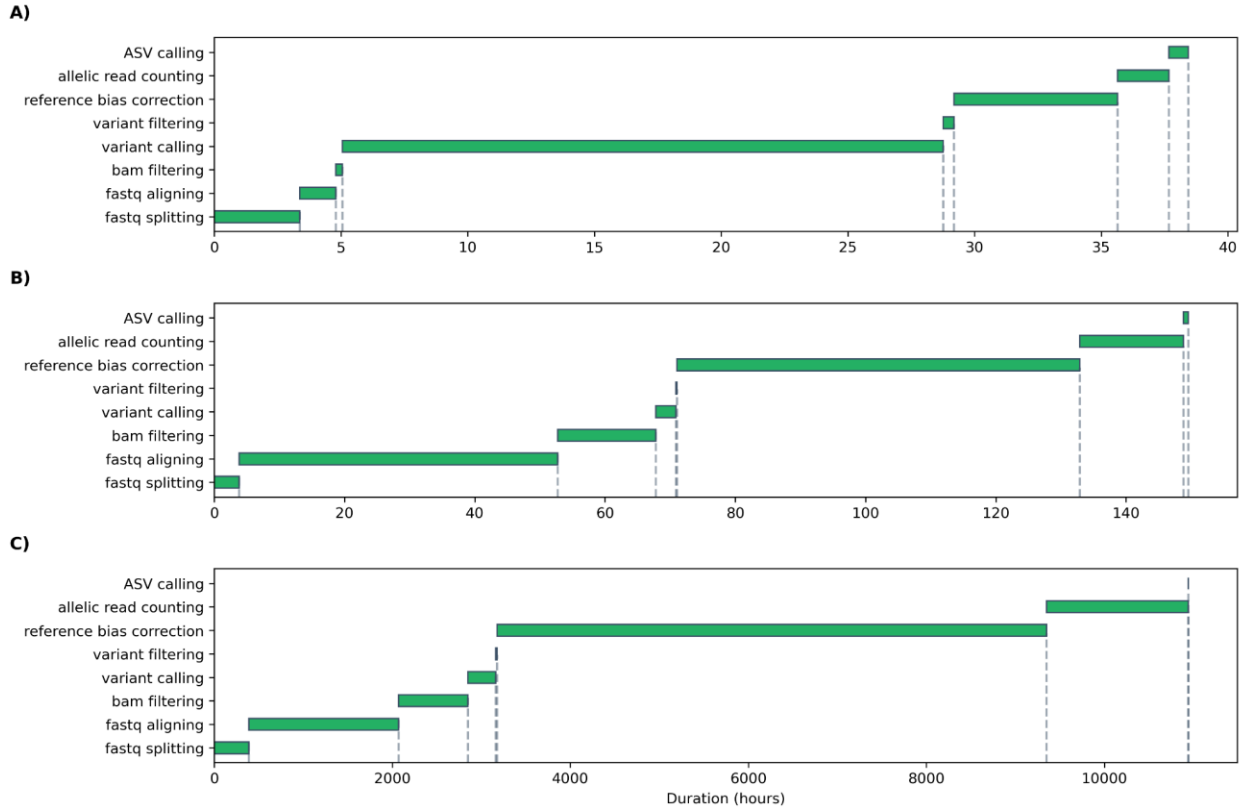

Figure 2. Pipeline execution time under different parallelization scenarios. (A) Mean process duration per stage, representing the ideal upper bound with infinite parallelization. (B) Actual wall-time duration with a limit of 100 concurrent CPUs. (C) Cumulative sum of all process times, representing the lower bound of sequential execution on a single CPU.

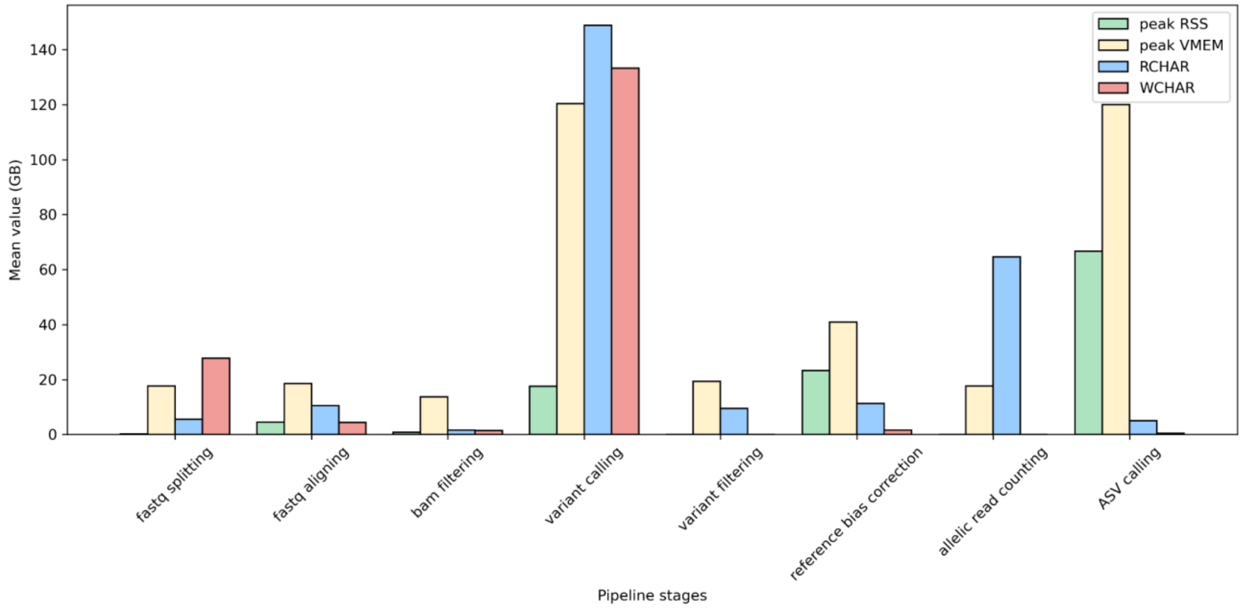

Figure 3: Mean computational resource utilization per process for each major pipeline stage. Metrics shown include: peak RSS (peak Resident Set Size , the maximum amount of physical memory a process has used at any point during its execution), peak VMEM (peak virtual memory usage, includes swap), RCHAR (volume of data read from storage), and WCHAR (volume of data written to storage).

Quality Control and Model Validation

The statistical analysis using MIXALIME passed the basic quality check. Particularly, the goodness-of-fit, measured by RMSEA for all fitted models, was excellent, falling well below the recommended threshold of 0.05 (Supplementary Figure S1A), indicating that the chosen BetaNB model adequately captured the overall distribution of allelic read counts.

As an orthogonal validation, we examined the fraction of discovered ASVs annotated in external databases of rSNPs (GTEx eQTLs and ADASTRA ASBs). We observed that the proportion of annotated variants increased monotonically along with higher thresholds for the FDR significance (Supplementary Figure S1B). This trend is biologically expected—stronger, more reproducible functional variants are more likely to be catalogued in existing databases—and provides independent support for the correctness of our statistical calls.

NEXT-scASV Uncovers Allele-Specific Events in Rare Cell Populations and Challenging Genomic Conditions

A major advantage of single-cell resolution is the ability to probe allele-specific regulation in rare cell types. We leveraged MIXALIME's ability to aggregate p-values across different hierarchical levels (e.g., within T-cell subsets before aggregating to all T cells) to enhance statistical power at different levels of resolution. As expected, aggregating data to

broader cell types (e.g., all T cells, all PBMCs) increased the total number of detectable ASVs due to greater statistical power from larger read counts (Supplementary Figure S2).

The sensitivity gains from analyzing particular cell types extend beyond discovering effects in rare populations to also detecting ASE of genes with low or cell type-specific expression. This includes challenging targets, such as long non-coding RNAs (lncRNAs), which are often expressed at low levels and whose regulation is highly context-dependent. In our case study, we identified significant ASVs impacting the expression of several non-coding RNAs. Fractions of the detected ASV impacting non-coding RNAs are the same as for all genes (Figure 3B). Importantly, we found in total 19 long non-coding RNAs, some of them are well-studied regulators such as P53 Induced Transcript LINC-PINK or NLRP3 inflammasome regulator LINC00989, and also a number of less-studied RNAs (e.g., LINC02723, LINC01220, LINC01871, LINC01679, LINC02273, LINC01619), that may be included in immune cells regulation. The ability to probe allele-specific regulation of such genes provides a new avenue for understanding the functional impact of genetic variation in the non-coding genome.

High Concordance with Independent eQTL Analysis Validates Biological Relevance

To validate the biological significance of our findings, we compared the set of genes linked to the detected ASVs with those reported as significantly associated with eQTLs (eGenes) in the AIDA cohort study (Kock et al. 2025). This comparison showed a very high level of agreement: on average, 82% (± 6% across cell types) of the genes associated with ASVs were also identified as eGenes (Figure 4). This strong concordance with an orthogonal analysis performed on a larger cohort with genotyping data provides robust evidence that NEXT-scASV reliably identifies biologically genuine cis-regulatory variants.

The remaining ~18% of ASV-linked genes not reported as eGenes likely represent a mix of false positives and, more interestingly, true cell type-specific regulatory events. These could either be effects that are too weak, narrow context-specific, or dependent on cellular environments not captured in the bulk-tissue eQTL analysis, pointing to potential novel biology uncovered by our single-cell approach.

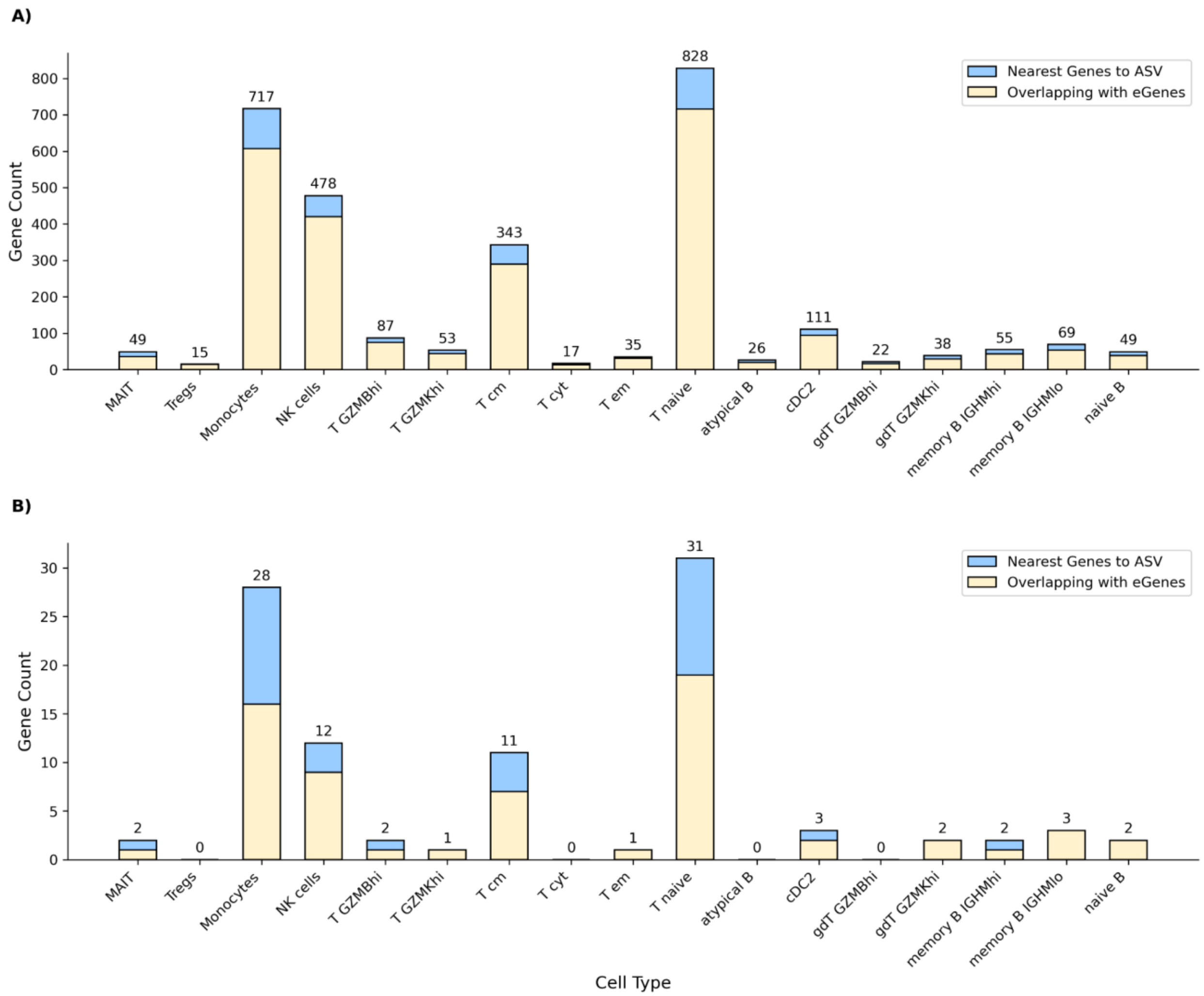

Figure 4. Validation of discovered ASVs by comparison against eQTLs analysis. For each cell type, the bar shows the total number of genes associated with significant ASVs (blue). The yellow segment indicates the subset of these genes that were also reported as eGenes (FDR < 0.05) in the original cohort eQTL study. A. All genes, B. Non-coding RNAs.

DISCUSSION

We have developed NEXT-scASV, a scalable and reproducible pipeline for the detection of allele-specific variants from 5′ scRNA-seq data. Its containerized implementation ensures that the ASV calling is reproducible across different computing platforms, a cornerstone of robust scientific practice. Our benchmarking on data from 57 individuals (135,000 cells) demonstrates that NEXT-scASV is capable of processing large datasets in a feasible timeframe on a mid-sized compute cluster. The resource analysis provides a valuable guide for other groups planning similar studies. The pipeline performance is not just technical: its results agree well with existing rSNP annotations

showing 80% concordance with established eQTLs, while also revealing cell type-specific effects that are invisible to bulk approaches.

A key design philosophy was to enable *de novo* discovery without requiring external genotyping data, making the pipeline widely applicable to standard 5' scRNA-seq study designs. The integration of WASP mitigates reference mapping bias, a known source of false positives in ASE studies, and the use of MIXALIME provides a statistically rigorous framework capable of handling the overdispersion in allelic counts arising due to noise and sparsity of single-cell data.

The ability of NEXT-scASV to detect signals in rare cell types like gdT GZMBhi cells is particularly exciting. It opens the door to investigating allele-specific regulation in rare cell populations involved in disease, development, and immune response, which have been largely inaccessible. An exciting implication of our approach is its potential to illuminate allele-specific regulation in genomic regions that have been historically difficult to study. The combination of cell type-specific resolution and sensitivity makes NEXT-scASV particularly suited to investigate ASE of lncRNAs and other lowly expressed genes. These categories are often poorly tagged in bulk tissue eQTL studies due to their low expression and high cell type specificity. By isolating the relevant cell type, our pipeline can overcome this limitation, offering a powerful strategy to ascribe function to genetic variants associated with lncRNAs and other elusive elements of the regulome.

The modular, DSL2-based architecture of Nextflow is a core strength of NEXT-scASV, ensuring it is not a static tool but a flexible framework for future methodological developments. The pipeline can be readily adapted to other single-cell modalities that probe cis-regulatory activity, such as scATAC-seq for identifying allele-specific accessibility or multiome assays that simultaneously measure chromatin accessibility and gene expression. This would primarily involve swapping the alignment and initial processing modules while leveraging the same robust downstream variant calling and statistical analysis framework. Furthermore, the modular design simplifies the process of incorporating new best practices, such as advanced filters for technical artifacts or novel statistical models for allelic imbalance, ensuring the pipeline remains at the forefront of the field without requiring a complete rebuild. This adaptability makes NEXT-scASV a lasting resource for the community, capable of evolving alongside rapidly advancing single-cell technologies.

While NEXT-scASV provides a robust framework for allele-specific analysis, several limitations should be mentioned. First, the pipeline's current implementation requires a predefined cell type annotation provided by the user via a barcode-group assignment file. It is therefore dependent on the accuracy and resolution of this external annotation. While the hierarchical aggregation in MIXALIME mitigates this to some degree, integrating the pipeline with a more advanced probabilistic cell typing could be a valuable future direction.

Second, the computational footprint, particularly the storage requirements for intermediate files (~15 TB for this study) and the high I/O load during the variant calling stage, can be prohibitive for extra large studies without access to high-performance computing infrastructure with fast and capacious storage.

Third, the variant calling step relies solely on the scRNA-seq data itself. While this de novo approach is a key feature that increases broad applicability, it may be less sensitive than methods that incorporate external genotype information from array data or whole-genome sequencing. Finally, the statistical power to detect ASVs is inherently constrained by the number of expressed reads covering a heterozygous SNP in a given cell type. While aggregation of related cell types helps, very rare cell types or extremely lowly expressed genes will remain challenging to analyze, a limitation inherent to all current scRNA-seq analyses.

In conclusion, NEXT-scASV provides the community with a validated, scalable, and reproducible solution for deciphering allele-specific regulation from 5′ scRNA-seq data. By transforming a complex, multi-stage analysis into an automated and portable workflow, it empowers researchers to move beyond logistical challenges and focus on biological discovery. We demonstrate that it reliably recovers known regulatory biology while uniquely enabling the exploration of genetic effects in rare cell populations and understudied genomic elements. As single-cell atlas projects continue to expand, NEXT-scASV stands as a critical tool for unlocking the functional impact of genetic variation across the full spectrum of cellular diversity in health and disease.

AVAILABILITY OF DATA AND MATERIALS

The code and instructions for NEXT-scASV is available at https://github.com/MedvedevaLab/NEXT-scASV

AUTHOR CONTRIBUTIONS

Conceptualization: I.K., Y.M., A.Sh. Code curation: A.Sh., A.B., V.N., G.M. Data curation and analysis: A.Sh., P.A. Supervision: I.K., Y.M. Writing—original draft: A.Sh., Y.M. Writing—review and editing: A.Sh., Y.M., I.K., A.B., A.St., P.A.

FUNDING

Pipeline creation, testing, application to real data and analysis of the results has been supported by the RSF 23-14-00371 grant to Y.A.M. MIXALIME adaptation was supported by MSHERF grant number № 075-15-2025-014 (previously № 075-15-2024-666). ADASTRA ASB analysis was supported by assignment FFRW-2025-010.

COMPETING INTERESTS

We declare that we do not have any competing interests.

SUPPLEMENTARY DATA

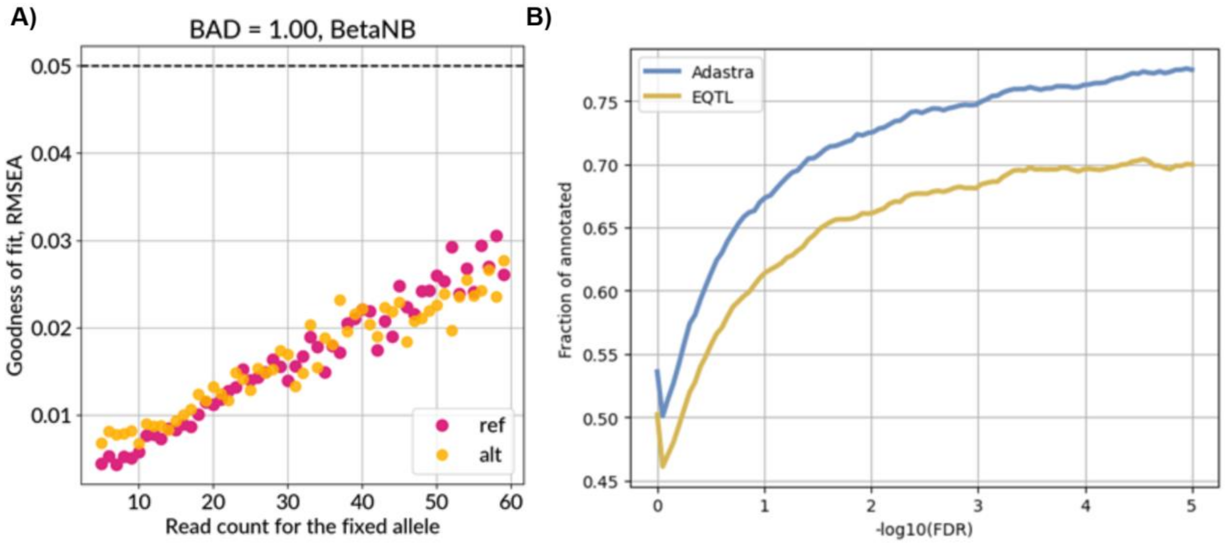

Figure S1: Quality control of the ASV detection. (A) Root Mean Square Error of Approximation (RMSEA) for all fitted MIXALIME models. The dashed line indicates the desired threshold of 0.05. (B) Fraction of significant ASVs that can be annotated in the GTEx (eQTL) and ADASTRA (ASB) databases as a function of the FDR threshold.

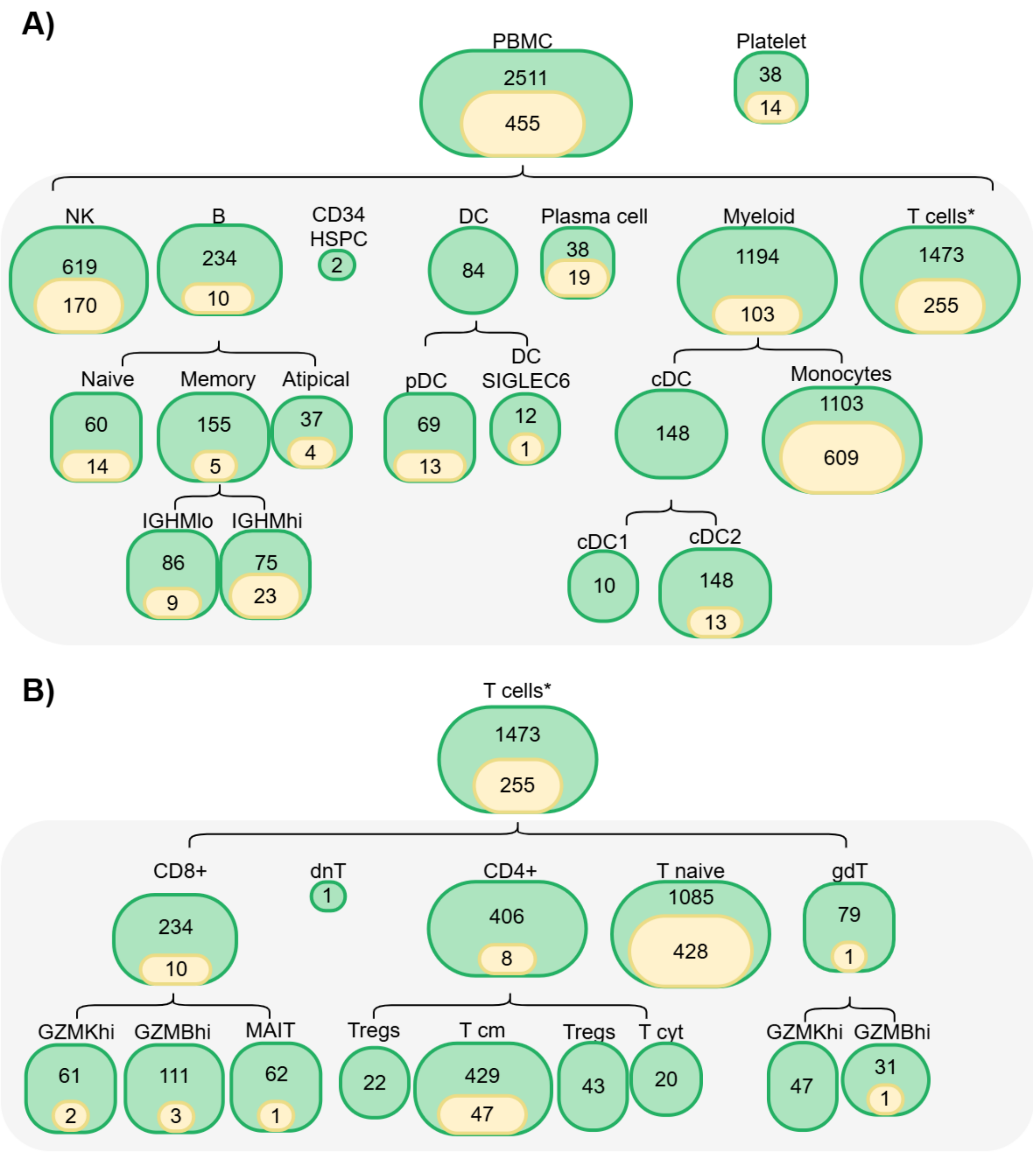

Figure S2: Number of significant ASVs detected at different levels of hierarchical cell type aggregation. The number in the green shape defines the number of found ASV, the yellow shape includes the number of unique found ASV per cell type (counts represent events unique to a node after subtracting all events from its parent nodes, so the nodes show only events not classified into any sub-type, highlighting the gain from broader aggregation). *A.* Main PBMC hierarchy without T cells branching. *B.* T cells branching.

Abramov, Sergey, Alexandr Boytsov, Daria Bykova, Dmitry D. Penzar, Ivan Yevshin, Semyon K. Kolmykov, Marina V. Fridman, et al. 2021. "Landscape of Allele-Specific Transcription Factor Binding in the Human Genome." *Nature Communications* 12 (1): 2751.

Aguet, François, Shankara Anand, Kristin G. Ardlie, Stacey Gabriel, Gad A. Getz, Aaron Graubert, Kane Hadley, et al. 2020. "The GTEx Consortium Atlas of Genetic Regulatory Effects across Human Tissues." *Science*, September. https://doi.org/10.1126/science.aaz1776.

Alam, Tanvir, Yulia A. Medvedeva, Hui Jia, James B. Brown, Leonard Lipovich, and Vladimir B. Bajic. 2014. "Promoter Analysis Reveals Globally Differential Regulation of Human Long Non-Coding RNA and Protein-Coding Genes." *PLOS ONE* 9 (10): e109443.

Amoah, Kofi, Yun-Hua Esther Hsiao, Jae Hoon Bahn, Yiwei Sun, Christina Burghard, Boon Xin Tan, Ei-Wen Yang, and Xinshu Xiao. 2021. "Allele-Specific Alternative Splicing and Its Functional Genetic Variants in Human Tissues." *Genome Research* 31 (3): 359.

Browne, Michael W., and Robert Cudeck. 1992. "Alternative Ways of Assessing Model Fit." *Sociological Methods & Research*, November. https://doi.org/10.1177/0049124192021002005.

Buyan, Andrey, Georgy Meshcheryakov, Viacheslav Safronov, Sergey Abramov, Alexandr Boytsov, Vladimir Nozdrin, Eugene F. Baulin, et al. 2025. "Statistical Framework for Calling Allelic Imbalance in High-Throughput Sequencing Data." *Nature Communications* 16 (1): 1–19.

Chen, Jieming, Joel Rozowsky, Timur R. Galeev, Arif Harmanci, Robert Kitchen, Jason Bedford, Alexej Abyzov, Yong Kong, Lynne Regan, and Mark Gerstein. 2016. "A Uniform Survey of Allele-Specific Binding and Expression over 1000-Genomes-Project Individuals." *Nature Communications* 7 (1): 1–13.

Cummings, Beryl B., Jamie L. Marshall, Taru Tukiainen, Monkol Lek, Sandra Donkervoort, A. Reghan Foley, Veronique Bolduc, et al. 2017. "Improving Genetic Diagnosis in Mendelian Disease with Transcriptome Sequencing." *Science Translational Medicine* 9 (386). https://doi.org/10.1126/scitranslmed.aal5209.

Di Tommaso, Paolo, Maria Chatzou, Evan W. Floden, Pablo Prieto Barja, Emilio Palumbo, and Cedric Notredame. 2017. "Nextflow Enables Reproducible Computational Workflows." *Nature Biotechnology* 35 (4): 316–19.

Frésard, Laure, Craig Smail, Nicole M. Ferraro, Nicole A. Teran, Xin Li, Kevin S. Smith, Devon Bonner, et al. 2019. "Identification of Rare-Disease Genes Using Blood Transcriptome Sequencing and Large Control Cohorts." *Nature Medicine* 25 (6): 911–19.

Ge, Bing, Dmitry K. Pokholok, Tony Kwan, Elin Grundberg, Lisanne Morcos, Dominique J. Verlaan, Jennie Le, et al. 2009. "Global Patterns of Cis Variation in Human Cells Revealed by High-Density Allelic Expression Analysis." *Nature Genetics* 41 (11): 1216–22.

Geijn, Bryce van de, Graham McVicker, Yoav Gilad, and Jonathan K. Pritchard. 2015. "WASP: Allele-Specific Software for Robust Molecular Quantitative Trait Locus Discovery." *Nature Methods* 12 (11): 1061–63.

George, E. O., and G. S. Mudholkar. 1983. "On the Convolution of Logistic Random Variables." *Metrika* 30 (1): 1–13.

Kim, Daehwan, Joseph M. Paggi, Chanhee Park, Christopher Bennett, and Steven L. Salzberg. 2019. "Graph-Based Genome Alignment and Genotyping with HISAT2 and HISAT-Genotype." *Nature Biotechnology* 37 (8): 907–15.

Kock, Kian Hong, Le Min Tan, Kyung Yeon Han, Yoshinari Ando, Damita Jevapatarakul, Ankita Chatterjee, Quy Xiao Xuan Lin, et al. 2025. "Asian Diversity in Human Immune Cells." *Cell* 188 (8): 2288–2306.e24.

Kurtzer, Gregory M., Vanessa Sochat, and Michael W. Bauer. 2017. "Singularity: Scientific Containers for Mobility of Compute." *PLOS ONE* 12 (5): e0177459.

Li, Heng, Bob Handsaker, Alec Wysoker, Tim Fennell, Jue Ruan, Nils Homer, Gabor Marth, Goncalo Abecasis, Richard Durbin, and 1000 Genome Project Data Processing Subgroup. 2009. "The

Sequence Alignment/Map Format and SAMtools." *Bioinformatics (Oxford, England)* 25 (16): 2078–79.

Liu, Zhi, Xiao Dong, and Yixue Li. 2018. "A Genome-Wide Study of Allele-Specific Expression in Colorectal Cancer." *Frontiers in Genetics* 9 (November):414764.

Li, Yun, Zheng Huang, Zhaojun Zhang, Qifei Wang, Fengxian Li, Shufang Wang, Xin Ji, Shaokun Shu, Xiangdong Fang, and Lan Jiang. 2023. "FIPRESCI: Droplet Microfluidics Based Combinatorial Indexing for Massive-Scale 5′-End Single-Cell RNA Sequencing." *Genome Biology* 24 (1): 1–32.

Martin, Marcel. 2011. "Cutadapt Removes Adapter Sequences from High-Throughput Sequencing Reads." *EMBnet.journal* 17 (1): 10–12.

Mattevi, Stefania, Francesco Mazzarotto, and Paolo Martini. 2025. "Allele-Specific Expression Analysis: Pipelines, Applications, Challenges, and Unmet Needs." *Computers in Biology and Medicine* 196 (September):110890.

Maurano, Matthew T., Eric Haugen, Richard Sandstrom, Jeff Vierstra, Anthony Shafer, Rajinder Kaul, and John A. Stamatoyannopoulos. 2015. "Large-Scale Identification of Sequence Variants Influencing Human Transcription Factor Occupancy in Vivo." *Nature Genetics* 47 (12): 1393–1401.

Miller, Brecca R., Alison M. Morse, Jacqueline E. Borgert, Zihao Liu, Kelsey Sinclair, Gavin Gamble, Fei Zou, et al. 2021. "Testcrosses Are an Efficient Strategy for Identifying Cis-Regulatory Variation: Bayesian Analysis of Allele-Specific Expression (BayesASE)." *G3 Genes|Genomes|Genetics* 11 (5): jkab096.

Ratliff, Simeon. 2025. "Docker: Accelerated Container Application Development." Docker. Simeon Ratliff. April 9, 2025. https://www.docker.com/.

Santiago, Ines de, Wei Liu, Ke Yuan, Martin O'Reilly, Chandra Sekhar Reddy Chilamakuri, Bruce A. J. Ponder, Kerstin B. Meyer, and Florian Markowetz. 2017. "BaalChIP: Bayesian Analysis of Allele-Specific Transcription Factor Binding in Cancer Genomes." *Genome Biology* 18 (1): 1–17.

Smith, Tom, Andreas Heger, and Ian Sudbery. 2017. "UMI-Tools: Modeling Sequencing Errors in Unique Molecular Identifiers to Improve Quantification Accuracy." *Genome Research* 27 (3): 491–99.

Stachowiak, Monika, Izabela Szczerbal, and Krzysztof Flisikowski. 2018. "Investigation of Allele-Specific Expression of Genes Involved in Adipogenesis and Lipid Metabolism Suggests Complex Regulatory Mechanisms of PPARGC1A Expression in Porcine Fat Tissues." *BMC Genetics* 19 (1): 1–9.

Vierstra, Jeff, John Lazar, Richard Sandstrom, Jessica Halow, Kristen Lee, Daniel Bates, Morgan Diegel, et al. 2020. "Global Reference Mapping of Human Transcription Factor Footprints." *Nature* 583 (7818): 729–36.

Click here to access/download;Figure;fig_s1.png

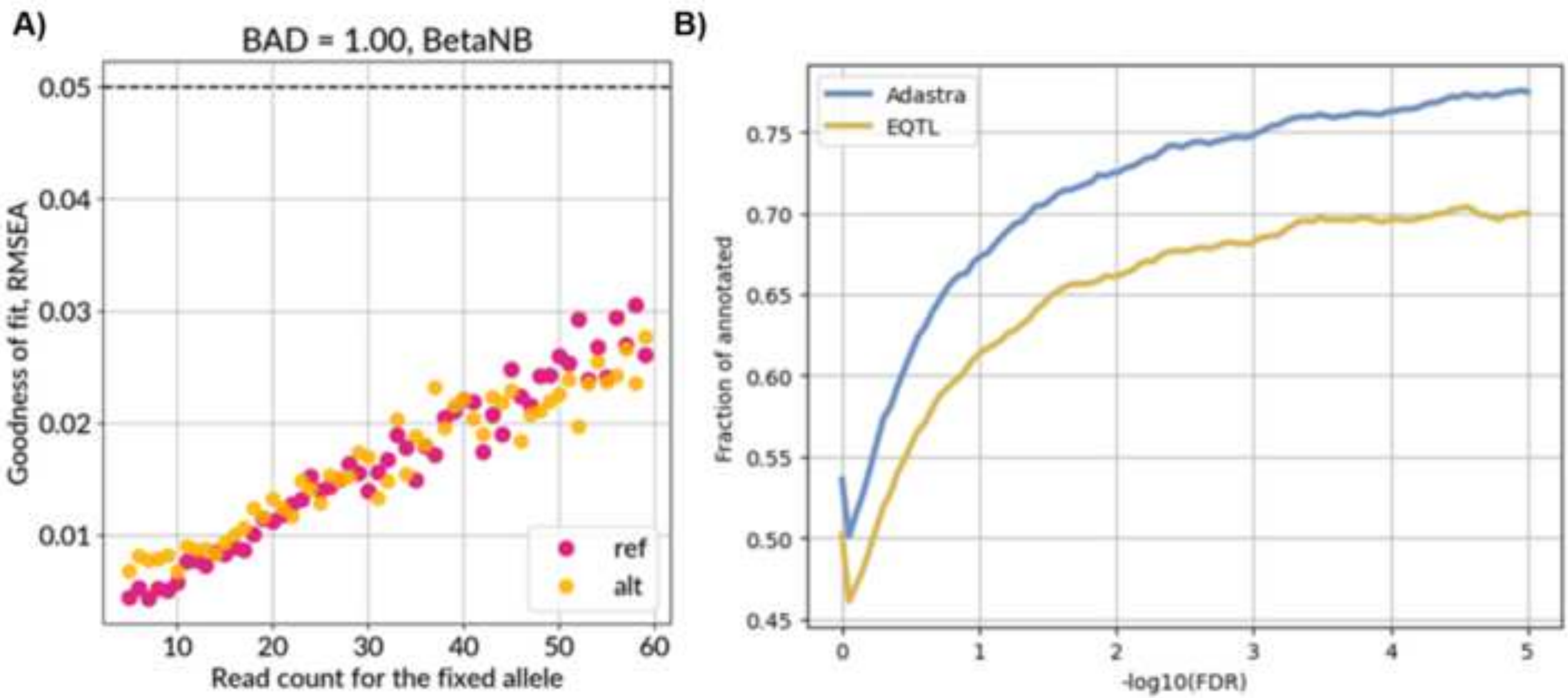

Click here to access/download;Figure;fig_4.png

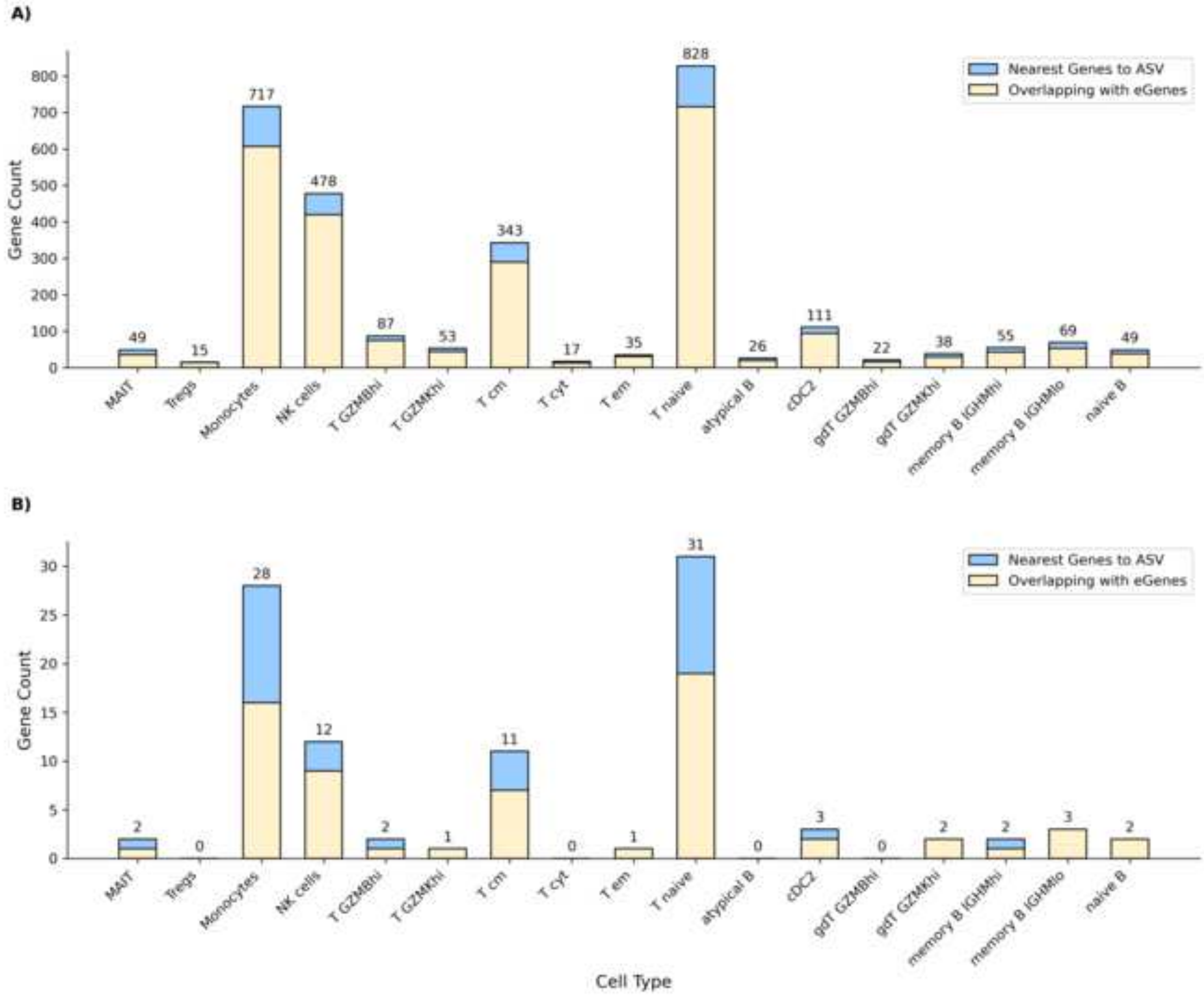

Click here to access/download;Figure;fig_1.png

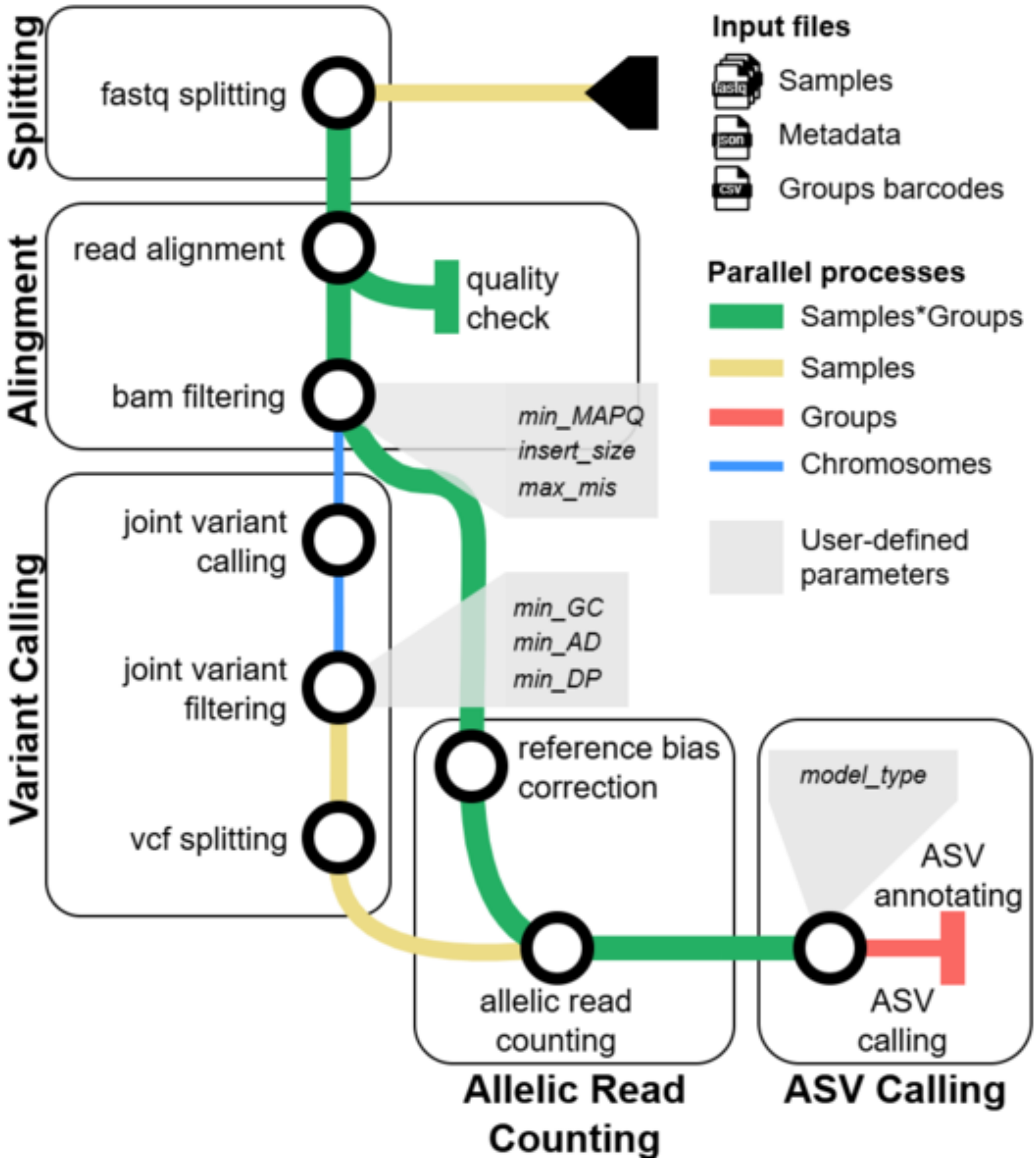

Click here to access/download;Figure;fig_3.png

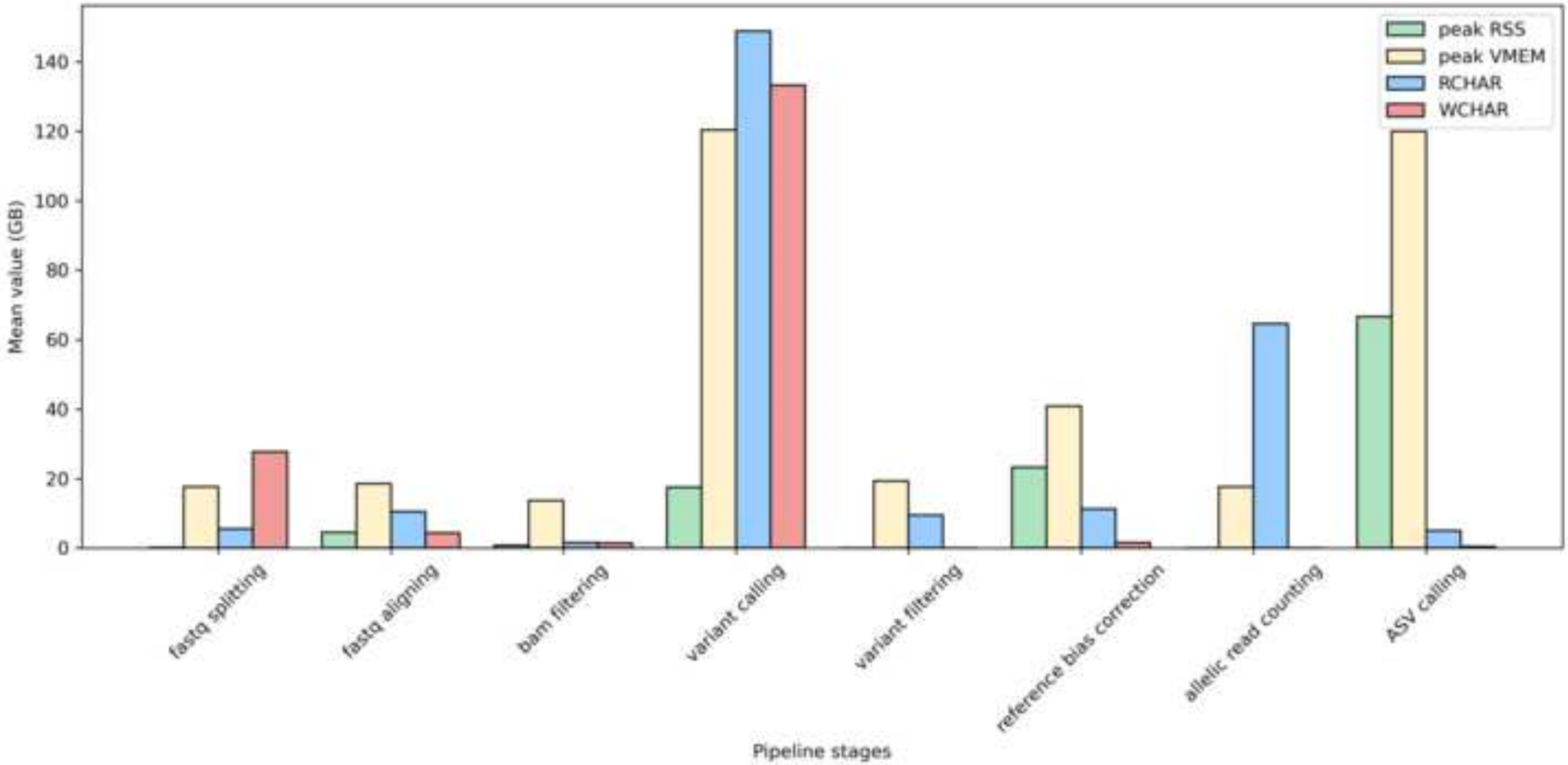

Click here to access/download;Figure;fig_2.png

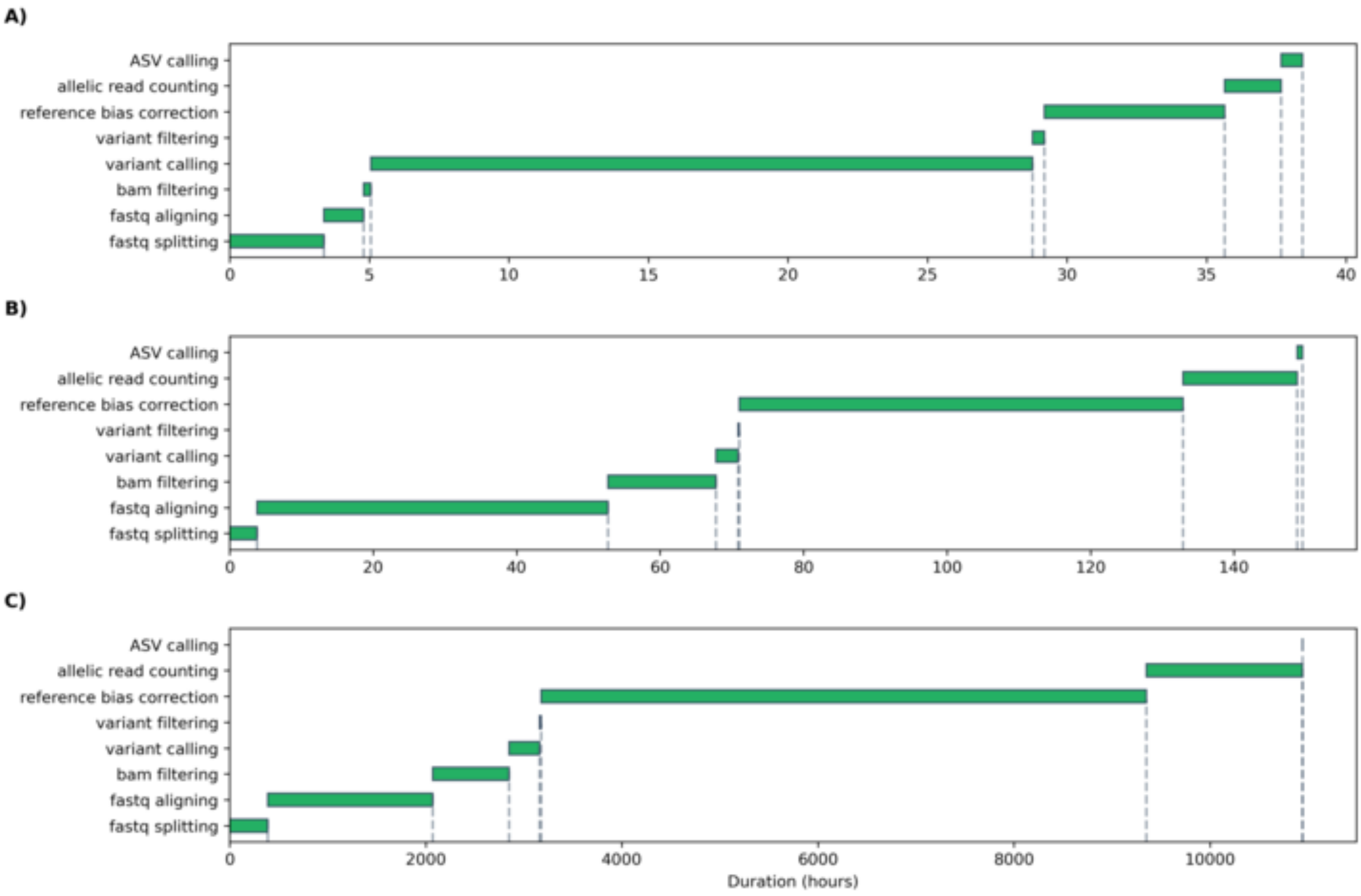

Click here to access/download;Figure;fig_s2.png

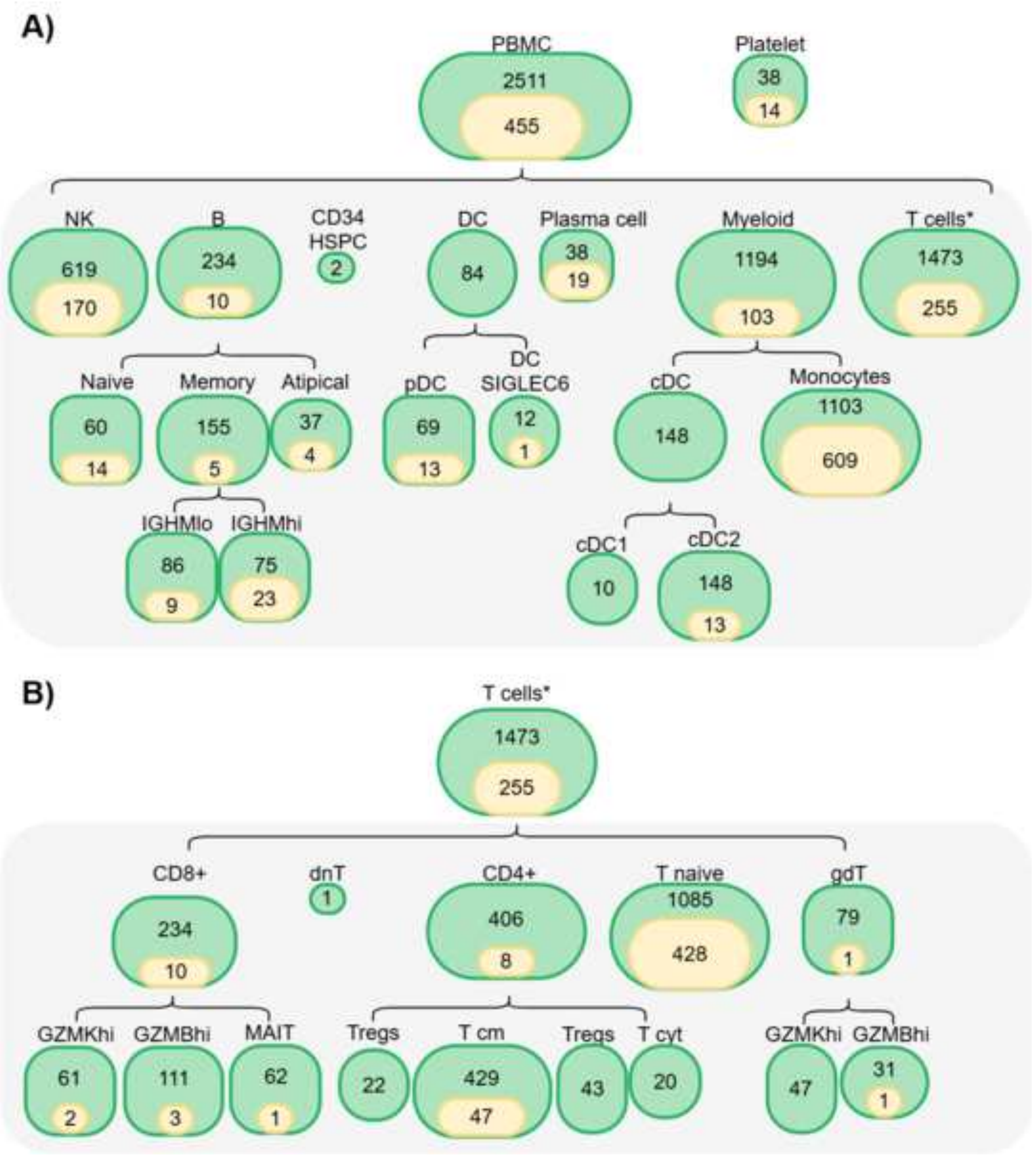

Supplement: giag042_GIGA-D-25-00386_original_submission [file giag042_giga-d-25-00386_original_submission.pdf]
