# Supplementary material for: NEXT-scASV: a Nextflow pipeline for allele-specific variant calling from single-cell RNA-seq data
Source: Gigascience. 2026 Apr 6;15:giag042. doi: 10.1093/gigascience/giag042 (PMC13148397; doi:10.1093/gigascience/giag042)
Supplement: giag042_GIGA-D-25-00386_Revision_1 [file giag042_giga-d-25-00386_revision_1.pdf]

GigaScience

NEXT-scASV: A Nextflow Pipeline for Allele-Specific Variants Calling from single cell RNA-seq data

--Manuscript Draft--

**Manuscript Number:**	GIGA-D-25-00386R1
**Full Title:**	NEXT-scASV: A Nextflow Pipeline for Allele-Specific Variants Calling from single cell RNA-seq data
**Article Type:**	Technical Note
**Funding Information:**	RSF (23-14-00371) — Mrs. Yulia Medvedeva<br>MSHERF (075-15-2025-014) — Mr. Ivan Kulakovskiy<br>FFRW (FFRW-2025-010) — Mr. Ivan Kulakovskiy
**Abstract:**	## Abstract<br><br>The rapid accumulation of single-cell sequencing data presents major computational challenges in reproducibility, scaling, and handling data imperfections like sparsity and technical variations, which complicate even basic analyses. The next level of complexity is the allele-specific analysis, focused on identifying differential gene expression or regulation between homologous chromosomes by estimating the allelic imbalance of read counts at individual single-nucleotide variants.<br>Here we present NEXT-scASV, a scalable Nextflow pipeline for calling allele-specific variants (ASVs) from 5′ single-cell RNA sequencing data. NEXT-scASV automates the entire process—from read alignment and quality control to variant calling and statistical evaluation of the allelic imbalance—within a containerized environment, ensuring reproducibility and ease of deployment across platforms. NEXT-scASV is able to perform de novo ASV detection from single-cell sequencing data without prior genotyping. Its modular design allows for massive parallelization, efficiently handling the scale of modern atlas-level studies.<br>We validate NEXT-scASV on a dataset of 135K peripheral blood mononuclear cells (PBMCs) from 57 donors, demonstrating that it processes large-scale data efficiently, completing analysis in a week on a cluster with one node and 100 threads. Crucially, the pipeline reliably identifies ASVs even in rare cell populations (e.g. gdT GZMBhi and memory B IGHMhi cells), which remains elusive for bulk analyses. We also successfully detect allele-specific regulation of long non-coding RNAs and other lowly expressed, cell type-specific genes. Genes linked to detected ASVs show a high concordance (80%) with previously reported eQTLs. This strong validation confirms that NEXT-scASV produces biologically relevant results, making it a powerful tool for uncovering allele-specific regulation in large-scale, complex single-cell studies.
**Corresponding Author:**	Andrey Vadimovich Shevtsov, M.D.<br>Federal Research Centre Fundamentals of Biotechnology: FGU Federal'nyj issledovatel'skij centr Fundamental'nye osnovy biotehnologii Rossijskoj akademii nauk<br>Moscow, RUSSIAN FEDERATION
**Corresponding Author Secondary Information:**	
**Corresponding Author's Institution:**	Federal Research Centre Fundamentals of Biotechnology: FGU Federal'nyj issledovatel'skij centr Fundamental'nye osnovy biotehnologii Rossijskoj akademii nauk
**Corresponding Author's Secondary Institution:**	
**First Author:**	Andrey Shevtsov
**First Author Secondary Information:**	
**Order of Authors:**	Andrey Shevtsov<br>Andrey Boyan

Powered by Editorial Manager® and ProduXion Manager® from Aries Systems Corporation

	Vladimir Nozdrin
	Pavel Akhtyamov
	Alexei Stupnikov
	Georgy Meshcheryakov
	Ivan Kulakovskiy
	Yulia Medvedeva
**Order of Authors Secondary Information:**	
**Response to Reviewers:**	Reviewer #1:<br><br>1. In the Abstract, on line 2, I strongly recommend avoiding the term "imperfections" when referring to characteristics or biases in single-cell data. A more neutral and precise term would be more appropriate.<br><br>*Answer:<br>Thank you for the recommendation. We changed the term “imperfections” to the more natural “characteristics”. Updated version: “The rapid accumulation of single-cell sequencing data presents major computational challenges in reproducibility, scaling, and handling data characteristics like sparsity.”<br><br>2. Although the pipeline uses containerization to ensure reproducibility, it is good practice to explicitly list all the major software tools used (e.g., cutadapt v5, HISAT2, UMI-tools, bcftools, WASP, MIXALIME) and their exact versions in the Methods section or at least in the GitHub repository or supplementary materials, to ensure full transparency.<br><br>*Answer:<br>We totally agree, software versions are very important for reproducibility. We added major package versions to the supplementary data (Package versions). We also have an environment configuration file in our repository (as_env.yaml) with all the package versions, except WASP. WASP needs a patch, so we describe its installation in the README.<br><br>3. Regarding the selection of tools, I am concerned about the criteria used, particularly the choice of HISAT2 over other widely used aligners (such as STAR) for scRNA-seq, and bcftools over other variant callers such as GATK HaplotypeCaller. The authors should better justify these decisions, especially considering the known challenges and biases in RNA-seq variant calling, even in bulk data, which is substantially less sparse than single-cell datasets.<br><br>*Answer:<br>We selected HISAT2 over STAR due to its superior speed, a critical factor when processing large single-cell datasets with multiple donors and cell types. This choice does not compromise accuracy: on a dataset with a natural human polymorphism rate of 0.001, both aligners delivered nearly identical performance, achieving near 100% precision and over 95% recall (Baruzzo et al. 2016).<br><br>The choice of variant caller determines the initial set of candidate SNPs and therefore establishes the upper bound for sensitivity and accuracy in downstream analysis. However, in our pipeline, the caller's impact is mitigated through several subsequent steps. We primarily use the caller for initial SNP discovery, after which we apply rigorous variant-level filters, including genotype quality (GQ), read depth (DP), and allelic depth (AD). Additionally, we implement read-level filters before counting, such as mapping quality (MAPQ), edit distance (NM), and exclusion of QC-failed reads using filter_reads.py and samtools (flag 512). Crucially, the final allele-specific expression (ASE) signal is derived from direct allele-specific read counting in BAM files using count_tags_pileup.py, rather than relying on caller-derived quality scores. Furthermore, variants are called at the patient level, which leverages information across related samples to address the sparse coverage typical of single-cell RNA-seq data. As a result, the final ASE estimates are substantially less dependent on the specific variant caller than in workflows where caller outputs directly determine allelic estimates. For

Powered by Editorial Manager® and ProduXion Manager® from Aries Systems Corporation

this study, we selected bcftools as our variant caller, as it offers a simpler, faster, and more computationally lightweight solution while still providing robust candidate variants suitable for downstream ASE analysis.

We also add these rationales into the Methods: Alignment Subflow and Variant Calling Subflow
sections.

4. In the Background section, the manuscript states that "very few existing tools allow for this type of analysis". However, tools such as SCALE and scBASE were developed with related goals. The authors should therefore compare NEXT-scASV with these existing approaches and clarify what advantages it provides.

*Answer:
We thank the reviewer for the suggestion. While MixALime was extensively benchmarked in the original paper, including comparisons with other bulk-focused tools, benchmarking against single-cell methods is also important for assessing our pipeline. We agree that SCALE and scBASE address related questions, but they are not directly comparable to our setting and output. First, SCALE was developed primarily for gene-level allele-specific expression analysis (e.g., allelic expression per gene and cell-type/state), whereas NEXT-scASV focuses on variant-level allele-specific variants (ASVs). Because the input features, statistical targets, and outputs differ (gene-level vs. SNP-level events), a direct head-to-head comparison would not be methodologically meaningful. Second, scBASE requires phased variants/haplotypes to assign allelic counts to parental haplotypes and to perform its downstream analyses. In our setting, reliable phasing information is not available for the studied data, and NEXT-scASV is designed to operate without requiring phased genotypes, which is an important practical advantage. To provide a relevant comparison within the same variant-level scope, we instead evaluated two widely used tools with closely related goals, scDALI and DAESC. We find that scDALI provides a conservative subset of MixALime calls, while DAESC can expand the analysis by highlighting variants with context-dependent allelic effects. We add a new paragraph, “ASV tools comparison” to the results section with comparison details.

We revised the Background text to more precisely state that few existing tools enable variant-level ASV calling in scRNA-seq without requiring phased genotypes, and discuss SCALE and scBASE in this context:

“Despite these advantages, single-cell RNA-seq data are sparse: most variants are covered by few reads per cell, and many cells show dropout at a given locus. To increase power, some single-cell allele-specific approaches therefore rely on different aggregation strategies, most commonly at the gene level, where allelic counts are summed across sites within a gene and tested for gene-level ASE (Choi et al. 2019; Jiang et al. 2017). In this work, we focus on variant-level ASVs rather than gene-level aggregation, because regulatory effects are often driven by specific nucleotide changes. A single variant can disrupt a transcription factor motif, alter promoter or enhancer activity, or create a splice-altering event, leading to a mechanistic and directly interpretable signal. Variant-level results also enable direct overlap with external catalogs of regulatory variants, and they avoid masking situations where multiple variants within the same gene have different, context-dependent effects. Several single-cell methods have been proposed for allele-specific analysis. SCALE (Jiang et al. 2017) is designed for gene-level ASE modeling from sparse scRNA-seq counts. scBASE (Choi et al. 2019) integrates allele-specific signals with phased haplotypes, which typically requires phased genotypes. Tools that can operate at the variant level using allelic counts: DAESC (Qi et al. 2023) and scDALI (Heinen et al. 2022). DAESC is formulated to detect differential allelic imbalance between groups or conditions, whereas scDALI supports tests for both differential effects (heterogeneous mode) and shared allele-specific effects (homogeneous mode), as well as a joint setting that combines information across modes.
”

Powered by Editorial Manager® and ProduXion Manager® from Aries Systems Corporation

5. MIXALIME is a key component of the pipeline, as it models allelic read counts using the Beta-Negative Binomial framework. However, the original MIXALIME methodology was primarily validated on bulk datasets including RNA-seq, CAGE-seq or ATAC-seq, as well as on simulated data, not on highly sparse single-cell data. Therefore, its direct applicability to scRNA-seq therefore requires additional justification. The manuscript would be strengthened by providing validation results specifically on single-cell data, or at least a detailed explanation of why the method is expected to perform robustly in this context. This should include any modifications or parameter adjustments relative to the original implementation.

*Answer:
In NEXT-scASV, MIXALIME is applied to allele-specific read counts aggregated into pseudo-bulk profiles for each sample-by-cell-type group, rather than to sparse per-cell matrices. This aggregation shields the model from single-cell dropout effects; residual scRNA-seq variability manifests primarily as overdispersion in read depth, which is explicitly captured by the Beta-Negative Binomial (BetaNB) framework. As orthogonal validation, we assessed the external support of detected signals: over 60% of significant ASVs overlap previously reported regulatory variants (Figure S1 B), including allele-specific transcription factor binding events from ADASTRA and cis-eQTLs from GTEx. These enrichments support the biological plausibility and robustness of our calls in the scRNA-seq context.

6. The results were validated for biological significance using the AIDA cohort study. On average, 82% of the ASV-associated genes identified by the pipeline were also reported in the original study. While this suggests good specificity, the manuscript does not adequately discuss sensitivity. What proportion of total known eGenes is recovered by this approach? The authors also state that the remaining ~18% of genes "likely represent a mix of false positives and, more interestingly, true cell-type-specific regulatory events". Since the AIDA study also involved single-cell-level detection, the meaning of "cell type-specific regulatory events" should be clarified and contextualized.

*Answer:
We agree that the overlap of ASV-associated genes with the original AIDA results primarily reflects specificity and that sensitivity should be discussed explicitly. In our current analysis, we intentionally processed only a subset of the AIDA cohort (≈50 out of ≈300 individuals), because the goal of this section was to validate the pipeline behavior (end-to-end calling, bias correction, and statistical modeling) rather than to provide a final biological catalog. As expected, this design yields a lower recovery of the full set of known eGenes: 1) statistical power is reduced due to smaller sample size, and 2) allele-specific approaches are intrinsically limited to loci with expressed heterozygous variants covered by reads, so regulatory variants acting through distal mechanisms or variants not captured in the transcribed/read-covered regions cannot be recovered by construction. Importantly, this does not undermine the utility of ASE/ASV analysis: ASE provides a within-individual, allele-contrast signal that is complementary to eQTL mapping and is particularly informative for detecting cis-regulatory effects in a cell-type-resolved manner while being less sensitive to between-individual confounders. We will add a sensitivity estimate (fraction of known eGenes recovered in our subset; shown in a new Supplementary figure S5) and clarify terminology.
By “cell-type-specific regulatory events” we mean genes showing significant allelic imbalance in a particular cell type (or cell-type group) but not in others, consistent with cell-type-restricted regulatory activity and/or differences in expression/coverage across cell types. Although the AIDA study also performed single-cell-level detection, cell-type specificity in our context refers specifically to differential presence/strength of allelic imbalance across annotated cell types within the same cohort, rather than to novelty relative to bulk catalogs; accordingly, the remaining ~18% likely comprises both false positives and bona fide cell-type-restricted effects that are underpowered or averaged out in global/aggregated analyses.

7. Importantly, I attempted to follow the GitHub instructions to install and run the pipeline, but I was unable to do so. The Docker build fails because line 13 of the Dockerfile specifies "RUN chmod +x /app/run_workflow.sh && chmod +x /app/run.sh && chmod +x /app/bin/nextflow" yet the files run_workflow.sh and run.sh do not exist in the repository. I am not sure whether this is a general issue or if additional steps are

required, but this must be corrected — either by updating the repository or by clarifying the installation instructions.

*Answer:
Thank you, we corrected the Dockerfile and updated the instructions.

8. Finally, I believe the pipeline would greatly benefit from the inclusion of a small test dataset and a brief tutorial. This would enable users to evaluate the workflow's functionality and performance before applying it to large datasets.

*Answer:
We fully agree that a small test dataset and tutorial are important. We prepared a small subset of the public dataset that we used for evaluation, including data from 2 patients and 14 cell types. We provide a download link to this dataset and add a brief step by step tutorial so users can quickly test the workflow and check its performance before running it on large datasets.

Reviewer #2:

1. On page 13, line 9, Figure 3B is referenced but does not appear in the manuscript.

*Answer:
Corrected “Figure 3B” -> “Figure 4B”

2. The first paragraph of Discussion section is left-aligned and inconsistent with the formatting of the surrounding text.

*Answer:
Fixed the alignment in the discussion section.

3. Suggestion for improvement: The Background section could be strengthened by summarizing existing single-cell ASV detection models and briefly comparing them with MIXALIME (Buyan et al. 2025) and WASP tool (van de Geijn et al. 2015), which are used within the NEXT-scASV pipeline. This context would help better position the contribution of the current work within the landscape of single-cell allele-specific variation analysis.

*Answer:
Thank you for this suggestion. In the revised manuscript, we add a short overview of existing single-cell allele-specific models and clarify how they relate to the tools used in our pipeline. To provide empirical context, we include our benchmark against scDALI and DAESC, showing that scDALI recovered largely the same variants as MixALime, but in smaller numbers. In contrast, DAESC identified additional, tool-specific variants, as it appears complementary to our general speciality detection approach. We placed the detailed analysis in the paragraph “ASV tools comparison” of the results section.

Reviewer #3:

1. A major concern regarding this manuscript is the lack of comparative benchmarking against existing tools. Although the manuscript states that "very few existing tools allow for this kind of analysis," it does not provide a comparison of NEXT-scASV with any of these tools. Even if direct "apples-to-apples" comparisons prove challenging, the variant calling component should be benchmarked against other single-cell variant callers, such as cellsnp-lite and Monopogen.

*Answer:
Thank you for raising this point. We agree that benchmarking is important. For benchmarking the ASV-calling component, the most relevant existing approaches are scDALI and DAESC, which directly test allele-specific signals using single-cell allelic counts. We therefore compared NEXT-scASV/MixALime-based ASV calls to scDALI

Powered by Editorial Manager® and ProduXion Manager® from Aries Systems Corporation

and DAESC in two input regimes: (i) a pseudobulk setup matching our pipeline (sample × group aggregation) and (ii) a true single-cell setup using per-barcode allele counts. In pseudobulk mode, scDALI and DAESC largely overlapped with MixALime calls, while MixALime reported more ASVs, consistent with its focus on detecting overall allelic imbalance rather than differential effects. In true single-cell mode, scDALI and DAESC detected substantially more ASVs (DAESC: 31 → 188; scDALI: 23 → 130). DAESC reported additional unique variants, reflecting its sensitivity to context-dependent allelic effects, in contrast to MiXalime that aims to detect general allele-specific events. Importantly, ASVs detected by all three approaches showed substantial overlap with known regulatory variant resources (GTEx cis-eQTLs and ADASTRA), supporting the biological relevance of the calls.

In NEXT-scASV, the variant caller is mainly used to generate an initial set of candidate SNPs. The final allele-specific signal is derived from direct allele-specific read counting after alignment and bias correction (WASP), followed by additional variant- and read-level filtering and statistical testing in MixALime. As a result, the final set of significant ASVs is less dependent on the particular caller than in workflows where caller-derived genotypes directly define allelic estimates.
Monopogen achieves improved sensitivity by leveraging high-quality haplotypes and linkage disequilibrium information, which can substantially limit applicability when such reference resources are not available or not well matched to the cohort. In our setting, we did not have suitable haplotype/LD resources required for Monopogen, and therefore could not include it as a practical comparator.
To provide an empirical benchmark, we compared bcftools with cellsnp-lite. Cellsnp-lite reported more candidate variants, but most additional variants did not pass downstream ASV significance testing in MixALime. This suggests that cellsnp-lite is more sensitive to low-coverage variants, but this increased sensitivity does not translate into a clear advantage in terms of final significant ASVs (Figure S3). At the same time, each caller contributed a little fraction of caller-specific variants.

In the revised version, we (1) rewrite the statement “very few tools” by explicitly describing existing tools and their scope, (2) include the scDALI/DAESC benchmarking results in the Results:ASV tools comparison, and add the comparison with cellsnp-lite

2. Furthermore, could the authors elucidate why they opted to physically split FASTQ files instead of employing Read Groups within merged BAM files, a standard approach in handling multiplexed data for variant calling, such as in GATK workflows? If splitting is necessary for parallelization of bcftools in this context, the authors should discuss the rationale for choosing between virtual splitting and sequential processing, to prevent excessive input/output operations.

*Answer:
We split FASTQ files because our workflow runs several steps per sample, including filtering, alignment, and allele counting, and these steps are much faster and simpler when each sample is processed as its own unit. Using read groups in one merged BAM is common in GATK-style variant calling, but in our case, we would still need to separate data by group for downstream counting and quality control, which would mean repeatedly scanning or extracting parts of a large BAM and reusing large indexes, often creating more total input-output and more file system pressure than an early split. Splitting once at the beginning creates smaller files, enables clean parallel processing with predictable memory and runtime per job, and avoids contention when many tasks read the same big BAM at the same time. We therefore chose physical splitting as a practical trade-off: a small one-time input-output cost up front, but lower total input-output and shorter runtime across the full pipeline compared to virtual splitting or sequential processing.

3. A minor point is that this manuscript extensively emphasizes the advantages of 5' scRNA-seq; however, the majority of publicly available single-cell datasets utilize 3' scRNA-seq. The authors should consider discussing the potential limitations—if any—when applying their method to 3' scRNA-seq data, or explicitly state this limitation to appropriately set user expectations.

*Answer:
5’ scRNA-seq data reads are enriched near the transcription start site. Therefore, it

	provides better coverage of regulatory regions and nearby heterozygous variants that can act as causal regulatory SNPs. When the same workflow is applied to 3 prime scRNA-seq data, the main limitation is that coverage is shifted to the transcript end, so many candidate regulatory variants will not be sequenced, and the number of informative SNPs per gene and per cell can be much lower. As a result, both sensitivity and power to detect allele-specific effects are expected to decrease, and it can be difficult to identify causal rSNPs and even to assess validity because 3 prime datasets often do not capture the variants that are present in external allele-specific variant resources. We will add this point to the manuscript to clarify that 3 prime data can still be processed, but results may be less complete
**Additional Information:**	
**Question**	**Response**
Are you submitting this manuscript to a special series or article collection?	No
**Experimental design and statistics**<br><br>Full details of the experimental design and statistical methods used should be given in the Methods section, as detailed in our Minimum Standards Reporting Checklist. Information essential to interpreting the data presented should be made available in the figure legends.<br><br>Have you included all the information requested in your manuscript?	Yes
**Resources**<br><br>A description of all resources used, including antibodies, cell lines, animals and software tools, with enough information to allow them to be uniquely identified, should be included in the Methods section. Authors are strongly encouraged to cite Research Resource Identifiers (RRIDs) for antibodies, model organisms and tools, where possible.<br><br>Have you included the information requested as detailed in our Minimum Standards Reporting Checklist?	Yes
**Availability of data and materials**<br><br>All datasets and code on which the	Yes

conclusions of the paper rely must be either included in your submission or deposited in publicly available repositories (where available and ethically appropriate), referencing such data using a unique identifier in the references and in the “Availability of Data and Materials” section of your manuscript.<br><br>Have you have met the above requirement as detailed in our Minimum Standards Reporting Checklist?	
GigaScience has policies and guidelines in place for the use of generative AI-writing tools such as ChatGPT. If you have used such writing tools to assist with writing the manuscript this must be declared and cited in the text. Authors should not list AI-writing tools and other AI-assisted technologies as an author or co-author and should acknowledge that they are fully responsible for text generated or refined by AI-writing tools.<p><br><br>A summary of use (particularly in the introduction or among methods) needs to be included at the end of the paper, and the outputs should also be included as a supplementary file hosted in GigaDB or other open repositories. Please read our guidelines for more information. <p><br><br>By submitting to GigaScience, you are aware of the journal's AI-writing tools policy, and if you have declared use of such tools below, you have acknowledged this where appropriate in your manuscript and have made a summary of use and outputs available. <p><br><b>AI-assisted writing tools have been used in the preparation of this manuscript?	No

Powered by Editorial Manager® and ProduXion Manager® from Aries Systems Corporation

Manuscript Click here to access/download;Manuscript;next-asv_final_version (1).docx

Title

NEXT-scASV: A Nextflow Pipeline for Allele-Specific Variants Calling from single cell RNA-seq data.

Authors

Andrey Shevtsov [1,2] (ORCID: 0009-0002-2737-6609), Andrey Buyan [3], Vladimir Nozdrin [4], Pavel Akhtyamov [5, 6], Alexei Stupnikov [1, 7], Georgy Meshcheryakov [3], Ivan V. Kulakovskiy [3, 7, 8] & Yulia A. Medvedeva [1,9] (ORCID: 0000-0002-7587-1666)

1- Research Institute of Biotechnology, Russian Academy of Sciences
2- Bioinformatics Group, AIRI, Moscow 121170, Russia
3- Institute of Protein Research, Russian Academy of Sciences, Pushchino, Russia
4- Faculty of Bioengineering and Bioinformatics, Lomonosov Moscow State University, Moscow, Russia
5- Moscow Center for Advanced Studies, Department of Biomedical Physics, Moscow, 123592, Russia
6- National Research University Higher School of Economics, 11 Pokrovksy Bulvar, Moscow, 109028, Russia
7- Vavilov Institute of General Genetics, Russian Academy of Sciences, Moscow, Russia
8- Institute of Biochemistry and Genetics, Ufa Federal Research Centre of the Russian Academy of Sciences, Ufa, Russia
9- Mohamed Bin Zayed University of Artificial Intelligence, Abu Dhabi, UAE

Corresponding authors:
Andrey Shevtsov
Yulia A. Medvedeva

ABSTRACT

The rapid accumulation of single-cell sequencing data presents major computational challenges in reproducibility, scaling, and handling data characteristics like sparsity and technical variations, which complicate even basic analyses. The next level of complexity is the allele-specific analysis, focused on identifying differential gene expression or regulation between homologous chromosomes by estimating the allelic imbalance of read counts at individual single-nucleotide variants.

Here we present NEXT-scASV, a scalable Nextflow pipeline for calling allele-specific variants (ASVs) from 5′ single-cell RNA sequencing data. NEXT-scASV automates the entire process—from read alignment and quality control to variant calling and statistical evaluation of the allelic imbalance—within a containerized environment, ensuring reproducibility and ease of deployment across platforms. NEXT-scASV is able to perform *de*

novo ASV detection from single-cell sequencing data without prior genotyping. Its modular design allows for massive parallelization, efficiently handling the scale of modern atlas-level studies.

We validate NEXT-scASV on a dataset of 135,000 peripheral blood mononuclear cells (PBMCs) from 57 donors, demonstrating that it processes large-scale data efficiently, completing analysis in a week on a cluster with one node and 100 threads. Crucially, the pipeline reliably identifies ASVs even in rare cell populations (e.g. gdT GZMBhi and memory B IGHMhi cells), which remains elusive for bulk analyses. We also successfully detect allele-specific regulation of long non-coding RNAs and other lowly expressed, cell type-specific genes. Genes linked to detected ASVs show a high concordance (80%) with previously reported eQTLs. This strong validation confirms that NEXT-scASV produces biologically relevant results, making it a powerful tool for uncovering allele-specific regulation in large-scale, complex single-cell studies.

KEYWORDS: allele-specific variants, single-cell RNA-seq, Nextflow, computational pipeline, regulatory variants

BACKGROUND

Genetic variation within regulatory elements represents a fundamental mechanism of phenotypic diversity and disease susceptibility [1,2]. In diploid genomes, particular regulatory variants can be identified from comparison of allelic signals at homologous chromosomes, revealing allele-specific gene expression (ASE), transcription factor binding (ASB), and chromatin accessibility (ASA), representing different types of allele-specific variants (ASVs).

Allele-specific variants have been widely studied using bulk sequencing [3–7], which yields averaged signals across often heterogeneous cell populations. The averaging obscures cell type-specific effects, potentially diluting the signal from biologically meaningful, context-dependent regulation [8,9]. The advent of single-cell sequencing (including scRNA-seq) provides the resolution necessary to interrogate ASVs and ASE within defined cell types [10,11].

5′ scRNA-seq offers a distinct advantage for allele-specific analysis. By capturing the 5′ ends of transcripts, scRNA-Seq reads are strongly enriched in regulatory regions in the close vicinity to the transcription start sites (TSSs), such as promoters and transcribed enhancers, physically linking genetic variants to the expression of their target genes on the level of individual reads [12]. This allows for direct and unambiguous assignment of regulatory effects compared to 3′ scRNA-seq protocols, which require imputation or statistical phasing. Furthermore, the high sensitivity afforded by cell type-specific analysis and the direct capture of 5' ends opens the possibility of investigating allele-specific regulation in challenging genomic contexts. These include long non-coding RNAs (lncRNAs) and other lowly expressed or highly tissue-specific genes, which are often underrepresented

in bulk tissue eQTL studies due to signal dilution across heterogeneous cell populations. Notably, the promoters of lncRNAs often contain highly specific genetic features [13] that are susceptible to disruption by genetic variants. This underscores the unique advantage of 5' scRNA-seq, and by extension our pipeline, for directly interrogating the allele-specific mechanisms governing lncRNA expression.

Despite these advantages, single-cell RNA-seq data are sparse: most variants are covered by few reads per cell, and many cells show dropout at a given locus. To increase power, some single-cell allele-specific approaches therefore rely on different aggregation strategies, most commonly at the gene level, where allelic counts are summed across sites within a gene and tested for gene-level ASE [14,15]. In this work, we focus on variant-level ASVs rather than gene-level aggregation, because regulatory effects are often driven by specific nucleotide changes. A single variant can disrupt a transcription factor motif, alter promoter or enhancer activity, or create a splice-altering event, leading to a mechanistic and directly interpretable signal. Variant-level results also enable direct overlap with external catalogs of regulatory variants, and they avoid masking situations where multiple variants within the same gene have different, context-dependent effects.

Several single-cell methods have been proposed for allele-specific analysis. SCALE [15] is designed for gene-level ASE modeling from sparse scRNA-seq counts. scBASE [14] integrates allele-specific signals with phased haplotypes, which typically requires phased genotypes. Tools that can operate at the variant level using allelic counts: DAESC [16] and scDALI [17]. DAESC is formulated to detect differential allelic imbalance between groups or conditions, whereas scDALI supports tests for both differential effects (heterogeneous mode) and shared allele-specific effects (homogeneous mode), as well as a joint setting that combines information across modes.

However, analyzing scRNA-seq data for ASE is a multi-faceted computational challenge, and very few existing tools allow for this kind of analysis [18]. The process involves numerous steps: demultiplexing, quality control, read alignment and deduplication, variant calling, allelic read counting, and finally, statistical modeling to pinpoint the single-nucleotide variants with the significant allelic imbalance. Each step requires specific software tools with complex dependencies, orchestrating such a workflow with custom scripts is fragile, difficult to parallelize, and notoriously hard to reproduce, creating a significant barrier to reliable discovery. Workflow management systems such as Nextflow [19] have emerged to solve these problems and gained popularity. Nextflow enables the creation of portable, scalable, and reproducible pipelines by abstracting away complex job scheduling, managing software dependencies through containers (Docker [20], Singularity [21]), and providing built-in mechanisms for logging and resuming failed runs.

Here, we present NEXT-scASV (Nextflow pipeline for Allele-Specific Variant calling), a comprehensive and robust workflow designed specifically for discovery of allele-specific variants from 5′ scRNA-seq data. NEXT-scASV integrates established tools for alignment (HISAT2 [22]), reads deduplication (umi_tools [23]), variant calling (bcftools mpileup [24]), reference bias correction (WASP [25]), and advanced statistical modeling (MIXALIME [26]). We demonstrate its performance on a large dataset of human PBMCs. The high sensitivity afforded by cell type-specific analysis and the direct capture of 5' ends opens the possibility

of investigating allele-specific regulation in rare cell populations or challenging genomic contexts, such as in long non-coding RNAs (lncRNAs) and other lowly expressed or highly tissue-specific genes, which are often underpowered in bulk tissue eQTL studies. By providing a standardized, containerized, and parallelized solution, NEXT-scASV lowers the barrier for performing sophisticated allele-specific analyses in large-scale single-cell studies.

MATERIALS AND METHODS

Pipeline Design and Implementation

NEXT-scASV is implemented in Nextflow (DSL2) with a modular structure where each processing stage (or subflow) is defined as a separate, reusable module. The pipeline execution is managed through a central configuration file, defining input data paths, sample metadata, and important parameters for each module. All software dependencies are packaged into Docker, supporting reproducibility across different computing environments.

The pipeline requires two primary inputs from the user:

1. A metadata JSON file linking sample identifiers to the paths of the corresponding FASTQ files.
2. A barcode-to-group assignment CSV file containing columns for *barcode*, *sample*, and *group* (e.g., a cell type). This file is essential for splitting the data and performing group-level analyses.

The workflow consists of five main subflows, illustrated in Figure 1: (i) Data Splitting, (ii) Alignment and Filtering, (iii) Variant Calling, (iv) Allelic Read Counting, and (v) ASV Calling.

Splitting Subflow

The initial step employs a custom Python script to split the raw FASTQ files based on the provided barcode-group assignments. Each input sample (e.g., one donor) is split into multiple FASTQ files, one for each assigned group (e.g., a cell type). This strategy, while increasing the number of subsequent tasks, is the cornerstone of the pipeline's parallelization, allowing downstream steps to process each sample × group combination independently and in parallel.

Alignment Subflow

Each split FASTQ file is then processed individually. First, the reads are trimmed to remove adapters and low-quality bases using cutadapt v5 [27]. Processed reads are then aligned to the reference genome (e.g., GRCh38) using HISAT2, which efficiently handles splice-aware alignment of RNA-seq data.

We selected HISAT2 over STAR due to its superior speed, a critical factor when processing large single-cell datasets with multiple donors and cell types. This choice does not compromise accuracy: on a dataset with a natural human polymorphism rate of 0.001,

both aligners delivered nearly identical performance, achieving near 100% precision and over 95% recall [28].

Following alignment, PCR duplicates are marked and removed using umi_tools dedup [23] to mitigate technical artifacts. The resulting BAM files are rigorously filtered to retain only high-quality alignments, for our testing we set the following values for the user defining parameters: mapping quality (MAPQ) ≥ 10, a maximum of 2 mismatches, and an insert size ≤ 750 bp.

Variant Calling Subflow

To call heterozygous single-nucleotide variants (SNVs), as in the default MIXALIME workflow [26], the pipeline processes the filtered BAM files from each sample × group combination using bcftools mpileup and bcftools call [24]. Variant calling is performed independently for each chromosome (1-22) to maximize parallelization. The initial call set is stringently filtered to ensure high-confidence heterozygous sites: read depth (DP) ≥ 10, genotype quality (GQ) ≥ 50, and a minimum of 5 reads supporting each allele (AD ≥ 5). At the vcf splitting step, jointly called variants are splitted by samples and each sample vcf file is also filtered by minimum 5 reads supporting each allele.

The choice of variant caller determines the initial set of candidate SNPs and therefore establishes the upper bound for sensitivity and accuracy in downstream analysis. However, in our pipeline, the impact of the caller is mitigated through several subsequent steps. We primarily use the caller for initial SNP discovery, after which we apply rigorous variant-level filters, including genotype quality (GQ), read depth (DP), and allelic depth (AD). Additionally, we implement read-level filters before counting, such as mapping quality (MAPQ), edit distance (NM), and exclusion of QC-failed reads using filter_reads.py and samtools (flag 512). Crucially, the final allele-specific expression (ASE) signal is derived through direct allele-specific read counting from BAM files via count_tags_pileup.py, rather than relying on caller-derived quality scores. Furthermore, variants are called at the patient level, which leverages information across related samples to address the sparse coverage typical of single-cell RNA-seq data. As a result, the final ASE estimates are substantially less dependent on the specific variant caller than in workflows where caller outputs directly determine allelic estimates. For this study, we selected bcftools as our variant caller, as it offers a simpler, faster, and more computationally lightweight solution while still providing robust candidate variants suitable for downstream ASE analysis.

Allelic Read Counting Subflow

A critical challenge in ASV analysis is reference allele mapping bias, where reads containing alternative alleles map with lower confidence. To correct this, the pipeline integrates the WASP tool [25]. For each potential variant site, WASP identifies reads overlapping the site, swaps the alleles, remaps these modified reads, and filters out the original reads that fail to remap correctly. This process generates a corrected set of alignments devoid of reference bias. A Python script [29] then parses these corrected BAM files to count the number of reads supporting the reference and alternative alleles for every

variant in every sample × group combination, producing an extensive table of allelic read counts for statistical testing.

ASV Calling Subflow

The final step identifies the sites with the significant allelic imbalance using the MIXALIME framework [26]. MIXALIME fits probabilistic models to the allelic read counts, accounting for data sparsity and over-dispersion inherent in scRNA-seq. We employed the Beta-Negative Binomial (BetaNB) model, which is the most conservative but reliable for modeling over-dispersed count data. The tool fits model parameters and calculates P-values for the deviation from the expected balanced allelic read counts (i.e., 0.5 ratio). P-values are then aggregated with the Mudholkar-George *logitp* method [30] across hierarchical groups (e.g., within a cell type lineage) to increase statistical power. False discovery rate (FDR) correction is applied (Benjamini-Hochberg), and the significant ASVs are called at, FDR < 0.05. MIXALIME also generates extensive quality control plots, including goodness-of-fit metrics like RMSEA (Root Mean Square Error of Approximation) [31]. Significant ASVs are functionally annotated by overlapping their genomic coordinates with regulatory databases such as GTEx for eQTLs and ADASTRA for allele-specific binding events [2,32], providing immediate biological context and an additional layer of validation against known regulatory single-nucleotide polymorphisms (rSNPs).

Benchmarking Dataset

The pipeline was tested on data from the Asian Immune Diversity Atlas (AIDA) [33], a comprehensive resource of scRNA-seq data from PBMCs of 619 healthy donors. A random subset of 57 donors (23 male, 34 female, aged 25-40) of South Korean ancestry was selected for this benchmarking study to balance computational feasibility with statistical power. The dataset comprised 17 major immune cell types, as annotated in the original paper.

Computational Resource Profiling

The pipeline was executed on a high-performance computing cluster managed by the SLURM workload manager, with a constrained limit of 1 node with 100 threads and 15 TB of RAM. Resource usage (CPU time, peak memory, disk I/O) for each process was meticulously recorded using Nextflow's built-in tracing capabilities. The total execution time was calculated under three scenarios: the sum of all process times (emulating a single CPU, lower bound), the duration of the longest process chain (emulating infinite parallelization, upper bound), and the actual wall time under the 100 threads constraint.

Biological Validation against eQTL Data

To assess the biological relevance of the discovered ASVs, we compared our results against the eQTL analysis published in the original AIDA study. For each significant ASV, we identified its nearest transcription start site (TSS). The resulting list of ASV-associated genes was intersected with the list of genes identified as significantly associated with eQTLs (eGenes) in the AIDA cohort (FDR < 0.05).

Allele-specific analysis with scDALI and DAESC

We ran scDALI and DAESC in two input modes. First, we used a pseudobulk setup consistent with our pipeline, where allelic counts were aggregated for each sample × group combination and used as input for the models. Second, we used a true single-cell setup: at the allelic counting stage we computed reference and alternative counts for each barcode, yielding allele-count matrices for approximately 70,000 cells, which were then used as input for scDALI and DAESC.

For scDALI, we applied additional filtering thresholds: minimum SNPs per cell = 50, minimum cells per SNP = 50, minimum nonzero ALT cells = 5, and minimum nonzero REF cells = 5. scDALI was run in the joint mode (heterozygous + homozygous), which provides a test that is closer in spirit to overall allelic imbalance detection while still leveraging single-cell variation.

For DAESC, we used minimum SNPs per cell = 50 and minimum cells per SNP = 50, and ran the model with the following parameters: num_iter = 50, min_iter = 20, and max_optim = 10.

Variant calling with cellsnp-lite

As an alternative to the default variant-calling branch based on bcftools mpileup/call, we generated variants with cellsnp-lite [34]. In this mode, the pipeline takes the processed, coordinate-sorted and indexed BAM files and runs cellsnp-lite in Mode 2b, using the configured cell barcode/UMI tags (e.g., CB/UB when enabled). cellsnp-lite outputs per-locus reference/alternative allele counts, which are then used as the variant set for the downstream counting/ASE steps.

RESULTS

NEXT-scASV Pipeline Enables Scalable and Reproducible Analysis

The NEXT-scASV pipeline integrates a complex series of tools into a cohesive, automated workflow (Figure 1). Its modular Nextflow DSL2 implementation allows users to easily start, stop, and resume analyses, a critical feature for long-running computations. The use of containers eliminates "dependency hell" and ensures identical results regardless of the host system.

A key design feature is the aggressive data splitting at the outset. While this strategy generates a large number of intermediate files (~15 TB for this study), it unlocks massive parallelization, transforming a computationally prohibitive task into a manageable one. The configuration of the pipeline allows fine-grained control over resource allocation for each step and provides options for cleaning up intermediate files to conserve disk space after successful completion of individual stages.

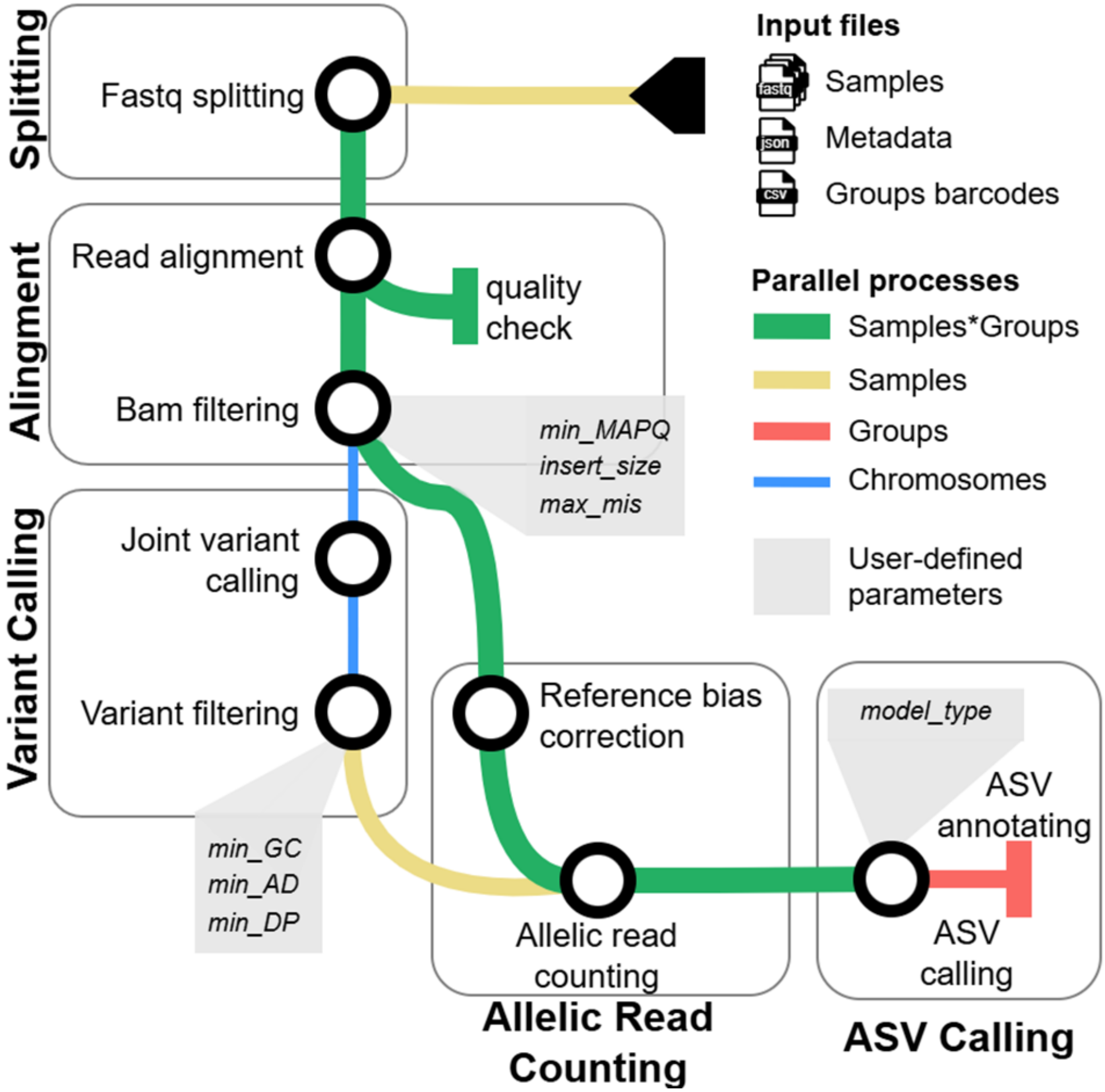

Figure 1. Schematic overview of the NEXT-scASV pipeline. Rounded rectangles represent the five main subflows. Circles represent individual modules within a subflow. The color and thickness of the arrows indicate the data aggregation strategy. Grayscale rectangles highlight important user-configurable parameters.

Pipeline Performance and Resource Utilization

We evaluated the computational cost of processing the 57-donor PBMC dataset. Under a realistic constraint of 100 threads, the pipeline completed in approximately 7 days (Figure 2B). Profiling the resource usage revealed distinct patterns for different stages (Figure 3). The alignment stages ("fastq aligning" and "reference bias correction") were the

most time-consuming but scaled efficiently with the number of available CPUs. In contrast, the "Call variants" stage, split into 22 parallel chromosome-level tasks, represents a parallelization bottleneck; its duration is fixed by the longest chromosome job and cannot be reduced by adding more CPUs beyond this point. This stage also exhibited the highest memory demand, with a peak virtual memory usage of ~120 GB per process and intensive disk I/O (reading/writing over 140 GB per process), highlighting the need for substantial memory and fast storage on compute nodes.

Theoretical estimates illustrate the necessity of parallelization: running the workflow sequentially on a single CPU would take over 400 days (Figure 2C), while perfect parallelization could reduce this to under a day (Figure 2A). Our real-world scenario (100 CPUs) strikes a practical balance, demonstrating that NEXT-scASV can process atlas-scale data within a reasonable timeframe on a high-performance computing cluster typically used in data-intensive biomedical research.

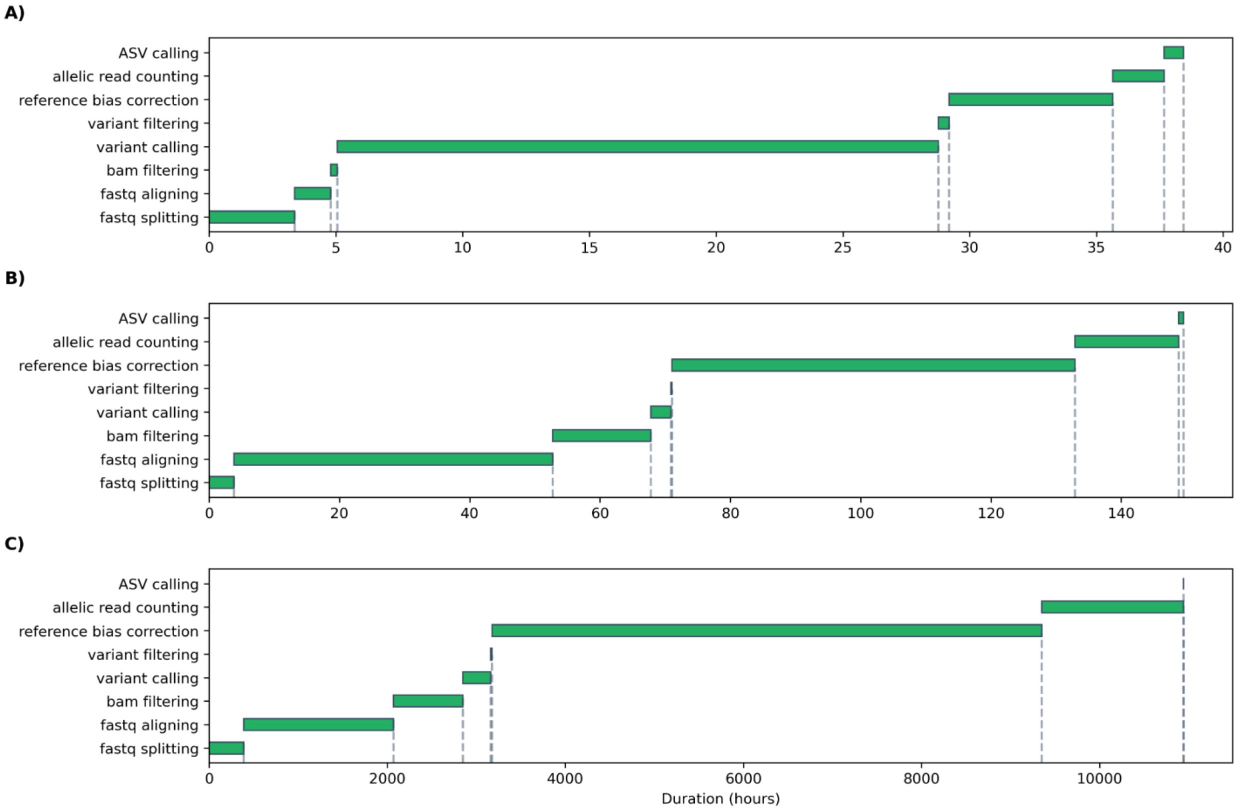

Figure 2. Pipeline execution time under different parallelization scenarios. (A) Mean process duration per stage, representing the ideal upper bound with infinite parallelization. (B) Actual wall-time duration with a limit of 100 concurrent CPUs. (C) Cumulative sum of all process times, representing the lower bound of sequential execution on a single CPU.

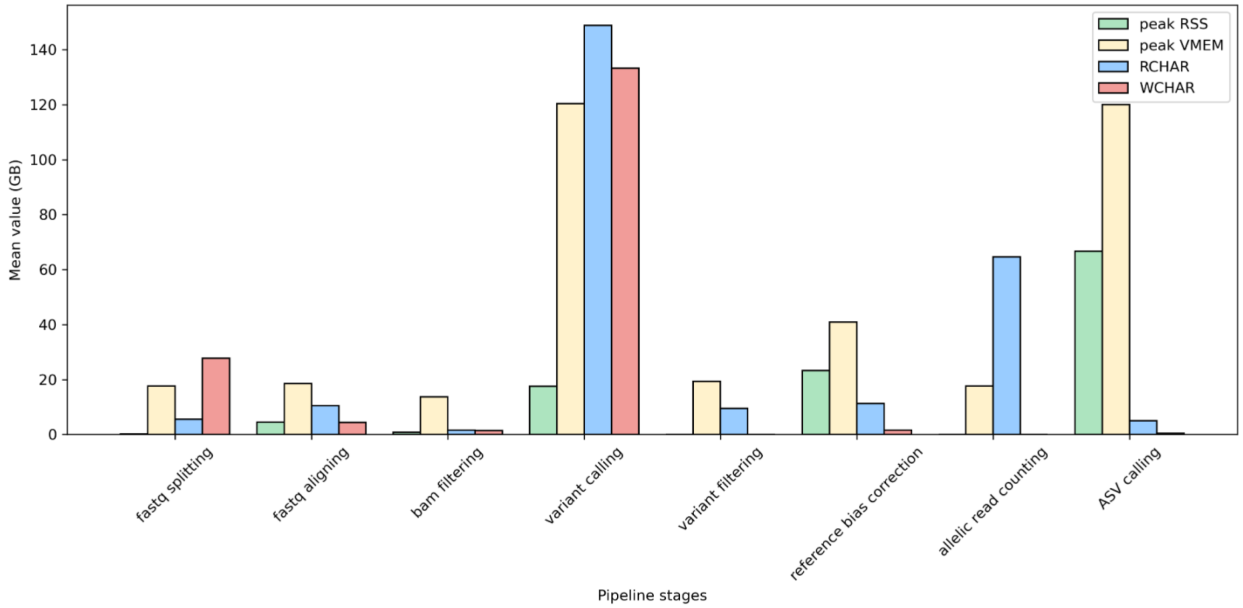

Figure 3: Mean computational resource utilization per process for each major pipeline stage. Metrics shown include: peak RSS (peak Resident Set Size , the maximum amount of physical memory a process has used at any point during its execution), peak VMEM (peak virtual memory usage, includes swap), RCHAR (volume of data read from storage), and WCHAR (volume of data written to storage).

Quality Control and Model Validation

The statistical analysis using MIXALIME passed the basic quality check. Particularly, the goodness-of-fit, measured by RMSEA for all fitted models, was excellent, falling well below the recommended threshold of 0.05 (Supplementary Figure S1A), indicating that the chosen BetaNB model adequately captured the overall distribution of allelic read counts.

As an orthogonal validation, we examined the fraction of discovered ASVs annotated in external databases of rSNPs (GTEx eQTLs and ADASTRA ASBs). We observed that the proportion of annotated variants increased monotonically along with higher thresholds for the FDR significance (Supplementary Figure S1B). This trend is biologically expected—stronger, more reproducible functional variants are more likely to be catalogued in existing databases—and provides independent support for the correctness of our statistical calls.

Caller comparison

To provide an empirical benchmark, we compared bcftools with cellsnp-lite. Cellsnp-lite reported more candidate variants (Figure S3 A), but most additional variants did not pass downstream ASV significance testing in MixALime (Figure S3 B). This suggests that cellsnp-lite is more sensitive to low-coverage variants, but this increased sensitivity does not translate into a clear advantage in terms of final significant ASVs. At the same time, each caller contributed a little fraction of caller-specific variants (Figure S3 B).

ASV tools comparison

We evaluated two alternatives to MixALime: DAESC and scDALI. When we ran them in the pseudobulk mode (the same way as in our pipeline), almost all variants detected by DAESC and scDALI were also detected by MixALime (Fig. S4A). In this setting, MixALime identified many more ASVs. A likely reason is that MixALime is designed to detect allelic imbalance itself, while DAESC and scDALI are primarily designed to test differences between groups or cell states. Next, we ran DAESC and scDALI on true single-cell allele counts. In this case, the number of detected ASVs increased strongly (DAESC: 31 → 188; scDALI: 23 → 130), and unique variants appeared (Fig. S4B). scDALI overlapped with MixALime more than DAESC, consistent with the idea that scDALI in the joint (homozygous + heterozygous) mode is conceptually closer to MixALime. DAESC targets allele-specific effects that vary across cell states or conditions rather than overall allelic imbalance from MixALime, so it should be considered complementary to MixALime rather than a direct alternative. Finally, ASVs reported by all three tools showed substantial overlap with known regulatory variant resources, including GTEx cis-eQTLs and ADASTRA (Fig. S4C–D), indicating that each method recovers a meaningful fraction of previously reported regulatory variants. scDALI and DAESC application details are in Methods: Allele-specific analysis with scDALI and DAESC.

NEXT-scASV Uncovers Allele-Specific Events in Rare Cell Populations and Challenging Genomic Conditions

A major advantage of single-cell resolution is the ability to probe allele-specific regulation in rare cell types. We leveraged MIXALIME's ability to aggregate p-values across different hierarchical levels (e.g., within T-cell subsets before aggregating to all T cells) to enhance statistical power at different levels of resolution. As expected, aggregating data to broader cell types (e.g., all T cells, all PBMCs) increased the total number of detectable ASVs due to greater statistical power from larger read counts (Supplementary Figure S2).

The sensitivity gains from analyzing particular cell types extend beyond discovering effects in rare populations to also detecting ASE of genes with low or cell type-specific expression. This includes challenging targets, such as long non-coding RNAs (lncRNAs), which are often expressed at low levels and whose regulation is highly context-dependent. In our case study, we identified significant ASVs impacting the expression of several non-coding RNAs. Fractions of the detected ASV impacting non-coding RNAs are the same as for all genes (Figure 4B). Importantly, we found in total 19 long non-coding RNAs, some of them are well-studied regulators such as P53 Induced Transcript LINC-PINK or NLRP3 inflammasome regulator LINC00989, and also a number of less-studied RNAs (e.g., LINC02723, LINC01220, LINC01871, LINC01679, LINC02273, LINC01619), that may be included in immune cells regulation. The ability to probe allele-specific regulation of such

genes provides a new avenue for understanding the functional impact of genetic variation in the non-coding genome.

High Concordance with Independent eQTL Analysis Validates Biological Relevance

To validate the biological significance of our findings, we compared the set of genes linked to the detected ASVs with those reported as significantly associated with eQTLs (eGenes) in the AIDA cohort study [33]. This comparison showed a very high level of agreement: on average, 82% (± 6% across cell types) of the genes associated with ASVs were also identified as eGenes (Figure 4 and Figure S5). This strong concordance with an orthogonal analysis performed on a larger cohort with genotyping data provides robust evidence that NEXT-scASV reliably identifies biologically genuine cis-regulatory variants.

The remaining ~18% of ASV-linked genes not reported as eGenes likely represent a mix of false positives and, more interestingly, true cell type-specific regulatory events. These could either be effects that are too weak, narrow context-specific, or dependent on cellular environments not captured in the bulk-tissue eQTL analysis, pointing to potential novel biology uncovered by our single-cell approach.

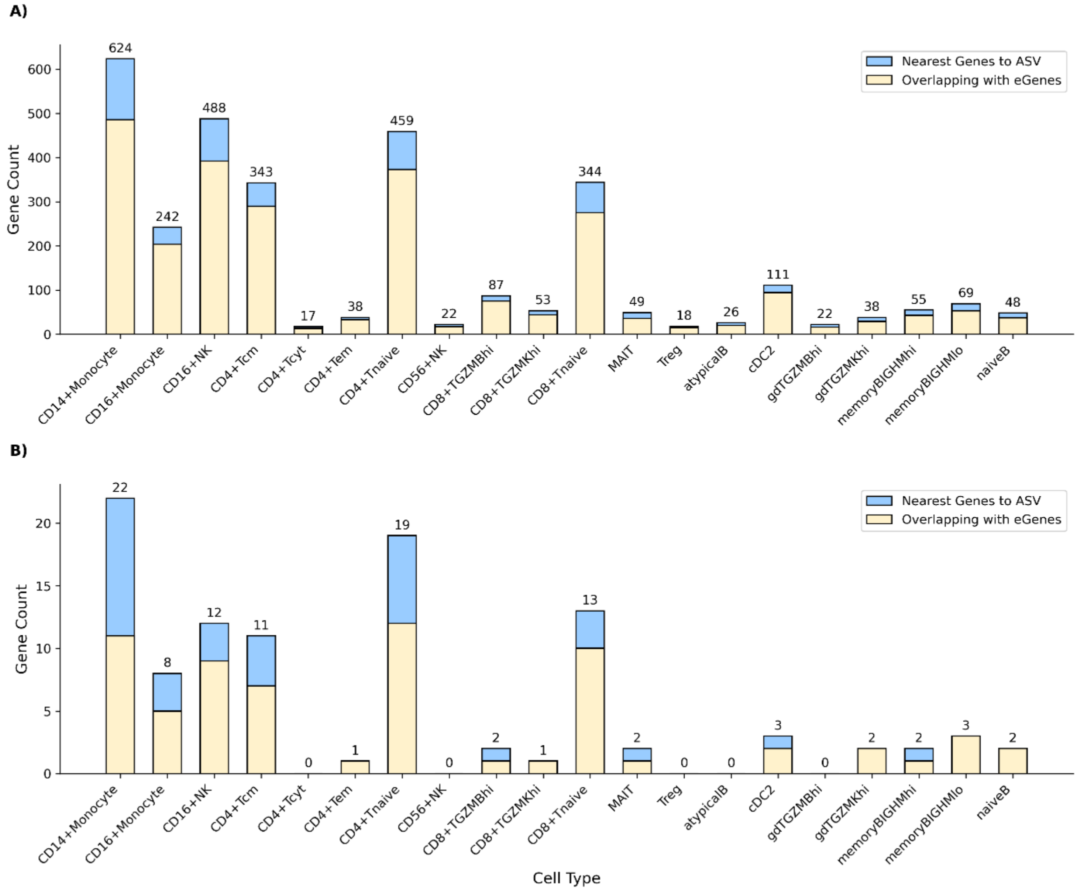

Figure 4. Validation of discovered ASVs by comparison against eQTLs analysis. For each cell type, the bar shows the total number of genes associated with significant ASVs (blue). The

yellow segment indicates the subset of these genes that were also reported as eGenes (FDR < 0.05) in the original cohort eQTL study. A. All genes, B. Non-coding RNAs.

DISCUSSION

We have developed NEXT-scASV, a scalable and reproducible pipeline for the detection of allele-specific variants from 5′ scRNA-seq data. Its containerized implementation ensures that the ASV calling is reproducible across different computing platforms, a cornerstone of robust scientific practice. Our benchmarking on data from 57 individuals (135,000 cells) demonstrates that NEXT-scASV is capable of processing large datasets in a feasible timeframe on a mid-sized compute cluster. The resource analysis provides a valuable guide for other groups planning similar studies. The pipeline performance is not just technical: its results agree well with existing rSNP annotations showing 80% concordance with established eQTLs, while also revealing cell type-specific effects that are invisible to bulk approaches.

A key design philosophy was to enable *de novo* discovery without requiring external genotyping data, making the pipeline widely applicable to standard 5' scRNA-seq study designs. The integration of WASP mitigates reference mapping bias, a known source of false positives in ASE studies, and the use of MIXALIME provides a statistically rigorous framework capable of handling the overdispersion in allelic counts arising due to noise and sparsity of single-cell data.

The ability of NEXT-scASV to detect signals in rare cell types like gdT GZMBhi cells is particularly exciting. It opens the door to investigating allele-specific regulation in rare cell populations involved in disease, development, and immune response, which have been largely inaccessible. An exciting implication of our approach is its potential to illuminate allele-specific regulation in genomic regions that have been historically difficult to study. The combination of cell type-specific resolution and sensitivity makes NEXT-scASV particularly suited to investigate ASE of lncRNAs and other lowly expressed genes. These categories are often poorly tagged in bulk tissue eQTL studies due to their low expression and high cell type specificity. By isolating the relevant cell type, our pipeline can overcome this limitation, offering a powerful strategy to ascribe function to genetic variants associated with lncRNAs and other elusive elements of the regulome.

The modular, DSL2-based architecture of Nextflow is a core strength of NEXT-scASV, ensuring it is not a static tool but a flexible framework for future methodological developments. The pipeline can be readily adapted to other single-cell modalities that probe cis-regulatory activity, such as scATAC-seq for identifying allele-specific accessibility or multiome assays that simultaneously measure chromatin accessibility and gene expression. This would primarily involve swapping the alignment and initial processing modules while leveraging the same robust downstream variant calling and statistical analysis framework. Furthermore, the modular design simplifies the process of incorporating new best practices, such as advanced filters for technical artifacts or novel statistical models for allelic imbalance, ensuring the pipeline remains at the forefront of the field without requiring a

complete rebuild. This adaptability makes NEXT-scASV a lasting resource for the community, capable of evolving alongside rapidly advancing single-cell technologies.

We compared the key step of our workflow—detecting allele-specific events with MixALime—to methods with closely related functionality (scDALI and DAESC). Results suggest that scDALI provides a conservative subset of MixALime calls, while DAESC can expand the analysis by highlighting variants with context-dependent allelic effects.

While NEXT-scASV provides a robust framework for allele-specific analysis, several limitations should be mentioned. First, the pipeline's current implementation requires a predefined cell type annotation provided by the user via a barcode-group assignment file. It is therefore dependent on the accuracy and resolution of this external annotation. While the hierarchical aggregation in MIXALIME mitigates this to some degree, integrating the pipeline with a more advanced probabilistic cell typing could be a valuable future direction.

Second, the computational footprint, particularly the storage requirements for intermediate files (~15 TB for this study) and the high I/O load during the variant calling stage, can be prohibitive for extra large studies without access to high-performance computing infrastructure with fast and capacious storage.

Third, the variant calling step relies solely on the scRNA-seq data itself. While this de novo approach is a key feature that increases broad applicability, it may be less sensitive than methods that incorporate external genotype information from array data or whole-genome sequencing. Finally, the statistical power to detect ASVs is inherently constrained by the number of expressed reads covering a heterozygous SNP in a given cell type. While aggregation of related cell types helps, very rare cell types or extremely lowly expressed genes will remain challenging to analyze, a limitation inherent to all current scRNA-seq analyses.

In conclusion, NEXT-scASV provides the community with a validated, scalable, and reproducible solution for deciphering allele-specific regulation from 5′ scRNA-seq data. By transforming a complex, multi-stage analysis into an automated and portable workflow, it empowers researchers to move beyond logistical challenges and focus on biological discovery. We demonstrate that it reliably recovers known regulatory biology while uniquely enabling the exploration of genetic effects in rare cell populations and understudied genomic elements. As single-cell atlas projects continue to expand, NEXT-scASV stands as a critical tool for unlocking the functional impact of genetic variation across the full spectrum of cellular diversity in health and disease.

AVAILABILITY OF SOURCE CODE AND REQUIREMENTS

Project name: NEXT-scASV
Project homepage: https://github.com/MedvedevaLab/NEXT-scASV
License: MIT license
Operating system(s): Linux (CentOS/Ubuntu)

Package management: Conda
Programming language: Nextflow, Python, Bash
Hardware requirements: High-performance computing (HPC) cluster recommended. . Requirements depend on input data size. Tested on SLURM cluster with 100 CPUs and 100 GB RAM. Minimum 16 GB RAM for small test datasets.
RRID: SCR_027470

DATA AVAILABILITY

We used the data from the AIDA datasets and it is available via the HCA Data Portal [35]. The supporting data is deposited in GigaDB [36].

AUTHOR CONTRIBUTIONS

Conceptualization: I.K., Y.M., A.Sh. Code curation: A.Sh., A.B., V.N., G.M. Data curation and analysis: A.Sh., P.A. Supervision: I.K., Y.M. Writing—original draft: A.Sh., Y.M. Writing—review and editing: A.Sh., Y.M., I.K., A.B., A.St., P.A.

FUNDING

Pipeline creation, testing, application to real data and analysis of the results has been supported by the RSF 23-14-00371 grant to Y.A.M. MIXALIME adaptation was supported by MSHERF grant number № 075-15-2025-014 (previously № 075-15-2024-666). ADASTRA ASB analysis was supported by assignment FFRW-2025-010.

COMPETING INTERESTS

We declare that we do not have any competing interests.

SUPPLEMENTARY DATA

Packages versions: python=3.10.0; Cutadapt=5.0; HISAT2=2.2.1; UMI-tools=1.1.6; Bcftools=1.21; WASP=v0.4.3 ; MIXALIME==2.27.3

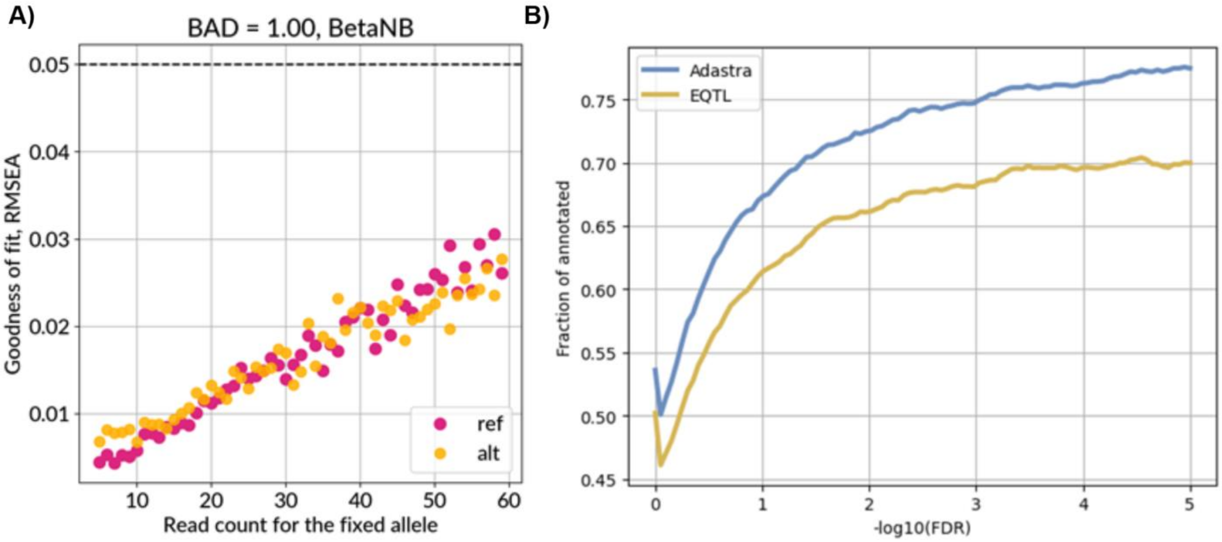

Figure S1: Quality control of the ASV detection. (A) Root Mean Square Error of Approximation (RMSEA) for all fitted MIXALIME models. The dashed line indicates the desired threshold of 0.05. (B) Fraction of significant ASVs that can be annotated in the GTEx (eQTL) and ADASTRA (ASB) databases as a function of the FDR threshold.

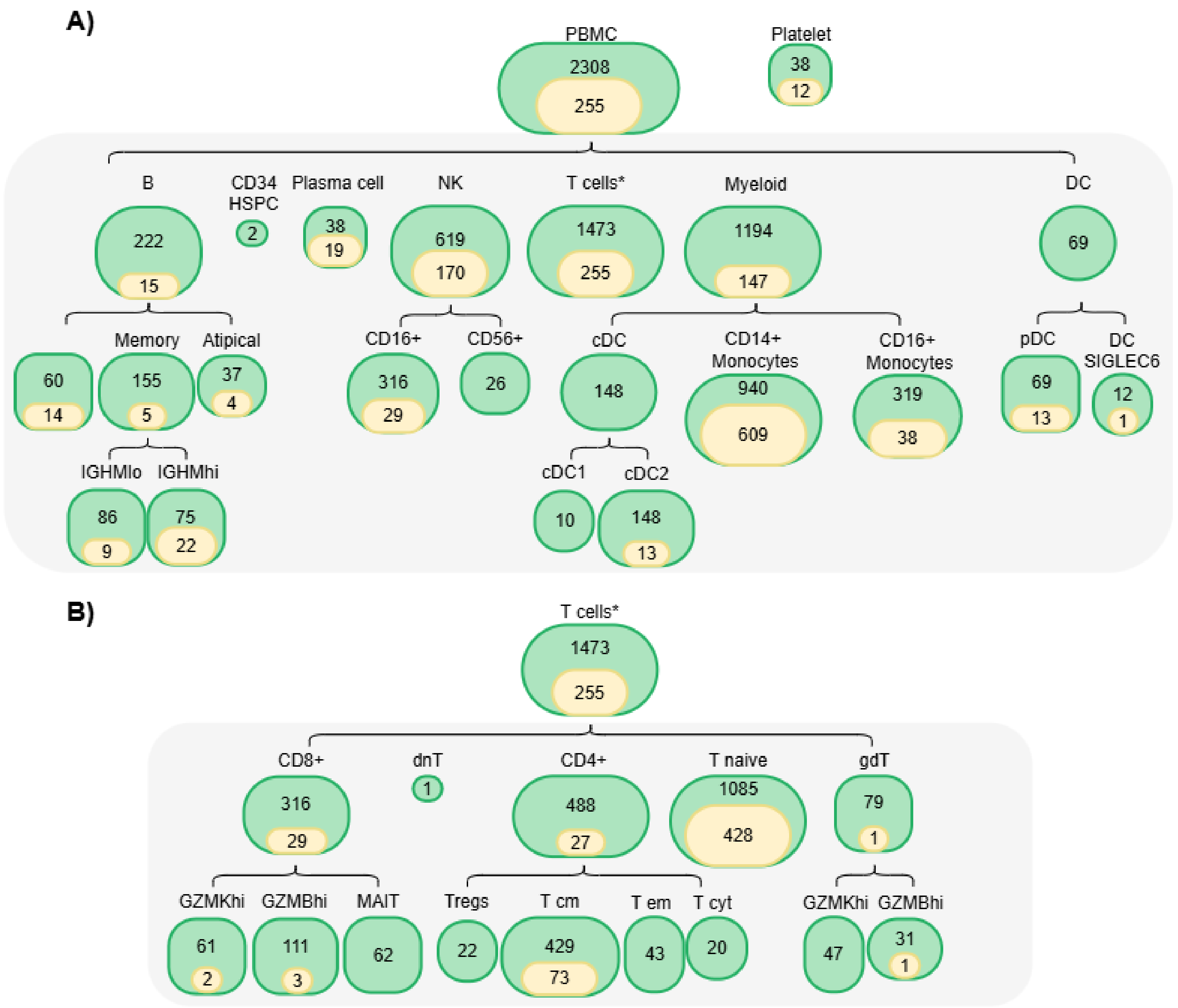

Figure S2: Number of significant ASVs detected at different levels of hierarchical cell type aggregation. The number in the green shape defines the number of found ASV, the yellow shape includes the number of unique found ASV per cell type (counts represent events unique to a node after subtracting all events from its parent nodes, so the nodes show only events not classified into any sub-type, highlighting the gain from broader aggregation). (A). Main PBMC hierarchy without T cells branching. B. T cells branching.

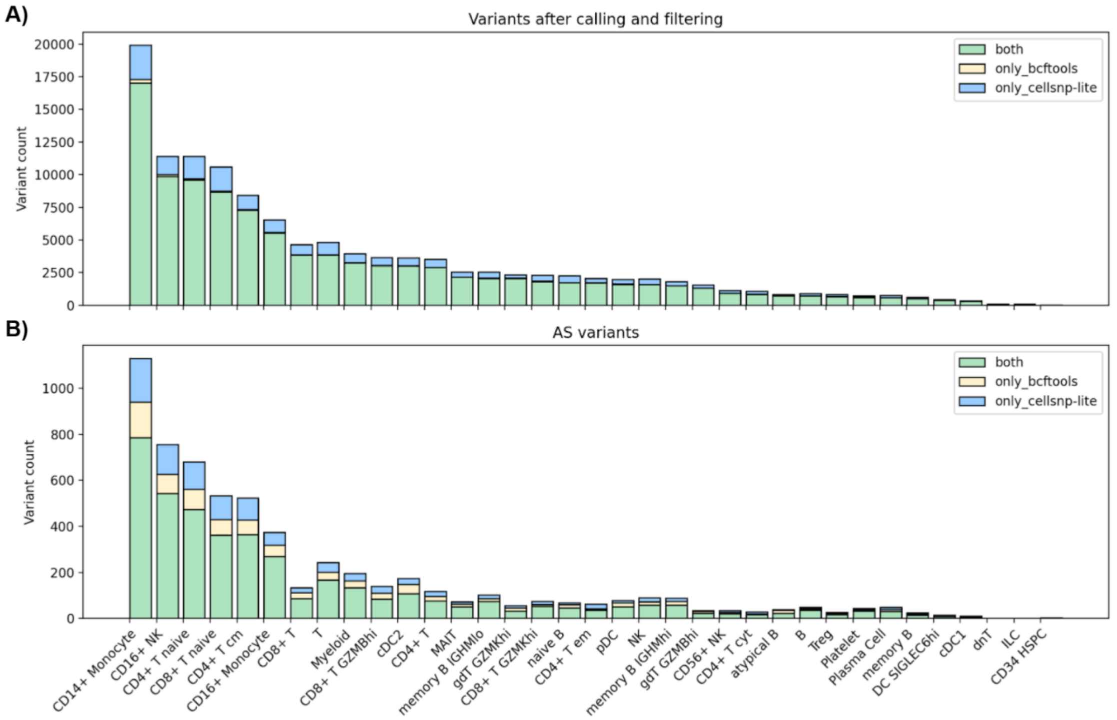

Figure S3: Number of variants that callers can detect. (A) Barplot with variants counts after calling and filtering, with green bar refers to both callers detections, yellow bar with unique bcftools mpileup variants counts and blue bars with only cellsnp-lite detected variants. (B) Significant ASV after each caller.

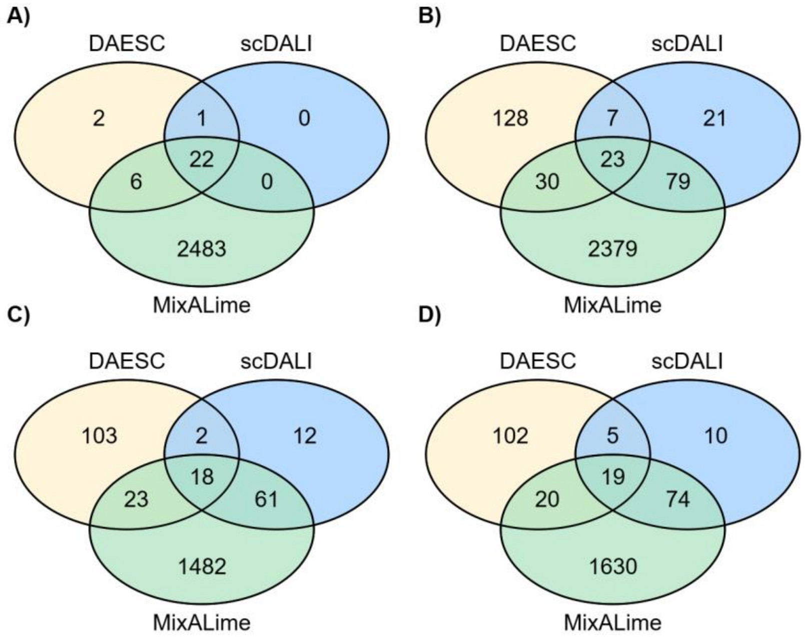

Figure S4: Intersections of significant ASV (FDR ≤ 0.05) identified by DAESC, scDALI, and MixALime. (A) All three tools use pseudobulk allelic counts (sample × cell group) as input. (B) DAESC and scDALI use single-cell allelic counts as input; MixALime uses pseudobulk counts. (C) Subset of significant ASV from B that overlap with cis-eQTLs in the GTEx database. (D) Subset of significant ASVs from B that overlap with regulatory variants in the ADASTRA database.

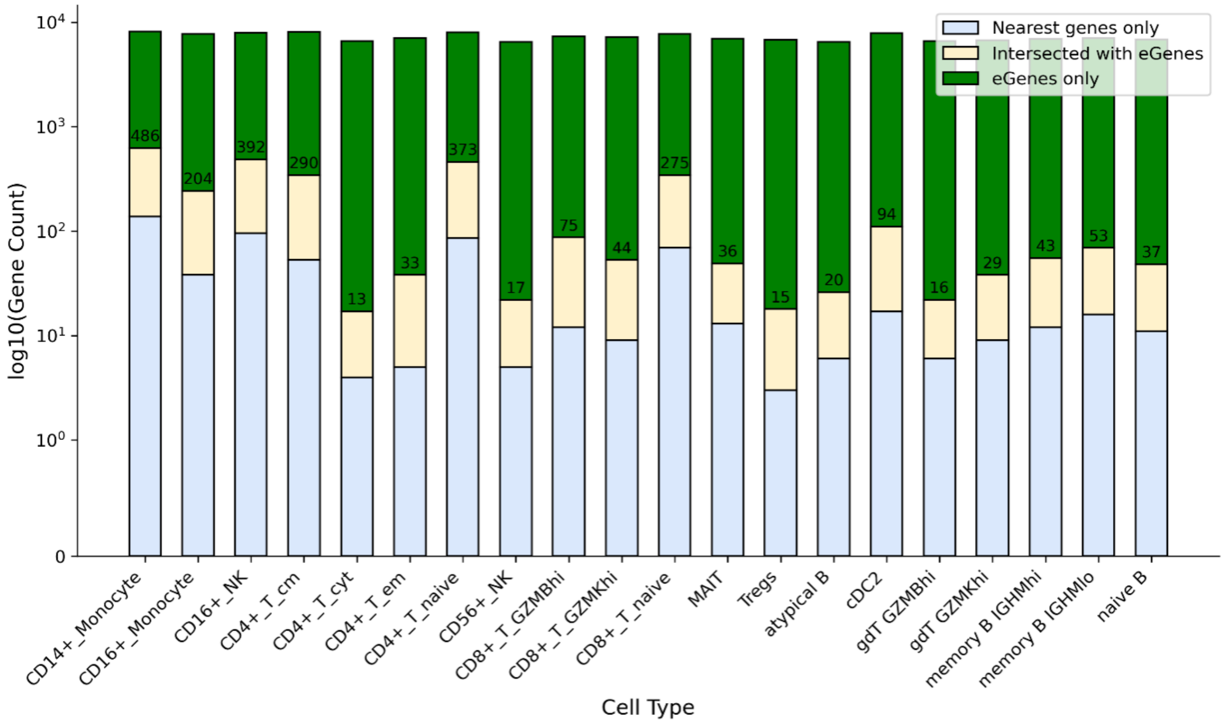

Figure S5: Validation of discovered ASVs by comparison against eQTLs analysis. For each cell type, the bar shows the total number of genes associated with significant ASVs (blue). The yellow segment indicates the subset of these genes that were also reported as eGenes (FDR < 0.05) in the original cohort eQTL study. The green segment represents the number eGens that are not intersected with the found ASVs.

1. Ge B, Pokholok DK, Kwan T, Grundberg E, Morcos L, Verlaan DJ, et al.. Global patterns of cis variation in human cells revealed by high-density allelic expression analysis. *Nature Genetics* (2009). Nature Publishing Group; 41:1216–222009; DOI: https://doi.org/10.1038/ng.473

2. Abramov S, Boytsov A, Bykova D, Penzar DD, Yevshin I, Kolmykov SK, et al.. Landscape of allele-specific transcription factor binding in the human genome. *Nat Commun* (2021). 12:27512021; DOI: https://doi.org/10.1038/s41467-021-23007-0

3. Maurano MT, Haugen E, Sandstrom R, Vierstra J, Shafer A, Kaul R, et al.. Large-scale identification of sequence variants influencing human transcription factor occupancy in vivo. *Nature Genetics* (2015). Nature Publishing Group; 47:1393–4012015; DOI: https://doi.org/10.1038/ng.3432

4. de Santiago I, Liu W, Yuan K, O'Reilly M, Chilamakuri CSR, Ponder BAJ, et al.. BaalChIP: Bayesian analysis of allele-specific transcription factor binding in cancer genomes. *Genome Biology* (2017). BioMed Central; 18:1–172017; DOI: https://doi.org/10.1186/s13059-017-1165-7

5. Chen J, Rozowsky J, Galeev TR, Harmanci A, Kitchen R, Bedford J, et al.. A uniform survey of allele-specific binding and expression over 1000-Genomes-Project individuals. *Nature Communications* (2016). Nature Publishing Group; 7:1–132016; DOI: https://doi.org/10.1038/ncomms11101

6. Miller BR, Morse AM, Borgert JE, Liu Z, Sinclair K, Gamble G, et al.. Testcrosses are an efficient strategy for identifying cis-regulatory variation: Bayesian analysis of allele-specific expression (BayesASE). *G3 Genes|Genomes|Genetics* (2021). Oxford Academic; 11:jkab0962021; DOI: https://doi.org/10.1093/g3journal/jkab096

7. Amoah K, Hsiao Y-HE, Bahn JH, Sun Y, Burghard C, Tan BX, et al.. Allele-specific alternative splicing and its functional genetic variants in human tissues. *Genome Research* (2021). 31:3592021; DOI: https://doi.org/10.1101/gr.265637.120

8. Liu Z, Dong X, Li Y. A Genome-Wide Study of Allele-Specific Expression in Colorectal Cancer. *Front Genet* (2018). Frontiers; 9:4147642018; DOI: https://doi.org/10.3389/fgene.2018.00570

9. Frésard L, Smail C, Ferraro NM, Teran NA, Li X, Smith KS, et al.. Identification of rare-disease genes using blood transcriptome sequencing and large control cohorts. *Nat Med* (2019). 25:911–92019; DOI: https://doi.org/10.1038/s41591-019-0457-8

10. Cummings BB, Marshall JL, Tukiainen T, Lek M, Donkervoort S, Foley AR, et al.. Improving genetic diagnosis in Mendelian disease with transcriptome sequencing. *Sci Transl Med* (2017). DOI: https://doi.org/10.1126/scitranslmed.aal5209.

11. Stachowiak M, Szczerbal I, Flisikowski K. Investigation of allele-specific expression of genes involved in adipogenesis and lipid metabolism suggests complex regulatory mechanisms of PPARGC1A expression in porcine fat tissues. *BMC Genetics* (2018). BioMed Central; 19:1–92018; DOI: https://doi.org/10.1186/s12863-018-0696-6

12. Li Y, Huang Z, Zhang Z, Wang Q, Li F, Wang S, et al.. FIPRESCI: droplet microfluidics based combinatorial indexing for massive-scale 5′-end single-cell RNA sequencing. *Genome Biology* (2023). BioMed Central; 24:1–322023; DOI: https://doi.org/10.1186/s13059-023-02893-1

13. Alam T, Medvedeva YA, Jia H, Brown JB, Lipovich L, Bajic VB. Promoter Analysis Reveals Globally Differential Regulation of Human Long Non-Coding RNA and Protein-Coding Genes. *PLOS ONE* (2014). Public Library of Science; 9:e1094432014; DOI: https://doi.org/10.1371/journal.pone.0109443

14. Choi K, Raghupathy N, Churchill GA. A Bayesian mixture model for the analysis of allelic expression in single cells. *Nature Communications* (2019). Nature Publishing Group; 10:51882019; DOI: https://doi.org/10.1038/s41467-019-13099-0

15. Jiang Y, Zhang NR, Li M. SCALE: modeling allele-specific gene expression by single-cell RNA sequencing. *Genome Biology* (2017). BioMed Central; 18:742017; DOI: https://doi.org/10.1186/s13059-017-1200-8

16. Qi G, Strober BJ, Popp JM, Keener R, Ji H, Battle A. Single-cell allele-specific expression analysis reveals dynamic and cell-type-specific regulatory effects. *Nature Communications* (2023). Nature

Publishing Group; 14:63172023; DOI: https://doi.org/10.1038/s41467-023-42016-9

17. Heinen T, Secchia S, Reddington JP, Zhao B, Furlong EEM, Stegle O. scDALI: modeling allelic heterogeneity in single cells reveals context-specific genetic regulation. *Genome Biology* (2022). 23:82022; DOI: https://doi.org/10.1186/s13059-021-02593-8

18. Mattevi S, Mazzarotto F, Martini P. Allele-specific expression analysis: pipelines, applications, challenges, and unmet needs. *Computers in Biology and Medicine* (2025). Pergamon; 196:1108902025; DOI: https://doi.org/10.1016/j.compbiomed.2025.110890

19. Di Tommaso P, Chatzou M, Floden EW, Barja PP, Palumbo E, Notredame C. Nextflow enables reproducible computational workflows. *Nature Biotechnology* (2017). Nature Publishing Group; 35:316–92017; DOI: https://doi.org/10.1038/nbt.3820

20. Ratliff S: Docker: Accelerated Container Application Development. Docker. Simeon Ratliff; https://www.docker.com/ (2025). Accessed 2025 May 1.

21. Kurtzer GM, Sochat V, Bauer MW. Singularity: Scientific containers for mobility of compute. *PLOS ONE* (2017). Public Library of Science; 12:e01774592017; DOI: https://doi.org/10.1371/journal.pone.0177459

22. Kim D, Paggi JM, Park C, Bennett C, Salzberg SL. Graph-based genome alignment and genotyping with HISAT2 and HISAT-genotype. *Nature Biotechnology* (2019). Nature Publishing Group; 37:907–152019; DOI: https://doi.org/10.1038/s41587-019-0201-4

23. Smith T, Heger A, Sudbery I. UMI-tools: modeling sequencing errors in Unique Molecular Identifiers to improve quantification accuracy. *Genome Res* (2017). 27:491–92017; DOI: https://doi.org/10.1101/gr.209601.116

24. Li H, Handsaker B, Wysoker A, Fennell T, Ruan J, Homer N, et al.. The Sequence Alignment/Map format and SAMtools. *Bioinformatics* (2009). 25:2078–92009; DOI: https://doi.org/10.1093/bioinformatics/btp352

25. van de Geijn B, McVicker G, Gilad Y, Pritchard JK. WASP: allele-specific software for robust molecular quantitative trait locus discovery. *Nature Methods* (2015). Nature Publishing Group; 12:1061–32015; DOI: https://doi.org/10.1038/nmeth.3582

26. Buyan A, Meshcheryakov G, Safronov V, Abramov S, Boytsov A, Nozdrin V, et al.. Statistical framework for calling allelic imbalance in high-throughput sequencing data. *Nature Communications* (2025). Nature Publishing Group; 16:1–192025; DOI: https://doi.org/10.1038/s41467-024-55513-2

27. Martin M. Cutadapt removes adapter sequences from high-throughput sequencing reads. *EMBnet.journal* (2011). 17:10–22011; DOI: https://doi.org/10.14806/ej.17.1.200

28. Baruzzo G, Hayer KE, Kim EJ, Di Camillo B, FitzGerald GA, Grant GR. Simulation-based comprehensive benchmarking of RNA-seq aligners. *Nature Methods* (2017). Nature Publishing Group; 14:135–92016; DOI: https://doi.org/10.1038/nmeth.4106

29. Vierstra J, Lazar J, Sandstrom R, Halow J, Lee K, Bates D, et al.. Global reference mapping of human transcription factor footprints. *Nature* (2020). Nature Publishing Group; 583:729–362020; DOI: https://doi.org/10.1038/s41586-020-2528-x

30. George EO, Mudholkar GS. On the convolution of logistic random variables. *Metrika* (1983). Springer; 30:1–131983; DOI: https://doi.org/10.1007/BF02056895

31. Browne MW, Cudeck R. Alternative Ways of Assessing Model Fit. *Sociological Methods & Research* (1992). SAGE PERIODICALS PRESS; DOI: https://doi.org/10.1177/0049124192021002005.

32. Aguet F, Anand S, Ardlie KG, Gabriel S, Getz GA, Graubert A, et al.. The GTEx Consortium atlas of genetic regulatory effects across human tissues. *Science* (2020). American Association for the Advancement of Science; DOI: https://doi.org/10.1126/science.aaz1776.

33. Kock KH, Tan LM, Han KY, Ando Y, Jevapatarakul D, Chatterjee A, et al.. Asian diversity in human immune cells. *Cell* (2025). Elsevier; 188:2288–306.e242025; DOI: https://doi.org/10.1016/j.cell.2025.02.017

34. Huang X, Huang Y. Cellsnp-lite: an efficient tool for genotyping single cells. *Bioinformatics* (2021). Oxford Academic; 37:4569–712021; DOI: https://doi.org/10.1093/bioinformatics/btab358

35. Human Cell Atlas Project: Single-cell transcriptomic atlas of human tissues. Human Cell Atlas Data Portal. https://data.humancellatlas.org/explore/projects/f0f89c14-7460-4bab-9d42-22228a91f185. Accessed 20 Feb 2026.

36. Shevtsov A; Buyan A; Nozdrin V; Akhtyamov P; Stupnikov A; Meshcheryakov G; Kulakovskiy I V; Medvedeva Y A; (2026): Supporting data for "NEXT-scASV: A Nextflow Pipeline for Allele-Specific Variants Calling from single cell RNA-seq data" GigaScience Database. https://doi.org/10.5524/102816

Click here to access/download;Figure;fig_s1.png

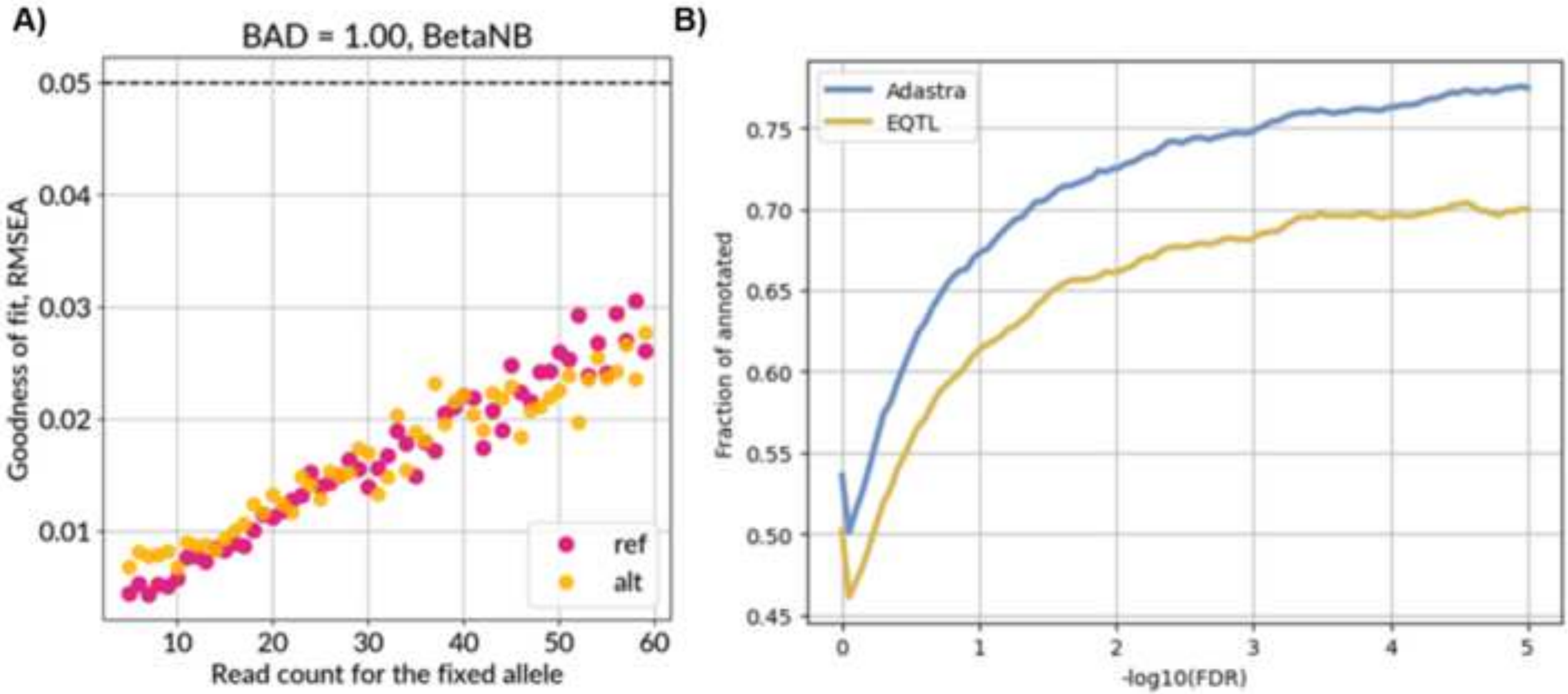

Click here to access/download;Figure;fig_4.png

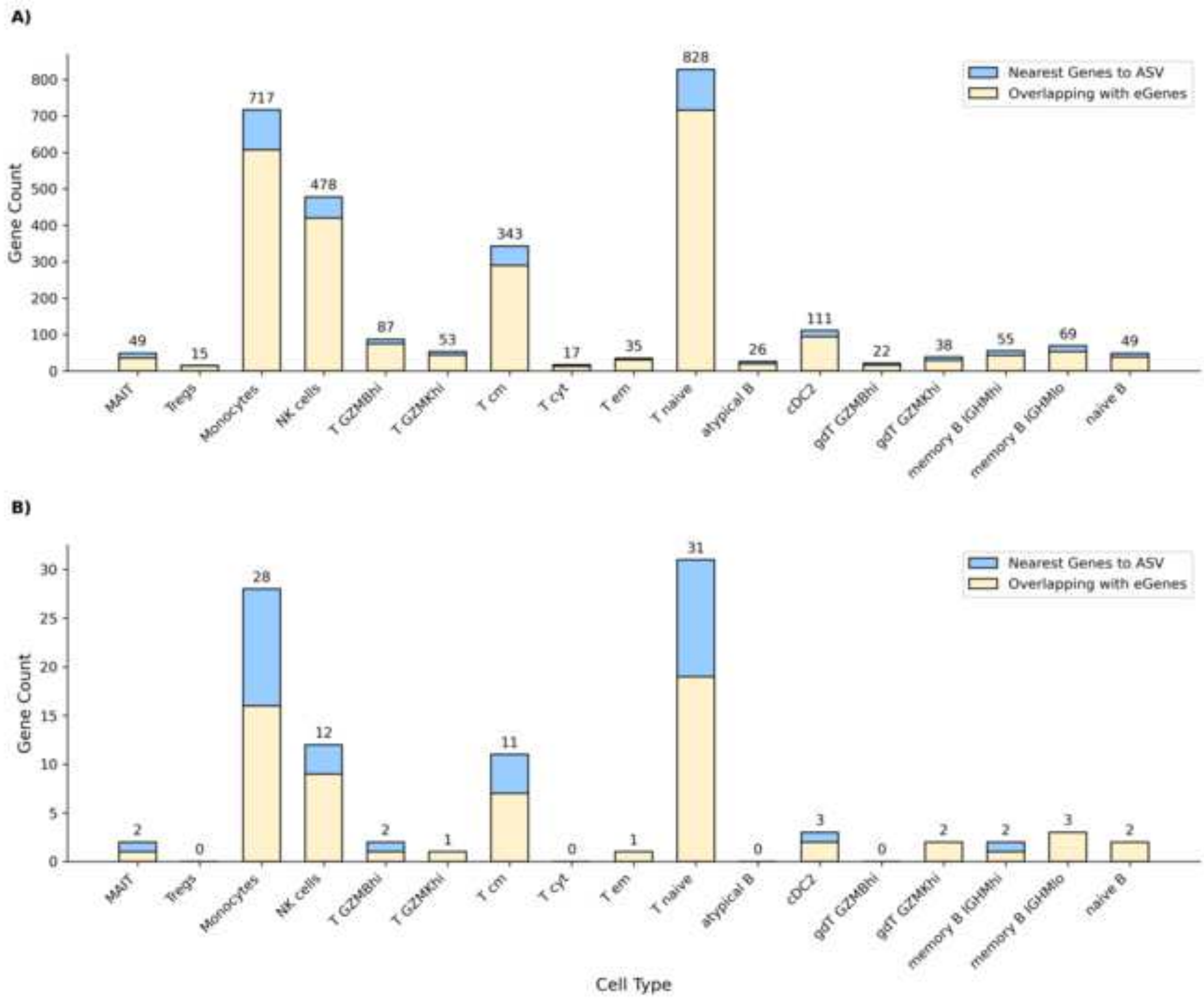

Click here to access/download;Figure;fig_1.png

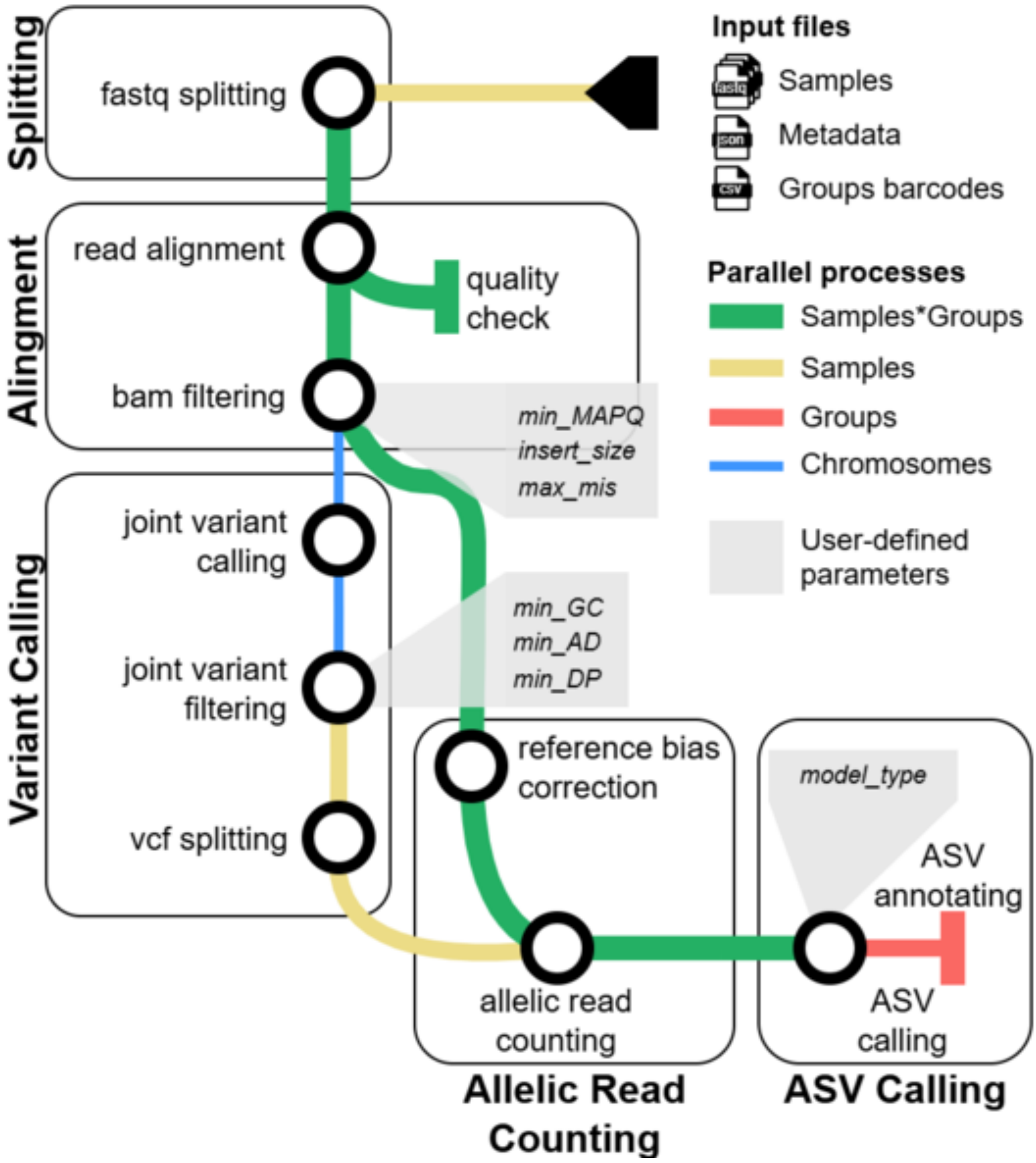

Click here to access/download;Figure;fig_3.png

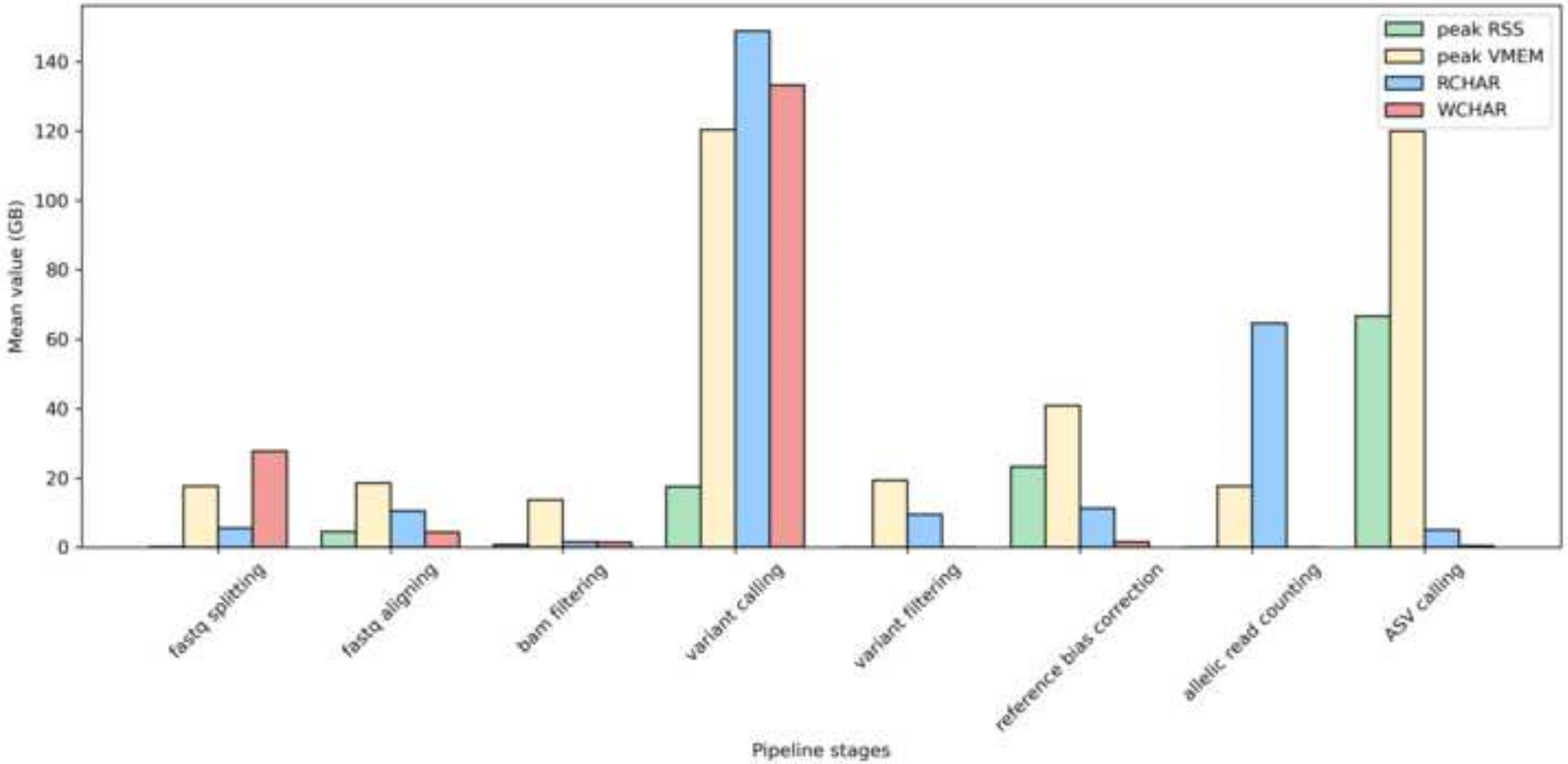

Click here to access/download;Figure;fig_2.png

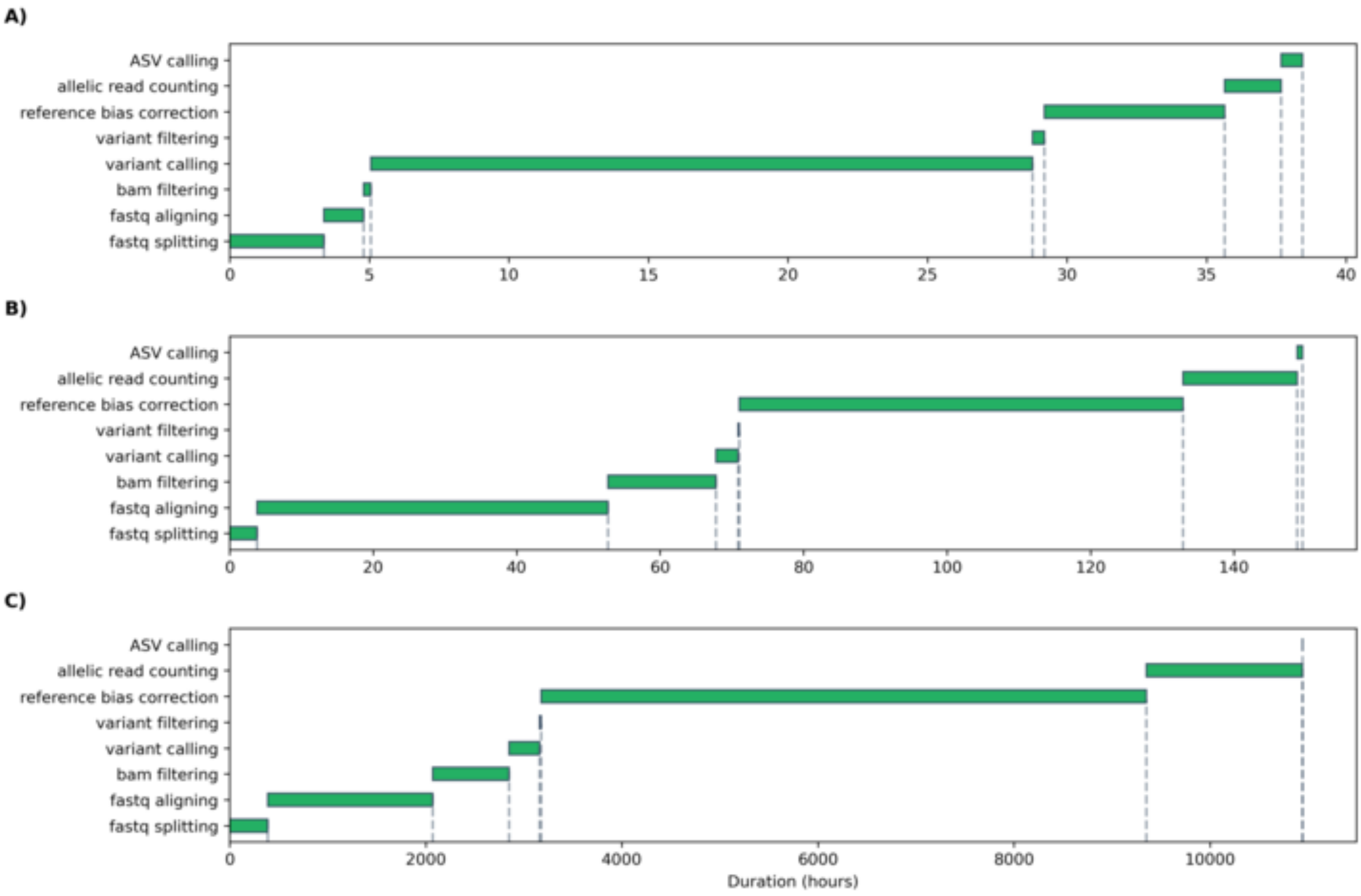

Click here to access/download;Figure;fig_s2.png

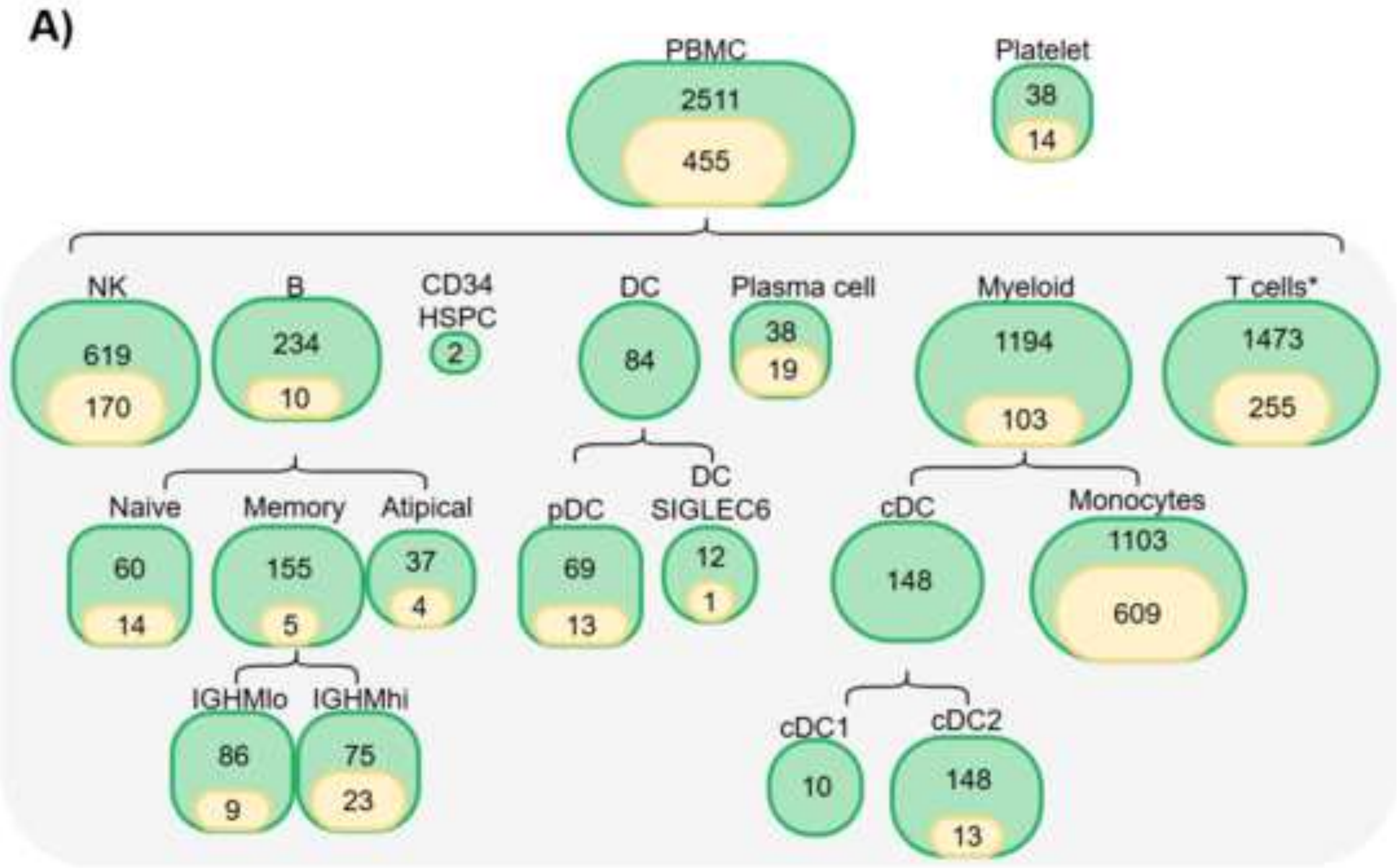

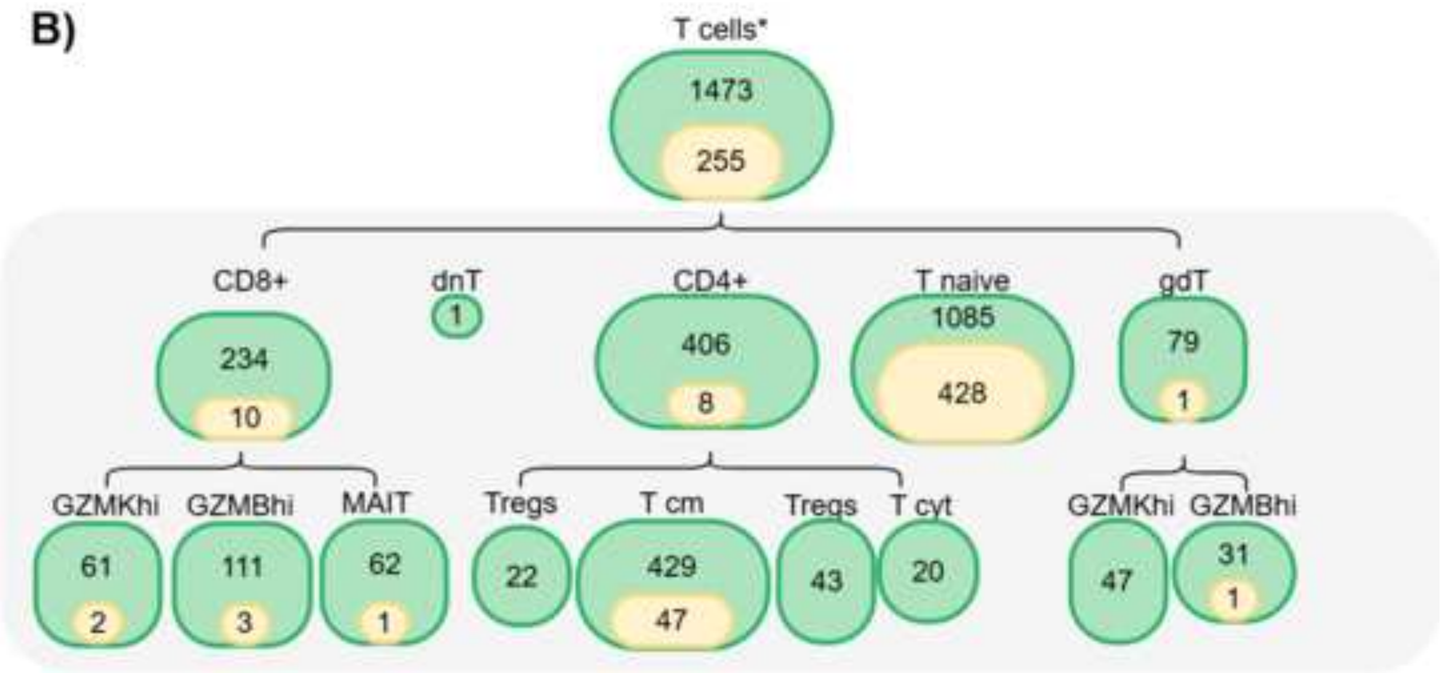

Click here to access/download;Figure;s3_revision.png

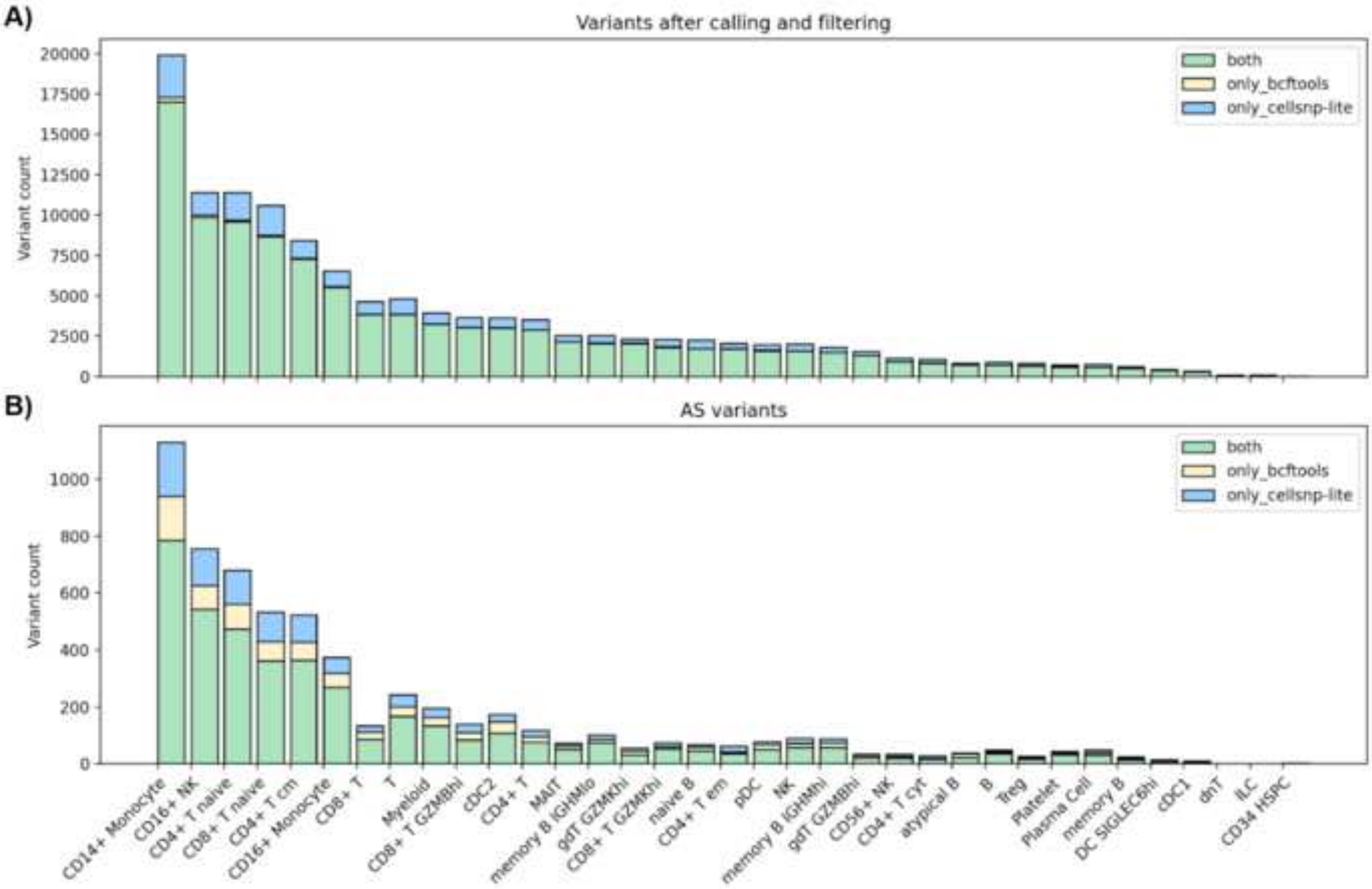

Click here to access/download;Figure;s4_revision.png

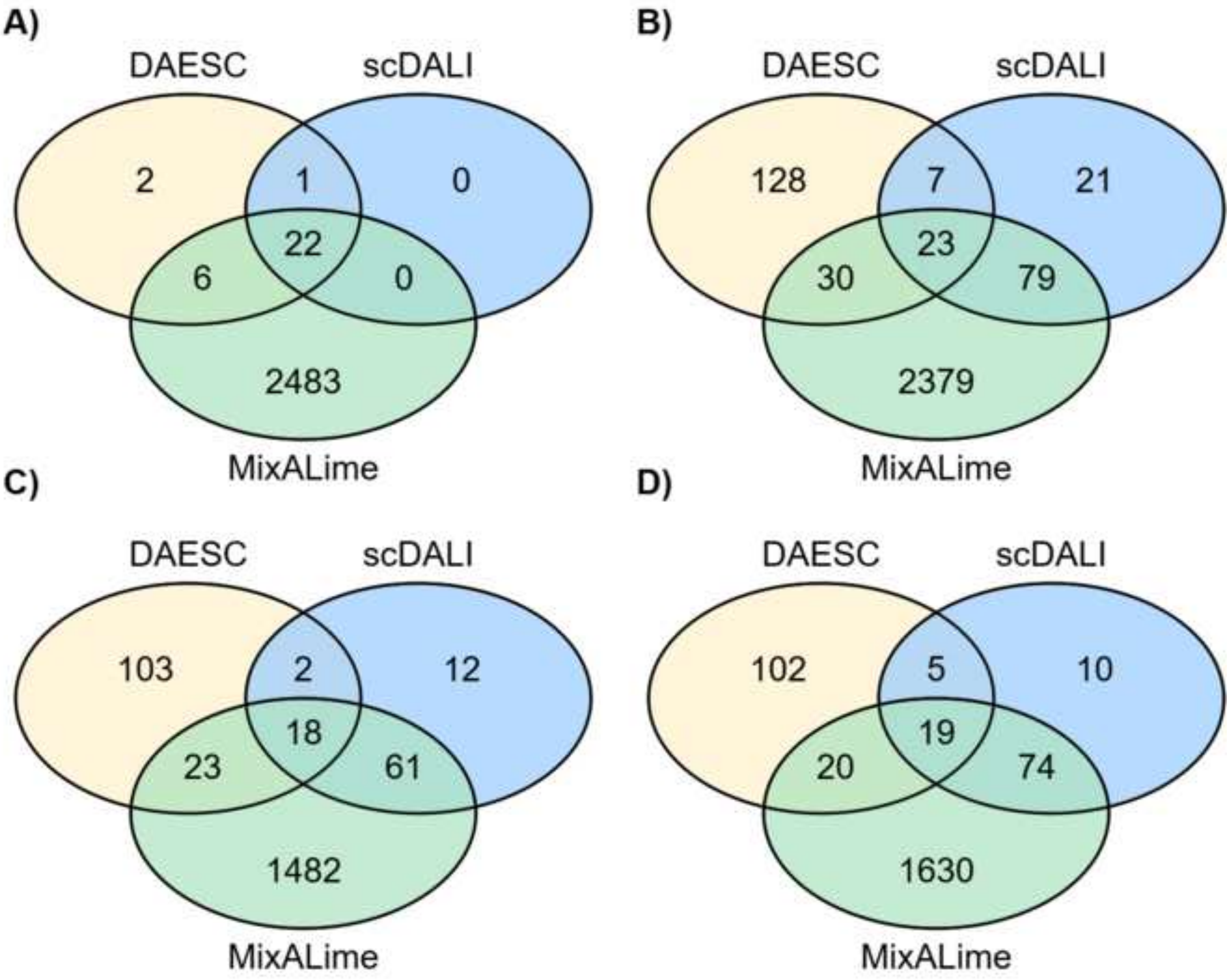

Click here to access/download;Figure;s5_revision.png

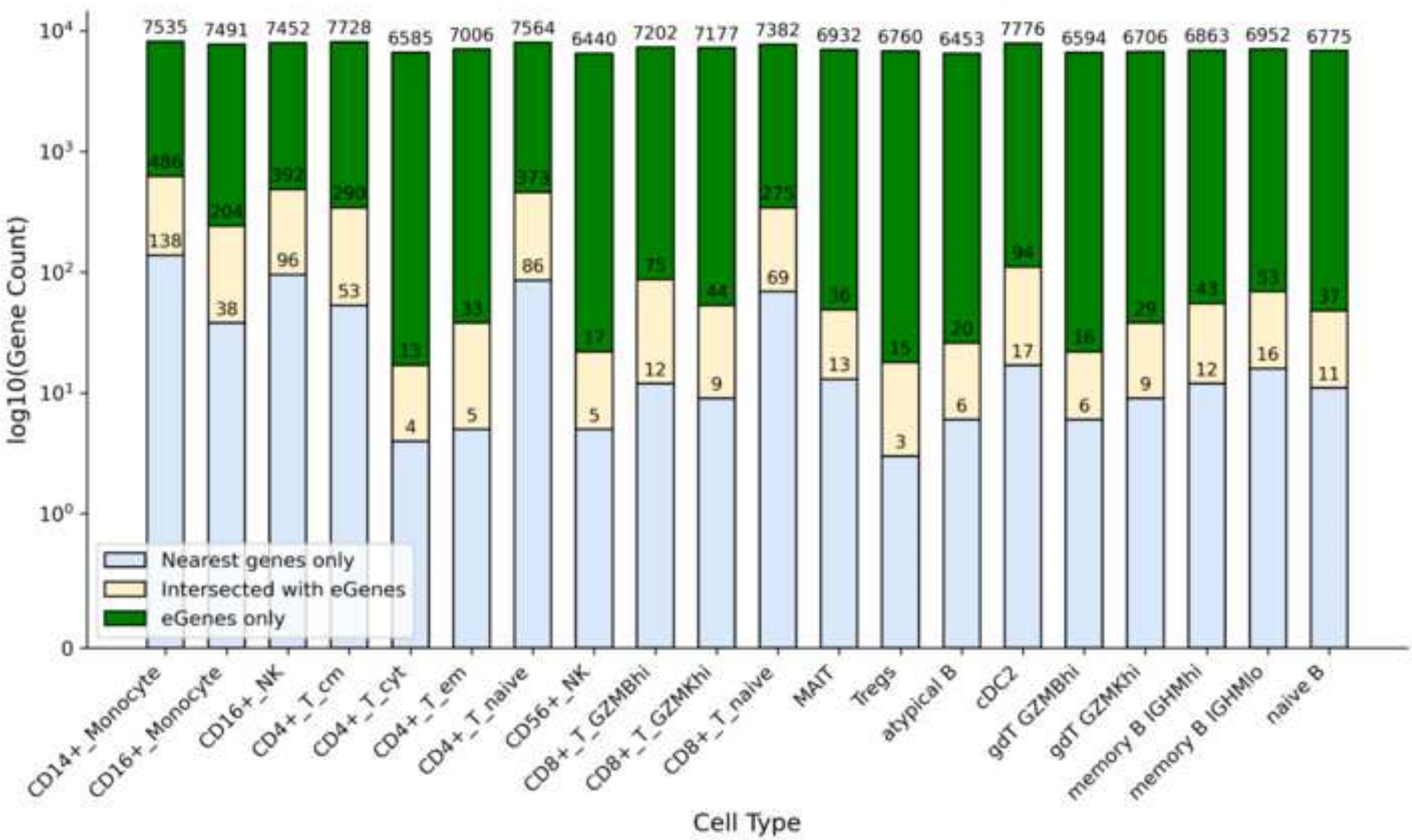

Reviewer #1:

This manuscript presents NEXT-scASV, a scalable and reproducible computational pipeline for the novo detection of Allele-Specific Variants (ASVs) from 5' single-cell RNA sequencing data. The pipeline integrates several well-established tools, including HISAT2, WASP, bcftools, and the advanced statistical framework MIXALIME, into a containerized Nextflow workflow. The authors demonstrate NEXT-scASV ability to efficiently process atlas-scale data and highlight its capacity to detect ASVs in rare cell populations and challenging genomic contexts, such as lncRNAs, which are frequently overlooked in bulk analyses. While this tool represents a valuable contribution to the computational genomics community, I have several concerns and suggestions that should be addressed to improve the manuscript.

1. In the Abstract, on line 2, I strongly recommend avoiding the term "imperfections" when referring to characteristics or biases in single-cell data. A more neutral and precise term would be more appropriate.

Answer:
Thank you for the recommendation. We changed the term “imperfections” to the more natural “characteristics”. Updated version: “The rapid accumulation of single-cell sequencing data presents major computational challenges in reproducibility, scaling, and handling data characteristics like sparsity.”

2. Although the pipeline uses containerization to ensure reproducibility, it is good practice to explicitly list all the major software tools used (e.g., cutadapt v5, HISAT2, UMI-tools, bcftools, WASP, MIXALIME) and their exact versions in the Methods section or at least in the GitHub repository or supplementary materials, to ensure full transparency.

Answer:
We totally agree, software versions are very important for reproducibility. We added major package versions to the supplementary data (Package versions). We also have an environment configuration file in our repository (as_env.yaml) with all the package versions, except WASP. WASP needs a patch, so we describe its installation in the README.

3. Regarding the selection of tools, I am concerned about the criteria used, particularly the choice of HISAT2 over other widely used aligners (such as STAR) for scRNA-seq, and bcftools over other variant callers such as GATK HaplotypeCaller. The authors should better justify these decisions, especially considering the known challenges and biases in RNA-seq variant calling, even in bulk data, which is substantially less sparse than single-cell datasets.

Answer:
We selected HISAT2 over STAR due to its superior speed, a critical factor when processing large single-cell datasets with multiple donors and cell types. This choice does not compromise accuracy: on a dataset with a natural human polymorphism rate of 0.001, both aligners delivered nearly identical performance, achieving near 100% precision and over 95% recall (Baruzzo et al. 2016).

The choice of variant caller determines the initial set of candidate SNPs and therefore establishes the upper bound for sensitivity and accuracy in downstream analysis. However, in our pipeline, the caller's impact is mitigated through several subsequent steps. We primarily use the caller for initial SNP discovery, after which we apply rigorous variant-level filters, including genotype quality (GQ), read depth (DP), and allelic depth (AD). Additionally, we implement read-level filters before counting, such as mapping quality (MAPQ), edit distance (NM), and exclusion of QC-failed reads using filter_reads.py and samtools (flag 512). Crucially, the final allele-specific expression (ASE) signal is derived from direct allele-specific read counting in BAM files using count_tags_pileup.py, rather than relying on caller-derived quality scores. Furthermore, variants are called at the patient level, which leverages information across related samples to address the sparse coverage typical of single-cell RNA-seq data. As a result, the final ASE estimates are substantially less dependent on the specific variant caller than in workflows where caller outputs directly determine allelic estimates. For this study, we selected bcftools as our variant caller, as it offers a simpler, faster, and more computationally lightweight solution while still providing robust candidate variants suitable for downstream ASE analysis.

We also add these rationales into the Methods: Alignment Subflow and Variant Calling Subflow sections.

4. In the Background section, the manuscript states that "very few existing tools allow for this type of analysis". However, tools such as SCALE and scBASE were developed with related goals. The authors should therefore compare NEXT-scASV with these existing approaches and clarify what advantages it provides.

Answer:
We thank the reviewer for the suggestion. While MixALime was extensively benchmarked in the original paper, including comparisons with other bulk-focused tools, benchmarking against single-cell methods is also important for assessing our pipeline. We agree that SCALE and scBASE address related questions, but they are not directly comparable to our setting and output. First, SCALE was developed primarily for gene-level allele-specific expression analysis (e.g., allelic expression per gene and cell-type/state), whereas NEXT-scASV focuses on variant-level allele-specific variants (ASVs). Because the input features, statistical targets, and outputs differ (gene-level vs. SNP-level events), a direct head-to-head comparison would not be methodologically meaningful. Second, scBASE requires phased variants/haplotypes to assign allelic counts to parental haplotypes and to perform its downstream analyses. In our setting, reliable phasing information is not available for the studied data, and NEXT-scASV is designed to operate without requiring phased genotypes, which is an important practical advantage. To provide a relevant comparison within the same variant-level scope, we instead evaluated two widely used tools with closely related goals, scDALI and DAESC. We find that scDALI provides a conservative subset of MixALime calls, while DAESC can expand the analysis by highlighting variants with context-dependent allelic effects. We add a new paragraph, "ASV tools comparison" to the results section with comparison details.

We revised the Background text to more precisely state that few existing tools enable variant-level ASV calling in scRNA-seq without requiring phased genotypes, and discuss SCALE and scBASE in this context:

"Despite these advantages, single-cell RNA-seq data are sparse: most variants are covered by few reads per cell, and many cells show dropout at a given locus. To increase power, some single-cell allele-specific approaches therefore rely on different aggregation strategies, most commonly at the gene level, where allelic counts are summed across sites within a gene and tested for gene-level ASE (Choi et al. 2019; Jiang et al. 2017). In this work, we focus on variant-level ASVs rather than gene-level aggregation, because regulatory effects are often driven by specific nucleotide changes. A single variant can disrupt a transcription factor motif, alter promoter or enhancer activity, or create a splice-altering event, leading to a mechanistic and directly interpretable signal. Variant-level results also enable direct overlap with external catalogs of regulatory variants, and they avoid masking situations where multiple variants within the same gene have different, context-dependent effects.
Several single-cell methods have been proposed for allele-specific analysis. SCALE (Jiang et al. 2017) is designed for gene-level ASE modeling from sparse scRNA-seq counts. scBASE (Choi et al. 2019) integrates allele-specific signals with phased haplotypes, which typically requires phased genotypes. Tools that can operate at the variant level using allelic counts: DAESC (Qi et al. 2023) and scDALI (Heinen et al. 2022). DAESC is formulated to detect differential allelic imbalance between groups or conditions, whereas scDALI supports tests for both differential effects (heterogeneous mode) and shared allele-specific effects (homogeneous mode), as well as a joint setting that combines information across modes.
"

5. MIXALIME is a key component of the pipeline, as it models allelic read counts using the Beta-Negative Binomial framework. However, the original MIXALIME methodology was primarily validated on bulk datasets including RNA-seq, CAGE-seq or ATAC-seq, as well as on simulated data, not on highly sparse single-cell data. Therefore, its direct applicability to scRNA-seq therefore requires additional justification. The manuscript would be strengthened by providing validation results specifically on single-cell data, or at least

a detailed explanation of why the method is expected to perform robustly in this context. This should include any modifications or parameter adjustments relative to the original implementation.

Answer:
In NEXT-scASV, MIXALIME is applied to allele-specific read counts aggregated into pseudo-bulk profiles for each sample-by-cell-type group, rather than to sparse per-cell matrices. This aggregation shields the model from single-cell dropout effects; residual scRNA-seq variability manifests primarily as overdispersion in read depth, which is explicitly captured by the Beta-Negative Binomial (BetaNB) framework. As orthogonal validation, we assessed the external support of detected signals: over 60% of significant ASVs overlap previously reported regulatory variants (Figure S1 B), including allele-specific transcription factor binding events from ADASTRA and cis-eQTLs from GTEx. These enrichments support the biological plausibility and robustness of our calls in the scRNA-seq context.

6. The results were validated for biological significance using the AIDA cohort study. On average, 82% of the ASV-associated genes identified by the pipeline were also reported in the original study. While this suggests good specificity, the manuscript does not adequately discuss sensitivity. What proportion of total known eGenes is recovered by this approach? The authors also state that the remaining ~18% of genes "likely represent a mix of false positives and, more interestingly, true cell-type-specific regulatory events". Since the AIDA study also involved single-cell-level detection, the meaning of "cell type-specific regulatory events" should be clarified and contextualized.

Answer:
We agree that the overlap of ASV-associated genes with the original AIDA results primarily reflects specificity and that sensitivity should be discussed explicitly. In our current analysis, we intentionally processed only a subset of the AIDA cohort (≈50 out of ≈300 individuals), because the goal of this section was to validate the pipeline behavior (end-to-end calling, bias correction, and statistical modeling) rather than to provide a final biological catalog. As expected, this design yields a lower recovery of the full set of known eGenes: 1) statistical power is reduced due to smaller sample size, and 2) allele-specific approaches are intrinsically limited to loci with expressed heterozygous variants covered by reads, so regulatory variants acting through distal mechanisms or variants not captured in the transcribed/read-covered regions cannot be recovered by construction. Importantly, this does not undermine the utility of ASE/ASV analysis: ASE provides a within-individual, allele-contrast signal that is complementary to eQTL mapping and is particularly informative for detecting cis-regulatory effects in a cell-type-resolved manner while being less sensitive to between-individual confounders. We will add a sensitivity estimate (fraction of known eGenes recovered in our subset; shown in a new Supplementary figure S5) and clarify terminology.
By “cell-type-specific regulatory events” we mean genes showing significant allelic imbalance in a particular cell type (or cell-type group) but not in others, consistent with cell-type-restricted regulatory activity and/or differences in expression/coverage across cell types. Although the AIDA study also performed single-cell-level detection, cell-type specificity in our context refers specifically to differential presence/strength of allelic imbalance across annotated cell types within the same cohort, rather than to novelty relative to bulk catalogs; accordingly, the remaining ~18% likely comprises both false positives and bona fide cell-type-restricted effects that are underpowered or averaged out in global/aggregated analyses.

7. Importantly, I attempted to follow the GitHub instructions to install and run the pipeline, but I was unable to do so. The Docker build fails because line 13 of the Dockerfile specifies "RUN chmod +x /app/run_workflow.sh && chmod +x /app/run.sh && chmod +x /app/bin/nextflow" yet the files run_workflow.sh and run.sh do not exist in the repository. I am not sure whether this is a general issue or if additional steps are required, but this must be corrected — either by updating the repository or by clarifying the installation instructions.

Answer:
Thank you, we corrected the Dockerfile and updated the instructions.

8. Finally, I believe the pipeline would greatly benefit from the inclusion of a small test dataset and a brief tutorial. This would enable users to evaluate the workflow's functionality and performance before applying it to large datasets.

Answer:
We fully agree that a small test dataset and tutorial are important. We prepared a small subset of the public dataset that we used for evaluation, including data from 2 patients and 14 cell types. We provide a download link to this dataset and add a brief step by step tutorial so users can quickly test the workflow and check its performance before running it on large datasets.

Reviewer #2:

This manuscript introduces NEXT-scASV, a pipeline for detecting allele-specific variants (ASVs) from 5' single-cell RNA-seq data. The authors applied it to 57 South Korean donors from the Asian Immune Diversity Atlas (AIDA) dataset and demonstrated that the workflow can efficiently process large datasets within a feasible timeframe on a mid-sized compute cluster. They further validated the detected ASVs against previously reported eQTLs (Kock et al., 2025), supporting the biological relevance of the findings. The manuscript also highlights the pipeline's ability to detect signals in rare cell types. Both the data and analysis scripts are publicly available, which enhances transparency and reproducibility.
Minor issue: On page 13, line 9, Figure 3B is referenced but does not appear in the manuscript.
Formatting issue: The first paragraph of Discussion section is left-aligned and inconsistent with the formatting of the surrounding text.
Suggestion for improvement: The Background section could be strengthened by summarizing existing single-cell ASV detection models and briefly comparing them with MIXALIME (Buyan et al. 2025) and WASP tool (van de Geijn et al. 2015), which are used within the NEXT-scASV pipeline. This context would help better position the contribution of the current work within the landscape of single-cell allele-specific variation analysis.

On page 13, line 9, Figure 3B is referenced but does not appear in the manuscript.

Answer:
Corrected "Figure 3B" -> "Figure 4B"

The first paragraph of Discussion section is left-aligned and inconsistent with the formatting of the surrounding text.

Answer:
Fixed the alignment in the discussion section.

Suggestion for improvement: The Background section could be strengthened by summarizing existing single-cell ASV detection models and briefly comparing them with MIXALIME (Buyan et al. 2025) and WASP tool (van de Geijn et al. 2015), which are used within the NEXT-scASV pipeline. This context would help better position the contribution of the current work within the landscape of single-cell allele-specific variation analysis.

Answer:
Thank you for this suggestion. In the revised manuscript, we add a short overview of existing single-cell allele-specific models and clarify how they relate to the tools used in our pipeline. To provide empirical context, we include our benchmark against scDALI and DAESC, showing that scDALI recovered largely the same variants as MixALime, but in smaller numbers. In contrast, DAESC identified additional, tool-specific variants, as it appears complementary to our general speciality detection approach. We placed the detailed analysis in the paragraph "ASV tools comparison" of the results section.

Reviewer #3:

In this manuscript, Shevtsov et al. introduce NEXT-scASV, a scalable pipeline for amplicon sequence variant (ASV) calling from 5' scRNA-seq data. The authors validated NEXT-scASV using 135,000 peripheral blood

mononuclear cells (PBMCs) from 57 donors and demonstrated its capacity to identify ASVs within rare cell populations.

A major concern regarding this manuscript is the lack of comparative benchmarking against existing tools. Although the manuscript states that "very few existing tools allow for this kind of analysis," it does not provide a comparison of NEXT-scASV with any of these tools. Even if direct "apples-to-apples" comparisons prove challenging, the variant calling component should be benchmarked against other single-cell variant callers, such as cellsnp-lite and Monopogen.

Answer:
Thank you for raising this point. We agree that benchmarking is important. For benchmarking the ASV-calling component, the most relevant existing approaches are scDALI and DAESC, which directly test allele-specific signals using single-cell allelic counts. We therefore compared NEXT-scASV/MixALime-based ASV calls to scDALI and DAESC in two input regimes: (i) a pseudobulk setup matching our pipeline (sample × group aggregation) and (ii) a true single-cell setup using per-barcode allele counts. In pseudobulk mode, scDALI and DAESC largely overlapped with MixALime calls, while MixALime reported more ASVs, consistent with its focus on detecting overall allelic imbalance rather than differential effects. In true single-cell mode, scDALI and DAESC detected substantially more ASVs (DAESC: 31 → 188; scDALI: 23 → 130). DAESC reported additional unique variants, reflecting its sensitivity to context-dependent allelic effects, in contrast to MiXalime that aims to detect general allele-specific events. Importantly, ASVs detected by all three approaches showed substantial overlap with known regulatory variant resources (GTEx cis-eQTLs and ADASTRA), supporting the biological relevance of the calls.

In NEXT-scASV, the variant caller is mainly used to generate an initial set of candidate SNPs. The final allele-specific signal is derived from direct allele-specific read counting after alignment and bias correction (WASP), followed by additional variant- and read-level filtering and statistical testing in MixALime. As a result, the final set of significant ASVs is less dependent on the particular caller than in workflows where caller-derived genotypes directly define allelic estimates.
Monopogen achieves improved sensitivity by leveraging high-quality haplotypes and linkage disequilibrium information, which can substantially limit applicability when such reference resources are not available or not well matched to the cohort. In our setting, we did not have suitable haplotype/LD resources required for Monopogen, and therefore could not include it as a practical comparator.
To provide an empirical benchmark, we compared bcftools with cellsnp-lite. Cellsnp-lite reported more candidate variants, but most additional variants did not pass downstream ASV significance testing in MixALime. This suggests that cellsnp-lite is more sensitive to low-coverage variants, but this increased sensitivity does not translate into a clear advantage in terms of final significant ASVs (Figure S3). At the same time, each caller contributed a little fraction of caller-specific variants.

In the revised version, we (1) rewrite the statement “very few tools” by explicitly describing existing tools and their scope, (2) include the scDALI/DAESC benchmarking results in the Results:ASV tools comparison, and add the comparison with cellsnp-lite

Furthermore, could the authors elucidate why they opted to physically split FASTQ files instead of employing Read Groups within merged BAM files, a standard approach in handling multiplexed data for variant calling, such as in GATK workflows? If splitting is necessary for parallelization of bcftools in this context, the authors should discuss the rationale for choosing between virtual splitting and sequential processing, to prevent excessive input/output operations.

Answer:
We split FASTQ files because our workflow runs several steps per sample, including filtering, alignment, and allele counting, and these steps are much faster and simpler when each sample is processed as its own unit. Using read groups in one merged BAM is common in GATK-style variant calling, but in our case, we would still need to separate data by group for downstream counting and quality control, which would mean repeatedly scanning or extracting parts of a large BAM and reusing large indexes, often creating more total input-output and more file system pressure than an early split. Splitting once at the beginning creates smaller files, enables clean parallel processing with predictable memory and runtime per job, and avoids

contention when many tasks read the same big BAM at the same time. We therefore chose physical splitting as a practical trade-off: a small one-time input-output cost up front, but lower total input-output and shorter runtime across the full pipeline compared to virtual splitting or sequential processing.

A minor point is that this manuscript extensively emphasizes the advantages of 5' scRNA-seq; however, the majority of publicly available single-cell datasets utilize 3' scRNA-seq. The authors should consider discussing the potential limitations—if any—when applying their method to 3' scRNA-seq data, or explicitly state this limitation to appropriately set user expectations.

Answer:
5’ scRNA-seq data reads are enriched near the transcription start site. Therefore, it provides better coverage of regulatory regions and nearby heterozygous variants that can act as causal regulatory SNPs. When the same workflow is applied to 3 prime scRNA-seq data, the main limitation is that coverage is shifted to the transcript end, so many candidate regulatory variants will not be sequenced, and the number of informative SNPs per gene and per cell can be much lower. As a result, both sensitivity and power to detect allele-specific effects are expected to decrease, and it can be difficult to identify causal rSNPs and even to assess validity because 3 prime datasets often do not capture the variants that are present in external allele-specific variant resources. We will add this point to the manuscript to clarify that 3 prime data can still be processed, but results may be less complete
