# Supplementary material for: NEXT-scASV: a Nextflow pipeline for allele-specific variant calling from single-cell RNA-seq data
Source: Gigascience. 2026 Apr 6;15:giag042. doi: 10.1093/gigascience/giag042 (PMC13148397; doi:10.1093/gigascience/giag042)
Supplement: giag042_Authors_Response_To_Reviewer_Comments_original_submission [file giag042_authors_response_to_reviewer_comments_original_submission.pdf]

Author's Response To Reviewer Comments

Reviewer #1:

1. In the Abstract, on line 2, I strongly recommend avoiding the term "imperfections" when referring to characteristics or biases in single-cell data. A more neutral and precise term would be more appropriate.

*Answer:
Thank you for the recommendation. We changed the term “imperfections” to the more natural “characteristics”. Updated version: “The rapid accumulation of single-cell sequencing data presents major computational challenges in reproducibility, scaling, and handling data characteristics like sparsity.”

2. Although the pipeline uses containerization to ensure reproducibility, it is good practice to explicitly list all the major software tools used (e.g., cutadapt v5, HISAT2, UMI-tools, bcftools, WASP, MIXALIME) and their exact versions in the Methods section or at least in the GitHub repository or supplementary materials, to ensure full transparency.

*Answer:
We totally agree, software versions are very important for reproducibility. We added major package versions to the supplementary data (Package versions). We also have an environment configuration file in our repository (as_env.yaml) with all the package versions, except WASP. WASP needs a patch, so we describe its installation in the README.

3. Regarding the selection of tools, I am concerned about the criteria used, particularly the choice of HISAT2 over other widely used aligners (such as STAR) for scRNA-seq, and bcftools over other variant callers such as GATK HaplotypeCaller. The authors should better justify these decisions, especially considering the known challenges and biases in RNA-seq variant calling, even in bulk data, which is substantially less sparse than single-cell datasets.

*Answer:
We selected HISAT2 over STAR due to its superior speed, a critical factor when processing large single-cell datasets with multiple donors and cell types. This choice does not compromise accuracy: on a dataset with a natural human polymorphism rate of 0.001, both aligners delivered nearly identical performance, achieving near 100% precision and over 95% recall (Baruzzo et al. 2016).

The choice of variant caller determines the initial set of candidate SNPs and therefore establishes the upper bound for sensitivity and accuracy in downstream analysis. However, in our pipeline, the caller's impact is mitigated through several subsequent steps. We primarily use the caller for initial SNP discovery, after which we apply rigorous variant-level filters, including genotype quality (GQ), read depth (DP), and allelic depth (AD). Additionally, we implement read-level filters before counting, such as mapping quality (MAPQ), edit distance (NM), and exclusion of QC-failed reads using filter_reads.py and samtools (flag 512). Crucially, the final allele-specific expression (ASE) signal is derived from direct allele-specific read counting in BAM files using count_tags_pileup.py, rather than relying on caller-derived quality scores. Furthermore, variants are called at the patient level, which leverages information across related samples to address the sparse coverage typical of single-cell RNA-seq data. As a result, the final ASE estimates are substantially less dependent on the specific variant caller than in workflows where caller outputs directly determine allelic estimates. For this study, we selected bcftools as our variant caller, as it offers a simpler, faster, and more computationally lightweight solution while still providing robust candidate variants suitable for downstream ASE analysis.

We also add these rationales into the Methods: Alignment Subflow and Variant Calling Subflow sections.

4. In the Background section, the manuscript states that "very few existing tools allow for this type of

analysis". However, tools such as SCALE and scBASE were developed with related goals. The authors should therefore compare NEXT-scASV with these existing approaches and clarify what advantages it provides.

*Answer:
We thank the reviewer for the suggestion. While MixALime was extensively benchmarked in the original paper, including comparisons with other bulk-focused tools, benchmarking against single-cell methods is also important for assessing our pipeline. We agree that SCALE and scBASE address related questions, but they are not directly comparable to our setting and output. First, SCALE was developed primarily for gene-level allele-specific expression analysis (e.g., allelic expression per gene and cell-type/state), whereas NEXT-scASV focuses on variant-level allele-specific variants (ASVs). Because the input features, statistical targets, and outputs differ (gene-level vs. SNP-level events), a direct head-to-head comparison would not be methodologically meaningful. Second, scBASE requires phased variants/haplotypes to assign allelic counts to parental haplotypes and to perform its downstream analyses. In our setting, reliable phasing information is not available for the studied data, and NEXT-scASV is designed to operate without requiring phased genotypes, which is an important practical advantage. To provide a relevant comparison within the same variant-level scope, we instead evaluated two widely used tools with closely related goals, scDALI and DAESC. We find that scDALI provides a conservative subset of MixALime calls, while DAESC can expand the analysis by highlighting variants with context-dependent allelic effects. We add a new paragraph, “ASV tools comparison” to the results section with comparison details.

We revised the Background text to more precisely state that few existing tools enable variant-level ASV calling in scRNA-seq without requiring phased genotypes, and discuss SCALE and scBASE in this context:

“Despite these advantages, single-cell RNA-seq data are sparse: most variants are covered by few reads per cell, and many cells show dropout at a given locus. To increase power, some single-cell allele-specific approaches therefore rely on different aggregation strategies, most commonly at the gene level, where allelic counts are summed across sites within a gene and tested for gene-level ASE (Choi et al. 2019; Jiang et al. 2017). In this work, we focus on variant-level ASVs rather than gene-level aggregation, because regulatory effects are often driven by specific nucleotide changes. A single variant can disrupt a transcription factor motif, alter promoter or enhancer activity, or create a splice-altering event, leading to a mechanistic and directly interpretable signal. Variant-level results also enable direct overlap with external catalogs of regulatory variants, and they avoid masking situations where multiple variants within the same gene have different, context-dependent effects.
Several single-cell methods have been proposed for allele-specific analysis. SCALE (Jiang et al. 2017) is designed for gene-level ASE modeling from sparse scRNA-seq counts. scBASE (Choi et al. 2019) integrates allele-specific signals with phased haplotypes, which typically requires phased genotypes. Tools that can operate at the variant level using allelic counts: DAESC (Qi et al. 2023) and scDALI (Heinen et al. 2022). DAESC is formulated to detect differential allelic imbalance between groups or conditions, whereas scDALI supports tests for both differential effects (heterogeneous mode) and shared allele-specific effects (homogeneous mode), as well as a joint setting that combines information across modes.
”

5. MIXALIME is a key component of the pipeline, as it models allelic read counts using the Beta-Negative Binomial framework. However, the original MIXALIME methodology was primarily validated on bulk datasets including RNA-seq, CAGE-seq or ATAC-seq, as well as on simulated data, not on highly sparse single-cell data. Therefore, its direct applicability to scRNA-seq therefore requires additional justification. The manuscript would be strengthened by providing validation results specifically on single-cell data, or at least a detailed explanation of why the method is expected to perform robustly in this context. This should include any modifications or parameter adjustments relative to the original implementation.

*Answer:
In NEXT-scASV, MIXALIME is applied to allele-specific read counts aggregated into pseudo-bulk profiles for each sample-by-cell-type group, rather than to sparse per-cell matrices. This aggregation shields the model from single-cell dropout effects; residual scRNA-seq variability manifests primarily as overdispersion

in read depth, which is explicitly captured by the Beta-Negative Binomial (BetaNB) framework. As orthogonal validation, we assessed the external support of detected signals: over 60% of significant ASVs overlap previously reported regulatory variants (Figure S1 B), including allele-specific transcription factor binding events from ADASTRA and cis-eQTLs from GTEx. These enrichments support the biological plausibility and robustness of our calls in the scRNA-seq context.

6. The results were validated for biological significance using the AIDA cohort study. On average, 82% of the ASV-associated genes identified by the pipeline were also reported in the original study. While this suggests good specificity, the manuscript does not adequately discuss sensitivity. What proportion of total known eGenes is recovered by this approach? The authors also state that the remaining ~18% of genes "likely represent a mix of false positives and, more interestingly, true cell-type-specific regulatory events". Since the AIDA study also involved single-cell-level detection, the meaning of "cell type-specific regulatory events" should be clarified and contextualized.

*Answer:
We agree that the overlap of ASV-associated genes with the original AIDA results primarily reflects specificity and that sensitivity should be discussed explicitly. In our current analysis, we intentionally processed only a subset of the AIDA cohort (≈50 out of ≈300 individuals), because the goal of this section was to validate the pipeline behavior (end-to-end calling, bias correction, and statistical modeling) rather than to provide a final biological catalog. As expected, this design yields a lower recovery of the full set of known eGenes: 1) statistical power is reduced due to smaller sample size, and 2) allele-specific approaches are intrinsically limited to loci with expressed heterozygous variants covered by reads, so regulatory variants acting through distal mechanisms or variants not captured in the transcribed/read-covered regions cannot be recovered by construction. Importantly, this does not undermine the utility of ASE/ASV analysis: ASE provides a within-individual, allele-contrast signal that is complementary to eQTL mapping and is particularly informative for detecting cis-regulatory effects in a cell-type-resolved manner while being less sensitive to between-individual confounders. We will add a sensitivity estimate (fraction of known eGenes recovered in our subset; shown in a new Supplementary figure S5) and clarify terminology.
By "cell-type-specific regulatory events" we mean genes showing significant allelic imbalance in a particular cell type (or cell-type group) but not in others, consistent with cell-type-restricted regulatory activity and/or differences in expression/coverage across cell types. Although the AIDA study also performed single-cell-level detection, cell-type specificity in our context refers specifically to differential presence/strength of allelic imbalance across annotated cell types within the same cohort, rather than to novelty relative to bulk catalogs; accordingly, the remaining ~18% likely comprises both false positives and bona fide cell-type-restricted effects that are underpowered or averaged out in global/aggregated analyses.

7. Importantly, I attempted to follow the GitHub instructions to install and run the pipeline, but I was unable to do so. The Docker build fails because line 13 of the Dockerfile specifies "RUN chmod +x /app/run_workflow.sh && chmod +x /app/run.sh && chmod +x /app/bin/nextflow" yet the files run_workflow.sh and run.sh do not exist in the repository. I am not sure whether this is a general issue or if additional steps are required, but this must be corrected — either by updating the repository or by clarifying the installation instructions.

*Answer:
Thank you, we corrected the Dockerfile and updated the instructions.

8. Finally, I believe the pipeline would greatly benefit from the inclusion of a small test dataset and a brief tutorial. This would enable users to evaluate the workflow's functionality and performance before applying it to large datasets.

*Answer:
We fully agree that a small test dataset and tutorial are important. We prepared a small subset of the public dataset that we used for evaluation, including data from 2 patients and 14 cell types. We provide a download link to this dataset and add a brief step by step tutorial so users can quickly test the workflow and check its performance before running it on large datasets.

Reviewer #2:

1. On page 13, line 9, Figure 3B is referenced but does not appear in the manuscript.

*Answer:
Corrected “Figure 3B” -> “Figure 4B”

2. The first paragraph of Discussion section is left-aligned and inconsistent with the formatting of the surrounding text.

*Answer:
Fixed the alignment in the discussion section.

3. Suggestion for improvement: The Background section could be strengthened by summarizing existing single-cell ASV detection models and briefly comparing them with MIXALIME (Buyan et al. 2025) and WASP tool (van de Geijn et al. 2015), which are used within the NEXT-scASV pipeline. This context would help better position the contribution of the current work within the landscape of single-cell allele-specific variation analysis.

*Answer:
Thank you for this suggestion. In the revised manuscript, we add a short overview of existing single-cell allele-specific models and clarify how they relate to the tools used in our pipeline. To provide empirical context, we include our benchmark against scDALI and DAESC, showing that scDALI recovered largely the same variants as MixALime, but in smaller numbers. In contrast, DAESC identified additional, tool-specific variants, as it appears complementary to our general speciality detection approach. We placed the detailed analysis in the paragraph “ASV tools comparison” of the results section.

Reviewer #3:

1. A major concern regarding this manuscript is the lack of comparative benchmarking against existing tools. Although the manuscript states that "very few existing tools allow for this kind of analysis," it does not provide a comparison of NEXT-scASV with any of these tools. Even if direct "apples-to-apples" comparisons prove challenging, the variant calling component should be benchmarked against other single-cell variant callers, such as cellsnp-lite and Monopogen.

*Answer:
Thank you for raising this point. We agree that benchmarking is important. For benchmarking the ASV-calling component, the most relevant existing approaches are scDALI and DAESC, which directly test allele-specific signals using single-cell allelic counts. We therefore compared NEXT-scASV/MixALime-based ASV calls to scDALI and DAESC in two input regimes: (i) a pseudobulk setup matching our pipeline (sample × group aggregation) and (ii) a true single-cell setup using per-barcode allele counts. In pseudobulk mode, scDALI and DAESC largely overlapped with MixALime calls, while MixALime reported more ASVs, consistent with its focus on detecting overall allelic imbalance rather than differential effects. In true single-cell mode, scDALI and DAESC detected substantially more ASVs (DAESC: 31 → 188; scDALI: 23 → 130). DAESC reported additional unique variants, reflecting its sensitivity to context-dependent allelic effects, in contrast to MiXalime that aims to detect general allele-specific events. Importantly, ASVs detected by all three approaches showed substantial overlap with known regulatory variant resources (GTEx cis-eQTLs and ADASTRA), supporting the biological relevance of the calls.

In NEXT-scASV, the variant caller is mainly used to generate an initial set of candidate SNPs. The final allele-specific signal is derived from direct allele-specific read counting after alignment and bias correction (WASP), followed by additional variant- and read-level filtering and statistical testing in MixALime. As a result, the final set of significant ASVs is less dependent on the particular caller than in workflows where

caller-derived genotypes directly define allelic estimates.
Monopogen achieves improved sensitivity by leveraging high-quality haplotypes and linkage disequilibrium information, which can substantially limit applicability when such reference resources are not available or not well matched to the cohort. In our setting, we did not have suitable haplotype/LD resources required for Monopogen, and therefore could not include it as a practical comparator.
To provide an empirical benchmark, we compared bcftools with cellsnp-lite. Cellsnp-lite reported more candidate variants, but most additional variants did not pass downstream ASV significance testing in MixALime. This suggests that cellsnp-lite is more sensitive to low-coverage variants, but this increased sensitivity does not translate into a clear advantage in terms of final significant ASVs (Figure S3). At the same time, each caller contributed a little fraction of caller-specific variants.

In the revised version, we (1) rewrite the statement “very few tools” by explicitly describing existing tools and their scope, (2) include the scDALI/DAESC benchmarking results in the Results:ASV tools comparison, and add the comparison with cellsnp-lite

2. Furthermore, could the authors elucidate why they opted to physically split FASTQ files instead of employing Read Groups within merged BAM files, a standard approach in handling multiplexed data for variant calling, such as in GATK workflows? If splitting is necessary for parallelization of bcftools in this context, the authors should discuss the rationale for choosing between virtual splitting and sequential processing, to prevent excessive input/output operations.

*Answer:
We split FASTQ files because our workflow runs several steps per sample, including filtering, alignment, and allele counting, and these steps are much faster and simpler when each sample is processed as its own unit. Using read groups in one merged BAM is common in GATK-style variant calling, but in our case, we would still need to separate data by group for downstream counting and quality control, which would mean repeatedly scanning or extracting parts of a large BAM and reusing large indexes, often creating more total input-output and more file system pressure than an early split. Splitting once at the beginning creates smaller files, enables clean parallel processing with predictable memory and runtime per job, and avoids contention when many tasks read the same big BAM at the same time. We therefore chose physical splitting as a practical trade-off: a small one-time input-output cost up front, but lower total input-output and shorter runtime across the full pipeline compared to virtual splitting or sequential processing.

3. A minor point is that this manuscript extensively emphasizes the advantages of 5' scRNA-seq; however, the majority of publicly available single-cell datasets utilize 3' scRNA-seq. The authors should consider discussing the potential limitations—if any—when applying their method to 3' scRNA-seq data, or explicitly state this limitation to appropriately set user expectations.

*Answer:
5’ scRNA-seq data reads are enriched near the transcription start site. Therefore, it provides better coverage of regulatory regions and nearby heterozygous variants that can act as causal regulatory SNPs. When the same workflow is applied to 3 prime scRNA-seq data, the main limitation is that coverage is shifted to the transcript end, so many candidate regulatory variants will not be sequenced, and the number of informative SNPs per gene and per cell can be much lower. As a result, both sensitivity and power to detect allele-specific effects are expected to decrease, and it can be difficult to identify causal rSNPs and even to assess validity because 3 prime datasets often do not capture the variants that are present in external allele-specific variant resources. We will add this point to the manuscript to clarify that 3 prime data can still be processed, but results may be less complete

Editorial Manager®

Cookie Preference Center

We use cookies which are necessary to make our site work. We may also use additional cookies to analyze, improve and personalize our content and your digital experience. For more information, see our Cookie Policy.

You may choose not to allow some types of cookies. However, blocking some types may impact your experience of our site and the services we are able to offer. See the different category headings below to find out more or change your settings.

Allow all

Manage Consent Preferences

Strictly Necessary Cookies

Always active

These cookies are necessary for the website to function and cannot be switched off in our systems. They are usually only set in response to actions made by you which amount to a request for services, such as setting your privacy preferences, logging in or filling in forms. You can set your browser to block or alert you about these cookies, but some parts of the site will not then work.

Functional Cookies

These cookies enable the website to provide enhanced functionality and personalisation. They may be set by us or by third party providers whose services we have added to our pages. If you do not allow these cookies then some or all of these services may not function properly.

Performance Cookies

These cookies allow us to count visits and traffic sources so we can measure and improve the performance of our site. They help us to know which pages are the most and least popular and see how visitors move around the site.

Targeting Cookies

These cookies may be set through our site by our advertising partners. They may be used by those companies to build a profile of your interests and show you relevant adverts on other sites. If you do not allow these cookies, you will experience less targeted advertising.

Cookie List

Clear

Apply Cancel

Consent Leg.Interest

Confirm my choices

Power
