# Supplementary material for: NEXT-scASV: a Nextflow pipeline for allele-specific variant calling from single-cell RNA-seq data
Source: Gigascience. 2026 Apr 6;15:giag042. doi: 10.1093/gigascience/giag042 (PMC13148397; doi:10.1093/gigascience/giag042)
Supplement: giag042_Reviewer_1_Report_original_submission — Reviewer 1 -- 11/20/2025 [file giag042_reviewer_1_report_original_submission.pdf]

Reviewer Report

Title: NEXT-scASV: A Nextflow Pipeline for Allele-Specific Variants Calling from single cell RNA-seq data

Version: Original Submission **Date:** 11/20/2025

Reviewer name: Reviewer 1

Reviewer Comments to Author:

This manuscript presents NEXT-scASV, a scalable and reproducible computational pipeline for the novo detection of Allele-Specific Variants (ASVs) from 5' single-cell RNA sequencing data. The pipeline integrates several well-established tools, including HISAT2, WASP, bcftools, and the advanced statistical framework MIXALIME, into a containerized Nextflow workflow. The authors demonstrate NEXT-scASV ability to efficiently process atlas-scale data and highlight its capacity to detect ASVs in rare cell populations and challenging genomic contexts, such as lncRNAs, which are frequently overlooked in bulk analyses. While this tool represents a valuable contribution to the computational genomics community, I have several concerns and suggestions that should be addressed to improve the manuscript.

1. In the Abstract, on line 2, I strongly recommend avoiding the term "imperfections" when referring to characteristics or biases in single-cell data. A more neutral and precise term would be more appropriate.
2. Although the pipeline uses containerization to ensure reproducibility, it is good practice to explicitly list all the major software tools used (e.g., cutadapt v5, HISAT2, UMI-tools, bcftools, WASP, MIXALIME) and their exact versions in the Methods section or at least in the GitHub repository or supplementary materials, to ensure full transparency.
3. Regarding the selection of tools, I am concerned about the criteria used, particularly the choice of HISAT2 over other widely used aligners (such as STAR) for scRNA-seq, and bcftools over other variant callers such as GATK HaplotypeCaller. The authors should better justify these decisions, especially considering the known challenges and biases in RNA-seq variant calling, even in bulk data, which is substantially less sparse than single-cell datasets.
4. In the Background section, the manuscript states that "very few existing tools allow for this type of analysis". However, tools such as SCALE and scBASE were developed with related goals. The authors should therefore compare NEXT-scASV with these existing approaches and clarify what advantages it provides.
5. MIXALIME is a key component of the pipeline, as it models allelic read counts using the Beta-Negative Binomial framework. However, the original MIXALIME methodology was primarily validated on bulk datasets including RNA-seq, CAGE-seq or ATAC-seq, as well as on simulated data, not on highly sparse single-cell data. Therefore, its direct applicability to scRNA-seq therefore requires additional justification. The manuscript would be strengthened by providing validation results specifically on single-cell data, or at least a detailed explanation of why the method is expected to perform robustly in this context. This should include any modifications or parameter adjustments relative to the original implementation.
6. The results were validated for biological significance using the AIDA cohort study. On average, 82% of

the ASV-associated genes identified by the pipeline were also reported in the original study. While this suggests good specificity, the manuscript does not adequately discuss sensitivity. What proportion of total known eGenes is recovered by this approach? The authors also state that the remaining ~18% of genes "likely represent a mix of false positives and, more interestingly, true cell-type-specific regulatory events". Since the AIDA study also involved single-cell-level detection, the meaning of "cell type-specific regulatory events" should be clarified and contextualized.
7. Importantly, I attempted to follow the GitHub instructions to install and run the pipeline, but I was unable to do so. The Docker build fails because line 13 of the Dockerfile specifies "RUN chmod +x /app/run_workflow.sh && chmod +x /app/run.sh && chmod +x /app/bin/nextflow" yet the files run_workflow.sh and run.sh do not exist in the repository. I am not sure whether this is a general issue or if additional steps are required, but this must be corrected â€” either by updating the repository or by clarifying the installation instructions.
8. Finally, I believe the pipeline would greatly benefit from the inclusion of a small test dataset and a brief tutorial. This would enable users to evaluate the workflow's functionality and performance before applying it to large datasets.

Methods

Are the methods appropriate to the aims of the study, are they well described, and are necessary controls included? Choose an item.

Conclusions

Are the conclusions adequately supported by the data shown? Choose an item.

Reporting Standards

Does the manuscript adhere to the journal’s guidelines on [minimum standards of reporting?] Choose an item.

Choose an item.

Statistics

Are you able to assess all statistics in the manuscript, including the appropriateness of statistical tests used? Choose an item.

Quality of Written English

Please indicate the quality of language in the manuscript: Choose an item.

Declaration of Competing Interests

Please complete a declaration of competing interests, considering the following questions:

- Have you in the past five years received reimbursements, fees, funding, or salary from an organisation that may in any way gain or lose financially from the publication of this manuscript, either now or in the future?
- Do you hold any stocks or shares in an organisation that may in any way gain or lose financially from the publication of this manuscript, either now or in the future?
- Do you hold or are you currently applying for any patents relating to the content of the manuscript?
- Have you received reimbursements, fees, funding, or salary from an organization that holds or has applied for patents relating to the content of the manuscript?
- Do you have any other financial competing interests?
- Do you have any non-financial competing interests in relation to this paper?

If you can answer no to all of the above, write 'I declare that I have no competing interests' below. If your reply is yes to any, please give details below.

I declare that I have no competing interests

I agree to the open peer review policy of the journal. I understand that my name will be included on my report to the authors and, if the manuscript is accepted for publication, my named report including any attachments I upload will be posted on the website along with the authors' responses. I agree for my report to be made available under an Open Access Creative Commons CC-BY license (http://creativecommons.org/licenses/by/4.0/). I understand that any comments which I do not wish to be included in my named report can be included as confidential comments to the editors, which will not be published.

Choose an item.

To further support our reviewers, we have joined with Publons, where you can gain additional credit to further highlight your hard work (see: https://publons.com/journal/530/gigascience). On publication of this paper, your review will be automatically added to Publons, you can then choose whether or not to claim your Publons credit. I understand this statement.

Yes Choose an item.
