# Supplementary material for: NEXT-scASV: a Nextflow pipeline for allele-specific variant calling from single-cell RNA-seq data
Source: Gigascience. 2026 Apr 6;15:giag042. doi: 10.1093/gigascience/giag042 (PMC13148397; doi:10.1093/gigascience/giag042)
Supplement: giag042_Reviewer_3_Report_original_submission — Reviewer 3 -- 11/26/2025 [file giag042_reviewer_3_report_original_submission.pdf]

Reviewer Report

Title: NEXT-scASV: A Nextflow Pipeline for Allele-Specific Variants Calling from single cell RNA-seq data

Version: Original Submission **Date:** 11/26/2025

Reviewer name: Reviewer 3

Reviewer Comments to Author:

In this manuscript, Shevtsov et al. introduce NEXT-scASV, a scalable pipeline for amplicon sequence variant (ASV) calling from 5' scRNA-seq data. The authors validated NEXT-scASV using 135,000 peripheral blood mononuclear cells (PBMCs) from 57 donors and demonstrated its capacity to identify ASVs within rare cell populations.
A major concern regarding this manuscript is the lack of comparative benchmarking against existing tools. Although the manuscript states that "very few existing tools allow for this kind of analysis," it does not provide a comparison of NEXT-scASV with any of these tools. Even if direct "apples-to-apples" comparisons prove challenging, the variant calling component should be benchmarked against other single-cell variant callers, such as cellsnp-lite and Monopogen.
Furthermore, could the authors elucidate why they opted to physically split FASTQ files instead of employing Read Groups within merged BAM files, a standard approach in handling multiplexed data for variant calling, such as in GATK workflows? If splitting is necessary for parallelization of bcftools in this context, the authors should discuss the rationale for choosing between virtual splitting and sequential processing, to prevent excessive input/output operations.
A minor point is that this manuscript extensively emphasizes the advantages of 5' scRNA-seq; however, the majority of publicly available single-cell datasets utilize 3' scRNA-seq. The authors should consider discussing the potential limitationsâ€"if anyâ€"when applying their method to 3' scRNA-seq data, or explicitly state this limitation to appropriately set user expectations.

Methods

Are the methods appropriate to the aims of the study, are they well described, and are necessary controls included? Choose an item.

Conclusions

Are the conclusions adequately supported by the data shown? Choose an item.

Reporting Standards

Does the manuscript adhere to the journal's guidelines on minimum standards of reporting? Choose an item.

Choose an item.

Statistics

Are you able to assess all statistics in the manuscript, including the appropriateness of statistical tests used? Choose an item.

Quality of Written English

Please indicate the quality of language in the manuscript: Choose an item.

Declaration of Competing Interests

Please complete a declaration of competing interests, considering the following questions:

- Have you in the past five years received reimbursements, fees, funding, or salary from an organisation that may in any way gain or lose financially from the publication of this manuscript, either now or in the future?
- Do you hold any stocks or shares in an organisation that may in any way gain or lose financially from the publication of this manuscript, either now or in the future?
- Do you hold or are you currently applying for any patents relating to the content of the manuscript?
- Have you received reimbursements, fees, funding, or salary from an organization that holds or has applied for patents relating to the content of the manuscript?
- Do you have any other financial competing interests?
- Do you have any non-financial competing interests in relation to this paper?

If you can answer no to all of the above, write 'I declare that I have no competing interests' below. If your reply is yes to any, please give details below.

I declare that I have no competing interests

I agree to the open peer review policy of the journal. I understand that my name will be included on my report to the authors and, if the manuscript is accepted for publication, my named report including any attachments I upload will be posted on the website along with the authors' responses. I agree for my report to be made available under an Open Access Creative Commons CC-BY license (http://creativecommons.org/licenses/by/4.0/). I understand that any comments which I do not wish to be included in my named report can be included as confidential comments to the editors, which will not be published.

Choose an item.

To further support our reviewers, we have joined with Publons, where you can gain additional credit to further highlight your hard work (see: https://publons.com/journal/530/gigascience). On publication of this paper, your review will be automatically added to Publons, you can then choose whether or not to claim your Publons credit. I understand this statement.

Yes Choose an item.
